# Reaction of oxiranes with cyclodextrins under high-energy ball-milling conditions

**DOI:** 10.3762/bjoc.15.145

**Published:** 2019-07-01

**Authors:** László Jicsinszky, Federica Calsolaro, Katia Martina, Fabio Bucciol, Maela Manzoli, Giancarlo Cravotto

**Affiliations:** 1 Dipartimento di Scienza e Tecnologia del Farmaco, University of Turin, via P. Giuria 9, 10125 Turin, Italy

**Keywords:** crosslinked cyclodextrin polymers, (2-hydroxy)propylcyclodextrin, mechanochemistry, nucleophile reaction, planetary ball mill, solventless synthesis

## Abstract

This work presents a proof of concept for a green cyclodextrin derivatisation method that uses low-boiling epoxide reagents in a high-energy ball mill (HEBM). The simplified preparation and purification of low substitution-degree common (2-hydroxy)propylated β- and γ-cyclodextrins (β/γ-CDs) has been realised. The intelligent use of propylene oxide has also facilitated the more effective synthesis of highly substituted γ-CD. Epichlorohydrin-crosslinked CD-polymers (CDPs) have also been effectively prepared in the ball mill. The unoptimised preparations of soluble and insoluble CDPs displayed very small particle size distributions, while the prepared polymers currently have different complexation properties to those of their classically prepared analogues.

## Introduction

The derivatisation of natural cyclodextrins (CDs, cyclic α(1->4)-linked glucopyranosides) is always a difficult task, particularly when the attached moiety is prone to further derivatisation. In many cases, statistical (random) substitution results in essential changes in the hydrogen bonding systems of the cyclodextrin hydroxy rims, and is especially useful when the crystalline complex formation with the guest is undesirable. The aggregation properties, by the ready-to-associate secondary rim, are considerably affected by these substituents. One of the most important reactions of CDs occurs with oxiranes (epoxides) under basic conditions to provide (2-hydroxy)propyl-CDs (HPCDs) that are commonly used in pharmaceutical formulations and household products. Furthermore, a class of non-hydrolysable, soluble and insoluble CD polymers (CDP) can be prepared by crosslinking CDs with epichlorohydrin or 1,2:3,4-diepoxybutane. These are the oldest and most commonly used CDPs [1], more so than the so-called “nanosponges” [2], as well as being more chemically stable in both aqueous and alcoholic media.

Their industrial-scale preparation has been designed and uses sodium hydroxide as the base in a very concentrated aqueous CD solution [3]. The low solubility of β-CD can be dramatically increased using a base, which occurs via the ionisation of the secondary hydroxy groups, near pH ≈12 (secondary hydroxy dissociation starts around ≈11.5–11.8 in water) [4–5]. In basic aqueous medium, the high reactivity and low water-miscibility of 1,2-propylene oxide make the formation of (oligo)propylene glycol (oligo-PGs) unavoidable. Moreover, at higher degree of substitution (DS, number of substituent/macrocycle) the HP moiety can be further substituted, giving oligo-PG sidechains on the CDs [6]. At low DS values, occasional residual unsubstituted CDs can be removed (by aluminium oxide) and “recrystallisation” (from acetone) eliminates (oligo)-PG to a pharmaceutically acceptable level [6–7]. However, the low boiling point of the 1,2-propylene oxide generates some safety concerns. The low reaction temperature favours the secondary hydroxy group in CD substitution, which results in products having some DS-dependent solubilisation properties [8–11]. In solvent-free conditions, although the nature of the reaction means that they are not absolutely water free, not only the PG formation can be optimised but also the reagent use. As previously demonstrated, mechanochemical reactions usually give a more balanced substitution pattern for the randomly substituted derivatives [12].

In CDP preparations almost the same reaction conditions are used, but 1,2-propylene oxide is replaced with epichlorohydrin [13–16]. The disadvantage of this reaction is that the first step produces CD-propyl chlorohydrin, which is immediately transformed into an epoxide that can either react with other CDs, to form CDP, or with a hydroxy ion, to produce a (2,3-dihydroxy)propyl side chain. The hydrolytic reaction is unavoidable because of the aqueous solution and the higher reaction temperature. As the epichlorohydrin is in a large molar excess at the beginning of the reaction, its partial hydrolysis can result in the presence of glycerol moieties. Although it is true that the hydrolysis can be impeded somewhat by adding the reagents to the reaction mixture in a controlled manner, this side reaction cannot be completely eliminated.

The solubilisation/complexation potential of the polymers can be increased by the application of a chemically inert guest, using the so-called molecular imprinting strategy [17–19].

Despite the early patents and a promising beginning, CDPs are poorly applied CD derivatives and a standardised quality product is still missing. Detailed studies of the complexation properties of bead polymers have demonstrated their slow and structure-dependent complexation properties [13]. The preparation of an insoluble CDP is always challenging. Achieving a uniform insoluble CDP particle size is difficult and usually requires special, poorly transferable techniques [13–14,16,20]. The drawbacks of classical derivatisation methods include their problematic scale-up, which explains the limited number of sources of insoluble CDPs and also their high prices. Insoluble CDPs have excellent separation power in both regio- and enantiomer separations, as initially described by Zsadon et al., many decades ago [21–24]. More widespread use of CDPs in analytical and preparative applications is not only restricted by the aforementioned factors, but also by the lack of uniformity and the site-by-site variability of prepared CDPs, which have hampered the extensive CDP use.

Mechanochemistry has proven to be a useful green tool in the hands of synthetic chemists [25–28]. The reaction of epoxides with cyclodextrins under green and solventless conditions is discussed in this report. The HEBM technique was performed in a planetary ball mill for this purpose [12,29]. Proof of concept and reagent use were investigated for the most common HPCDs. Substitution patterns were not the target of our study as the modification of current industrial methods cannot be expected to occur in the near future. In the case of CDPs, our main aims were to find an effective, reproducible and green method for the preparation of a uniform insoluble CDP and to study its complexation properties in order to discover the right direction for synthesis optimisation.

## Results and Discussion

### Reaction of epoxides with CDs

The reactions of oxiranes, particularly propylene oxide, with CDs in solution have been extensively studied and the products are synthesised on the ton-scale by various companies [6,30–32]. The reaction is based on the activation of hydroxy groups with a base, which also increases CD solubility in water. Although the reaction proceeds in a completely random manner at a moderate molar ratio of base and low reaction temperatures, secondary hydroxy substitution is preferred. Primary OH and HP moiety substitution is suppressed at low DS values [9,33–36]. The aqueous solubility of propylene oxide is limited, meaning that only the dissolved reagent can react with the CD OH groups, which results in a permanent high excess of OH^−^. While the formation of a complex between propylene oxide and the CDs increases the solubility, OH^−^ can react with the reagent and the PG that is formed contaminates the product. Further reaction between propylene oxide, PG and the substituent(s) on the CDs leads to (oligo)PGs in the product and 2-(2-hydroxypropoxy)propyl moieties on the CDs. Although PG/oligo-PG is a pharmaceutically accepted solvent, its amount is limited, especially in parenteral applications [37]. Generally, 60–70% propylene oxide is utilized in the CD substitution reaction, which can result in 5–10% oligo-PG content in the crude product [6]. The hazardous nature of the reagent makes full conversion a necessity.

The substitution of propylene oxide with other reagents that are commonly used in CD derivatisation, such as 1,2:3,4-diepoxybutane and epichlorohydrin, further complicates the problems of reactions in solution. The hydrolysis of these reagents, whether in the free form or bound to CDs, results in a very complex structure that renders analyses and in-process control even more difficult. Furthermore, complications in reproducibility limit CDP use. Epichlorohydrin, which is used for the preparation of soluble CDPs, can also be used in the synthesis of insoluble CDPs. Although many kinds of insoluble CDPs [38], not only those based on epichlorohydrin, have been reported, their accessibility even on a kg-scale is very limited. The spread of insoluble polymer use can be easily improved by the existence of a readily transferable and engineered method, which would eliminate the main limiting factors of solution chemistry.

**Scheme 1:**
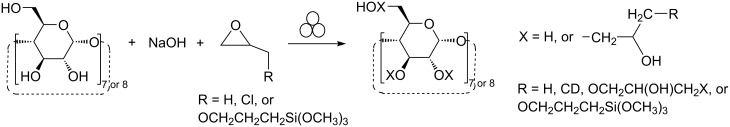
The reaction of CDs with oxiranes.

The restricted mobility of solid-state components further reduces the aforementioned side-reactions and enhances the utilization in the CD substitution. Although many side reactions are suppressed in ball milling, they cannot be completely eliminated when alkaline hydroxides are used to activate the CD OH groups. The formation of CD alkali salts always results in residual water when no metals or metal hydrides are used. The safer use of the hydroxides, in comparison with alkali metals or their hydrides, considerably outweighs the purification costs. Scheme 1 shows a general reaction scheme for CD substitution. This scheme is also valid for solution reactions.

### (*R*/*S*)-1,2-Epoxypropane ((±)-propylene oxide)

The solution method proceeded as a typical reaction run of hydroxypropylation; after a short period of intense stirring, a precipitate started to form (monoHP CDs), which slowly re-dissolved as the reaction mixture warmed and the reaction became faster. In order to allow the complete conversion of propylene oxide to occur, the reaction mixture was stirred for 4 days, after the ice in the bath had completely melted. The relatively large bath volume kept the reaction temperature practically constant at around room temperature (rt) during stirring.

Freshly dried CDs were generally used in ball-milling reactions to minimise the hydrolysis of propylene oxide, but we also tested CD-hydrates. Unlike in the solution method, HEBM experiments provide for relatively poor opportunities to visually inspect reaction progress. The low boiling point of propylene oxide means that the reaction-mixture temperature inside the jar cannot be monitored, but as our previous experiments have shown that monitoring the jar temperature, between certain limits, can give information about the energetics of the reaction [39]. The well-sealed jar is able to keep all the epoxides inside over the total milling time and, despite the necessity to have a considerable amount of propylene oxide in the vapour phase, the reagent also remains in the reaction vessel. In order to minimise the destruction of the reagent, which can occur either via hydrolysis by the residual water or escape via evaporation, the jar was cooled with liquid N_2_ below −30 °C after the sodium salt formation of the CDs and before the epoxide was added to the light, electrostatic powder. Liquid N_2_ not only worked as a cooling medium, but its evaporation completely excluded the humidity of air. Salt formation was exothermic, although the milling of CDs and NaOH also increased the internal temperature of the powder. Milling also increases the solid’s temperature, as was previously found to occur without a reagent. Whereas the internal temperature increased to around 40–45 °C when CDs were milled alone, the temperature of the powder could reach 60–65 °C during salt formation when the NaOH was added, while the external jar temperature stabilised at around 40–45 °C until the end of the milling periods.

**Figure 1:**
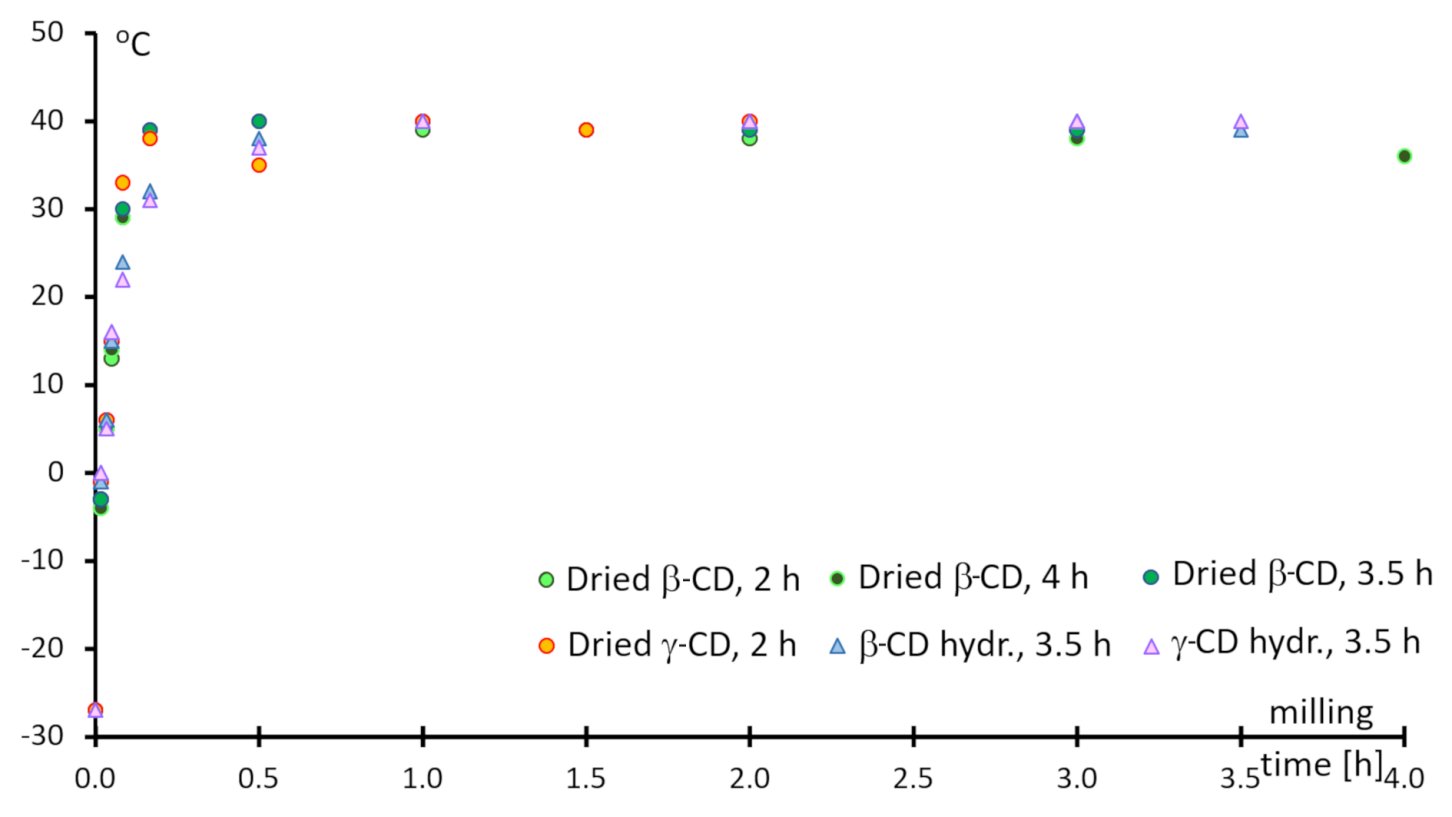
Jar-temperature changes during the reaction of 1,2-propylene oxide and cyclodextrins in the presence of NaOH.

As the propylene oxide was added, milling rapidly increased the jar temperature from less than −30 °C to between 40–45 °C. It then remained almost constant over 2 h of milling, as seen in Figure 1. The jar temperature reached rt in the first 5 minutes and the jar temperature started to decrease slowly after around 2 hours. The jar fixer needed to be adjusted often over the first minutes of milling due to its loosing as the system warmed.

As the jar temperature started to decrease after 2 hours, it was assumed that the reaction was finished. However, some hissing and powder blow-off upon jar opening indicated propylene-oxide overpressure, which signified its incomplete conversion. This effect was found in both β- and γ-CD reactions after 2 hours. The complete conversion of the reagent was found upon increasing the milling time to 4 hours. Finally, 3.5 h milling was found to completely convert the propylene oxide in cases with low DS values. The reaction mixtures were always in a fine powder state over the entire course of the reactions, including salt formation.

When using CD-hydrates, their natural state and as they are available from the suppliers, a hard CD-salt solid stuck to the jar wall in both β- and γ-CD, unlike with dried CDs. This solid should be removed from the wall to maximise reagent contact before cooling and the addition of propylene oxide. As the reaction proceeded, the solids became light and very electrostatic powders, just like in the dried CD tests. Product isolation was considerably easier than in the solution reactions because water elimination was not necessary. Any residual unsubstituted CDs were also removed and yields were quite acceptable, as seen in Table 1. Because the use of propylene oxide was found to be better here than in the solution reaction, higher DSs were obtained than would have been expected based on the solution reactions. Although the TLC of the powders after the reaction showed very little unsubstituted CD content, the removal of the very poorly soluble monoHPs also increased the average DS of the products.

**Table 1:** Summary of HPCD syntheses in HEBM.

No.	CD	Milling time [h]	B2M ratio^a^	Product^b^ [g]	DS^c^	Yield^d^ [%]

1	β	2	14.6	2.3	4.4	84
2	β	4	14.6	2.3	5.6	78
3	β	3.5	14.6	2.5	5.3	87
4	β-hydrate	3.5	13.0	2.4	3.7	89
5	γ	2	13.2	2.7	5.1	84
6	γ-hydrate	3.5	12.2	2.4	5.5	73

^a^Ball-to-mass (mass of balls/mass of reagents); ^b^isolated, purified; ^c^calculated from the integration of the anomeric-proton and CH_3_ signals of ^1^H NMR spectra and corrected using the residual solvent content; ^d^on the base of DS.

The easiest and cheapest product removal was dissolution in MeOH as nearly all of the components, except unsubstituted CDs and some monoHPs (these are somehow solubilized by the higher-DS HPCDs), are soluble in MeOH. At the end of the reaction, the MeOH contained some solids, but the filtration failed because of the very small particles. While centrifugation resulted in a clear solution, the clean removal of the supernatant was practically impossible because of re-suspension. Finally, due to the negligible amount of the fine powder, the suspension was treated immediately with a cation-exchanger to remove the sodium ion from the system. Some anion-exchanger was added before further treatment in order to remove the occasionally present fragments of the strong cation-exchanger. According to the supplier’s specifications, a few tenths of a per cent of linear dextrins (having reducing ends) cause strong colouration in the reaction mixture, in both the solid and solution methods. In order to reduce the colour intensity and remove the few insolubles, such as unsubstituted CDs, etc., from the solution, 5% charcoal was used.

The addition of acetone to the concentrated MeOH solution, to around 50% product content, completely removed the formed PGs. It was found that sometimes a small percentage of residual solvent was observed, despite the long drying times at temperatures above the solvent boiling points, as seen in the ^1^H NMR spectra. The DS of the product was calculated from the ^1^H NMR spectra, according to the pharmacopoeia method, using the integrals of the anomeric protons of the CDs and the integration of the methyl signal of the HP moiety (DS = Int_Me_/3, the anomeric proton signal integral is set to 7.0 or 8.0, according to the CD used). Similar calculations using the CD core-proton integral, which overlaps with the methine and methylene protons of the HP, resulted in similar DS values within the calculation limits. The lack of sharp signals in both the core- and methyl signal regions allowed us to assume that PGs were present in amounts that were below the detection limits. The mother liquors of the MeOH–acetone crystallisation of the products were hygroscopic and slightly waxy, which demonstrates their PG content.

A comparison of DS, by ^1^H NMR, and the product distribution of the ESIMS data showed a Gaussian-like distribution of masses in the range of 1–11, which was independent of the target DS. The ESIMS of the neutral HPCDs resulted in a complex spectral composition and showed mass distributions that were centred differently to the average DS that were calculated using ^1^H NMR.

The recovery of CD derivatives from the adsorbents showed minimal unsubstituted CDs and monoHP contents dominated, which explains the shift of DS toward the higher substitution range of the worked-up products. It is in agreement with previous findings [7].

The synthesis of high DS HP-γ-CD in solution is a multistep process used to minimise reagent loss and the formation of oligo-PGs during synthesis. The high DS and the oligo-PG side chain do not permit acetone crystallisation to remove oligo-PGs. Dialysis can completely remove PG contaminants but, particularly when DS < 10, leads to considerable product losses. High reagent utilisation in the reaction allowed the preparation of highly substituted CDs to be simplified and reduced the number of reaction steps, which could also cut down on purification as well as minimising residual PG impurities. In all cases, ball-to-mass ratios were reasonable and the yield acceptable, as seen in Table 2.

**Table 2:** (2-Hydroxy)propylation of γ-CD of high DS.

No.	Molar fold of reagent	Milling time [h]	B2M ratio^a^	Product^b^ [g]	DS^c^	Yield^d^ [%]

7	10	8	8.5	2.0	8.8	56
8	20	8	10.9	2.9	17.6	63

^a^Ball-to-mass (mass of balls/mass of reagents); ^b^isolated, purified; ^c^calculated from the integration of anomeric-proton and CH_3_ signals of ^1^H NMR spectra and corrected with the residual-solvent content; ^d^on the base of DS.

Although hydroxypropylation was found to be very effective, unlike in the solution reactions, the HEBM method prefers water-free components, otherwise CD-hydrates form a hard solid, which sticks to the jar wall, during the salt preparation. While this is currently a considerable drawback for the mechanochemical method, the optimisation of the reagent ratios and milling parameters can offer a truly fine-tunable HPCD preparation method.

### 1-Chloro-2,3-epoxypropane ((±)-epichlorohydrin)

The potential of insoluble CDPs in various fields was recognised at a very early stage of CD derivatisation, and earlier than (2-hydroxy)propylation [15–16,22]. The use of epichlorohydrin can result in variously crosslinked polymers, depending on the molar ratio of the CD and epichlorohydrin [40–43]. As in the HPCD preparations, the aqueous basic solution can crosslink the macrocycles, while the relatively large OH^−^ excess can hydrolyse both the reagent and the simultaneously formed oxirane. In order to prepare bead CDPs, limited water miscibility solvents are added. Controlled addition to a homogeneous reaction mixture gives an alternative reaction product; soluble CD polymers. Unfortunately, the originally unfavourable CD/guest mass ratio worsens further, despite the good aqueous solubility of the CD polymers.

The molar ratio of CDs/epichlorohydrin was set to 1:10, which was based on the preparation of a soluble polymer [44]. Surprisingly, insoluble CDPs were almost exclusively formed at this molar ratio, unlike in the solution method. This result suggests that, in accordance with the practice, the soluble polymer contains a considerable amount of the glycidyl (2,3-dihydroxypropyl) sidechain in the solution reaction instead of the crosslinking ether units. It is known that the reaction can be directed to the glycidyl CD derivatives instead of polymerisation by varying the conditions [45–47].

Centrifugation of the neutralised suspension completely removed not only the inorganic salts but also all the soluble contaminants. No unsubstituted CDs were found in the solution phase and only a few charrable spots could be seen on the TLC plate. The slightly TLC running CD-dimers were also found to be soluble and were removed from the product with the washing, and the almost complete conversion of both CDs and epichlorohydrin could be deduced from the mass of dried products in all cases, as seen in Table 3. The supernatants were always hazy suggesting the presence of submicron particles, but their amounts were considered negligible.

**Table 3:** Synthesis of CDPs.

No.	Used CD	Total milling time [h]	B2M ratio^a^	Product^b^ [g]	Soluble part^c^ [g]	Yield^d^ [%]

9	β	6	8.8	3.3	<0.1	96
10^e^	β	9	4.5	32.4	1.4	88
11	β-hydrate	6	8.2	3.19	<0.1	91
12	γ	6	8.3	3.4	0.1	92
13	γ-hydrate	6	7.9	3.4	0.1	92
14^f^	β	9	11.7->8.8	0.9	1.3	–
15^g^	β	5	12.8	<0.1	2.2	75
16^h^	β	5	11.2	0.1	2.4^i^	76
17^h^	γ	5	10.3	0.2	2.5^i^	73

^a^Ball-to-mass (mass of balls/mass of reagents); ^b^isolated, purified; ^c^calculated from the freeze-dried washing-solution theoretical NaCl content; ^d^assuming that all epichlorohydrin was used for crosslinking; ^e^scale-up, used 125 mL jar; ^f^epichlorohydrin was added in 3 portions: at the beginning, after 3 h milling and after 6 h milling; ^g^3.3 molar-fold epichlorohydrin; ^h^5 molar-fold epichlorohydrin; ^i^not necessarily soluble but not sedimented and cannot be centrifuged.

Salt formation in the 10-fold scaled-up synthesis of the β-CD polymer resulted in some materials that became stuck on the jar wall. However, these were considerably easier to remove than the salts of hydrated CDs. The scale-up experiment (20 mmol scale) did not give a significantly lower yield. This lower yield may be due to the larger particles of the larger-scale experiments at the experiment start.

Attempts to prepare soluble CDPs provide a complex picture. While the ≈3 molar-fold crosslinker resulted in a clear solution, the 5 molar-fold reactions gave a colloid solution and actually it is not clear that it is a very fine, inseparable by centrifugation suspension, or a highly associated soluble polymer. When 10 molar-fold epichlorohydrin was added portionwise, both insoluble and soluble/solubilised polymers seemed to be formed. The soluble part was similar to the 5 molar-fold experiments; a very hazy solution was obtained, which was inseparable by centrifugation, after dialysis. Filtration through a 0.22 µm hydrophilic membrane showed poor resistance, meaning that particle sizes were low and dominantly <0.2 µm. Experiments to clarify the situation and determine the composition of the formed polymers are in progress.

**Figure 2:**
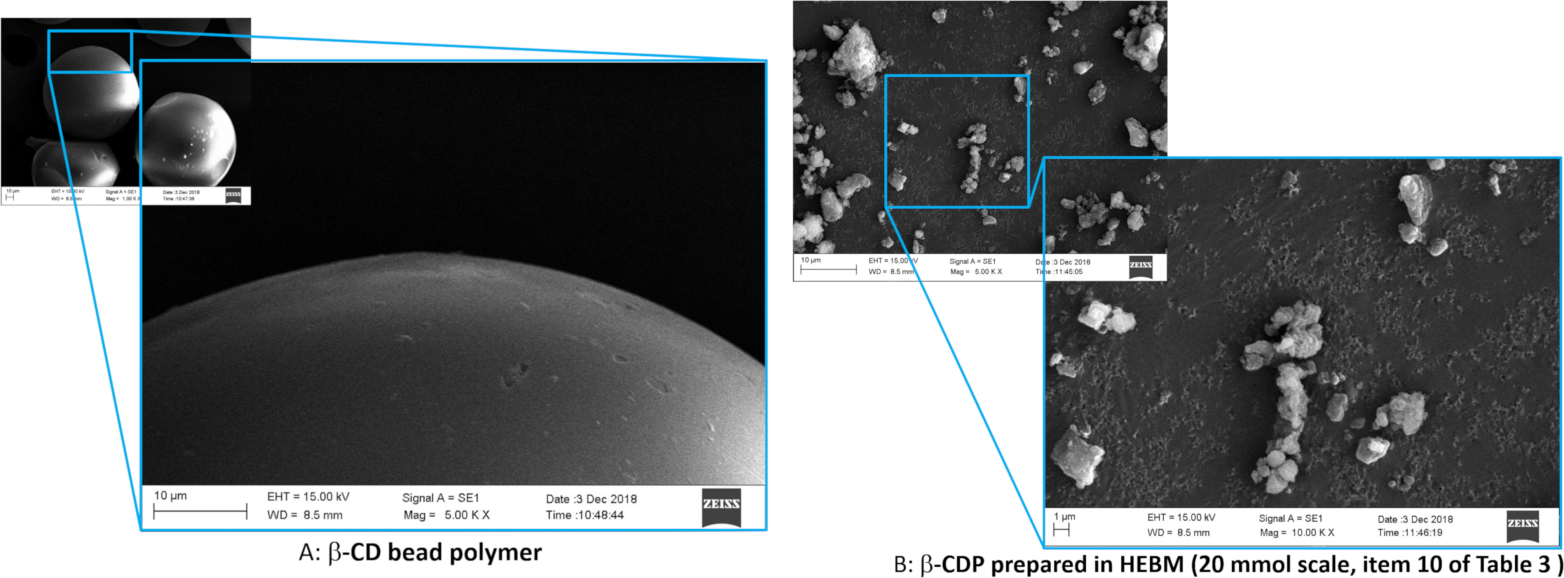
Comparative SEM pictures of a β-CD bead and β-CDP (20 mmol, Table 3, entry 10).

**Figure 3:**
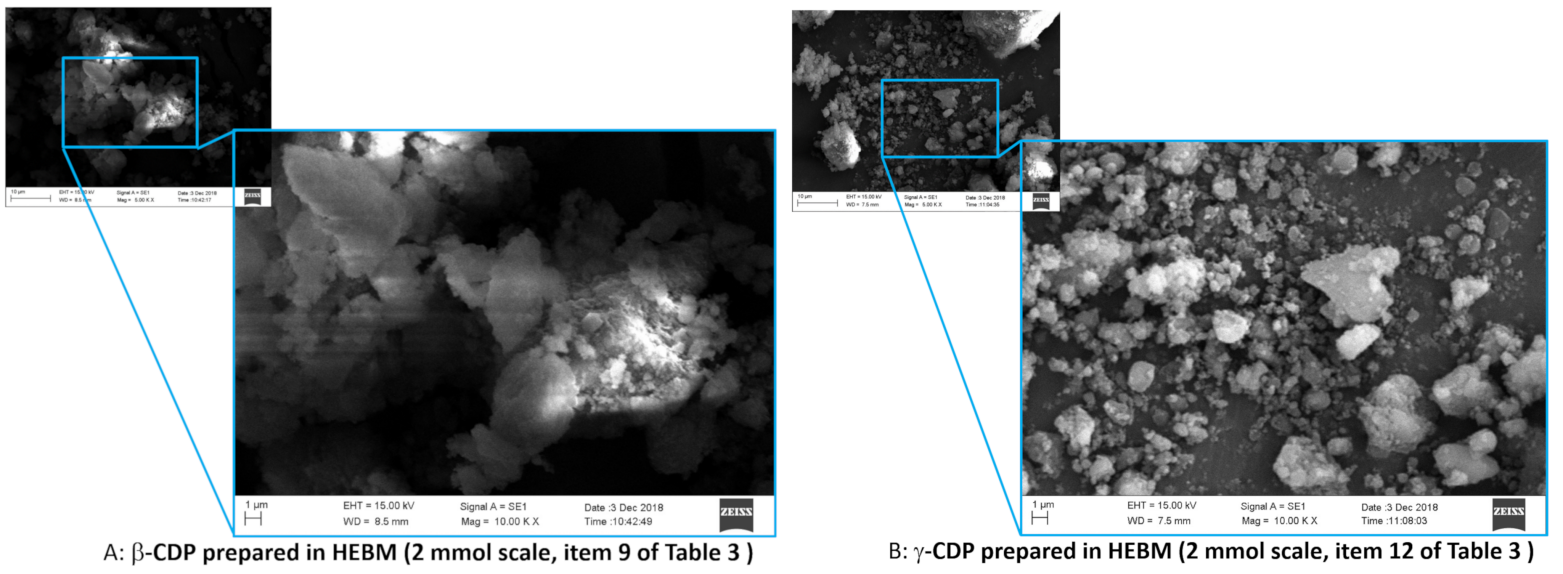
Comparison of β-CDP (Table 3, entry 9) and γ-CDP (Table 3, entry 12) prepared in a ball mill on 2 mmol scale.

Under an electron microscope, the bead polymer showed a smooth surface and a non-Gaussian particle-size distribution that was centred around 65–70 µm, while the ball-milled CDPs were completely bumpy and considerably smaller than the bead polymer, as seen in Figure 2 and Figure 3. Higher aggregation of particles of β-CDP was found when it had been prepared on a 2 mmol scale as compared to either the γ-CDP or β-CDP on a 20 mmol scale.

**Figure 4:**
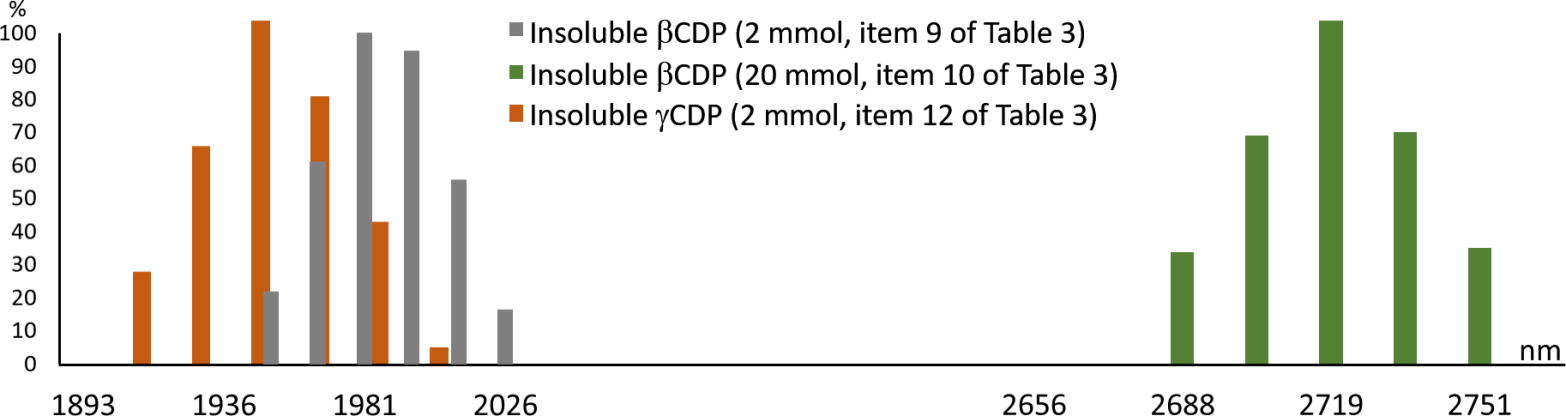
Normalised particle-size distribution of insoluble CD polymers (entries 9, 10, and 12 of Table 3).

The particle-size distribution, which was determined using the quasi elastic light scattering (QELS) method, was relatively tight and the 20 mmol scale reaction resulted in considerably larger particles, as seen in the normalised size distributions in Figure 4. However, it is necessary to mention that the cracking of the dried polymers of different scales were conducted under different conditions and that the larger particles can be assigned to the larger balls of the scaled-up product. The β-CDPs have a Gaussian distribution, while the γ-CDP does not. While it is interesting to note that γ-CDP gave smaller particles, although not significantly, it may also be true that the small β-CDP particles are more aggregated, as seen in Figure 2 and Figure 3.

**Figure 5:**
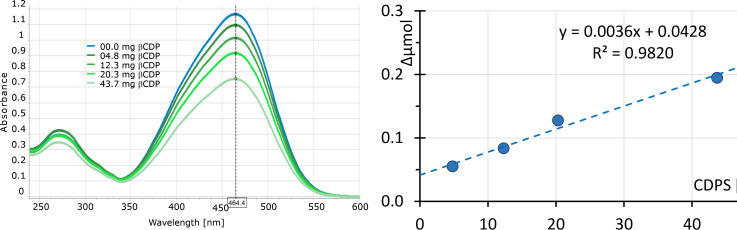
UV–vis spectra and adsorption isotherm of the insoluble β-CDP polymer in 10 ml 0.050 mM MO solution (entry 10 of Table 3) after 1 day of stirring.

Although scaled-up β-CDP has somehow larger particles, complexation studies with methyl orange (MO) only showed small differences. Although complex formation with MO showed similar behaviour to previous reports [13], adsorption capacity seems to be considerably lower than that of bead polymers in all cases. The adsorption of MO shows apparent first order kinetics, and the largest adsorption rates were accordingly found at the beginning of adsorption. The adsorption isotherm of insoluble β-CDP was recorded after 1 day and 2 weeks of equilibration. The adsorption isotherm of β-CDP showed linear concentration dependency, as seen in Figure 5.

It is known that, although γ-CD is able to form complexes with MO (they are less stable than those of β-CD), the prepared insoluble γ-CDP has few affinity towards MO. It can be also assumed that it is only the association rate that is lower than in the monomer. Particularly interesting results were observed when the same property of γ-CDP was compared to the corresponding (3-glycidyloxy)propylsilane γ-CD composite (next section). The complexation efficacy of the prepared CDPs is summarised in Table 4. No significant differences were found between the products that were prepared from the dried and hydrated CDs.

**Table 4:** Complexation efficacy of ≈20 mg insoluble CDPs in 0.050 mM MO solution after 1 day and 2 weeks (entry numbers are identical as those in Table 3.)

No.	CD used in CDP synthesis	Milling time [h]	Particle size^a^ [µm]	MO binding capacity [µmol/mg]		Relative MO binding capacity^b^

				1 day	14 days	

9	β	6	≈1.9	0.004	0.006	0.33
10^e^	β	9	≈2.9	0.005	0.009	0.51
11	β*H_2_O	6	≈1.7	0.007	0.010	0.61
12	γ	6	≈1.8	0.003	0.004	0.25
13	γ*H_2_O	6	≈2.2	<0.001	0.002	0.10
14	bead polymer^c^	N/A	≈77^d^	0.011	0.017	1.00

^a^By QELS; ^b^bead polymer = 1, calculated with not rounded values; ^c^Cyclolab’s CYL-2011; ^d^by electron microscope.

### (3-Glycidyloxypropyl)trimethoxysilane (GPTS)

**Figure 6:**
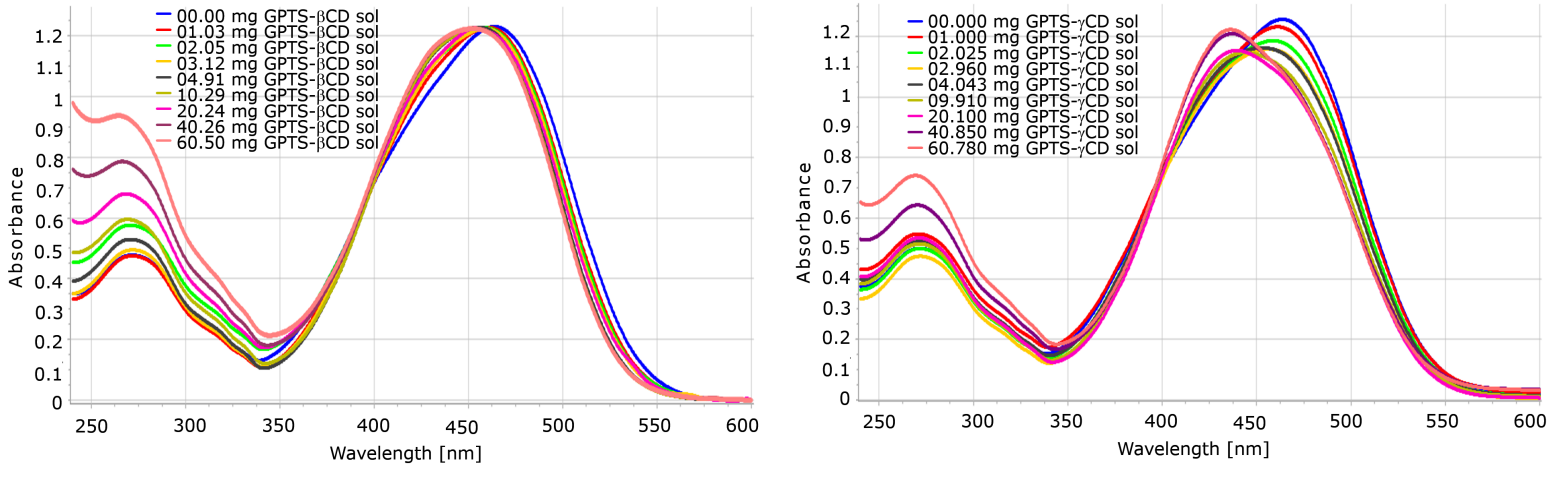
UV–vis spectral changes of 0.050 mM MO solution by GPTS-β-CD (left) and GPTS-γ-CD (right), as prepared in solution (entries 15 and 17 of Table 5).

**Figure 7:**
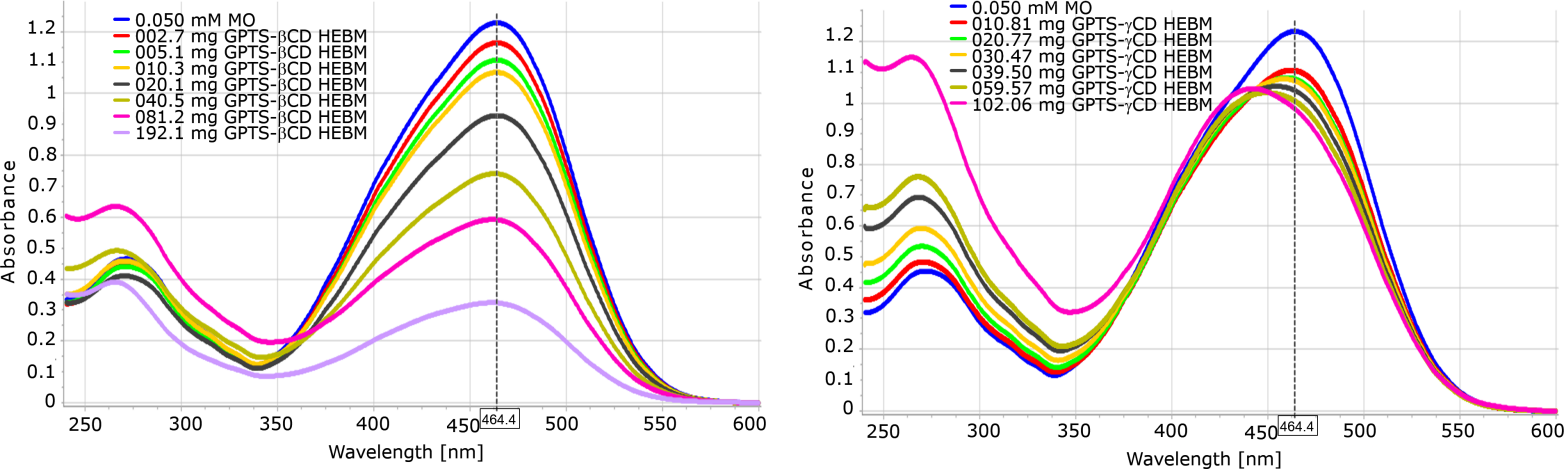
UV–vis spectral changes of 0.050 mM MO solution by GPTS-β-CD (left) and GPTS-γ-CD (right), as prepared in ball mill (entries 16 and 18 of Table 5).

The hydrolysis of GPTS occurs at two sites; on the oxirane ring and on the silyl ether moiety. The cleavage of trimethoxysilyl ether is unavoidable in the aqueous phase under basic conditions, while the oxirane ring seems to be more stable than in propylene oxide or epichlorohydrin. This is partially the consequence of its considerably lower aqueous solubility. It was found that the substitution reaction does not proceed, or at least does so very slow at temperatures below 60 °C. The GPTS reagent in water was found to be present at around 60–70%, like in case of propylene oxide. Although the neutralised reaction mixture was homogeneous, the isolation and NMR analysis of the isolated product showed poor methoxy content, demonstrating the almost complete hydrolysis of the silyl ethers. Unlike in the solution reaction, the HEBM isolated reaction products of β-CD were almost completely insoluble in water, while the γ-CD derivative was somehow soluble or solubilised, as seen in the UV–vis spectra. Both the β- and γ-CD derivatives showed high complexation affinity toward MO, as seen in the UV spectra in Figure 6 and Figure 7. However, unlike the epichlorohydrin polymers, their “concentration-dependency” was not linear. The solution and solid state reactions showed similar yields, as can be concluded from Table 5.

**Table 5:** Summary of GPTS-derivatisation of β- and γ-CD.

No.	Used CD	Reaction time [h]	B2M ratio	Product^c^ [g]	Yield^d^ [%]

15	β	48^a^	N/A	1.3	57
16	β	3^b^	10	4.2	76
17	γ	48^a^	N/A	2.2	88
18	γ	3^b^	10	5.3	88

^a^Solution reaction, at 70–75 °C; ^b^milling time; ^c^isolated purified compounds; ^d^on the base of DS, as determined by ^1^H NMR, in cases of solution reactions and in HEBM reactions, assuming that all GPTS is attached to the CDs.

A significant difference was found between GPTS-β-CD and -γ-CD that were prepared under HEBM conditions; a considerable part of the GPTS-γ-CD composite was solubilised. Not only were there no residual unsubstituted CDs seen on TLC of the prepared GPTS-CDs, but no running charrable spots were found either. GPTS, or its hydrolytic product(s), were run with the front, and they were found to be very poorly charrable. While it is not clear whether the product itself was dissolved or whether the particle size was smaller than <0.22 µm, the UV–vis spectra clearly show the hypsochromic shift that was caused by complexation with increasing amounts of added GPTS-γ-CD. The adsorption isotherm can also be divided in two parts; the simple adsorptive removal of MO can be seen at the beginning, while the MO peak showed a noticeable blue-shift as the amount of solubilised/dissolved γ-CD-derivative increased.

## Conclusion

The use of the HEBM method to force the reaction between epoxides and CDs was successful. It was also found that reagent utilization was higher than in solution reactions and that oxirane hydrolysis in the presence of a strong base, NaOH, could be hampered under ball-milling conditions.

Although the work-up and purification of the HEBM-reaction-synthesised CD derivatives was simpler in the (2-hydroxy)propylated cases, it does not seem possible to alter the current industrial method to make it more green.

Preparations of water-insoluble CD derivatives were more effective than the solution reactions, but the particle size of the solid state reaction products were very small, which also affected the complexation properties. Further studies to compare the physicochemical and complexation properties of the insoluble CD polymers are necessary and may be able to develop a feasible and reproducible alternative synthetic process. This would lead to the wider dissemination of insoluble epichlorohydrin-crosslinked CD polymers. The post-manipulation of fine CDP powder, such as granulation on a hard support, can improve the physicochemical and complexation properties of the polymers. Although the complexation properties of the prepared insoluble CD polymers are far from those of solution-method β-CD bead polymers, the fine-tuning and optimisation of the reaction conditions can be improved to offer a truly green synthesis that can exploit the combination of CD complexation and the elimination of aqueous solubility. Further optimisation of the synthesis conditions and structure elucidation are both in progress and will aid the development of an, at least, kilolab-scale procedure for standardised quality products.

## Experimental

The details of the reactions are described in Supporting Information File 1.

All reagents and organic solvents were used without further purification, except the ion-exchangers. The ion-exchangers were freshly washed with water and methanol until the washing solutions became colourless and UV inactive.

Reactions were carried out in a planetary ball mill (Retsch PM100 High Speed Planetary Ball Mill), using a 50 mL stainless steel jar and mix of stainless-steel balls (*m* = 44.1 g, in which ø = 5 mm, *m* = 28.1 g and 550 ø = 1–1.2 mm, *m* = 16.0 g) at 650 rpm for various time periods. The rotation direction was changed every 15 min (3 min during CD-Na salt preparation) with 3 seconds of silent periods between the alternating rotations. The scale-up of the insoluble CDP was done in a 125 mL stainless steel jar with a mixture of larger balls (*m* = 236.2 g, in which 7 ø = 12–13 mm, *m* = 97.1 g and 70 ø = 7 mm, *m* = 145.1 g) at 650 rpm. The same balls were used for 2 min at 450 rpm to crack the CDP solids after purification.

In the HEBM experiments the CD-sodium salts were cooled below −30 °C with liquid nitrogen before the addition of the reagents.

## Supporting Information

File 1 Experimental details, and the NMR and MS spectra of the soluble products.
