# Supplementary material for: Reaction of oxiranes with cyclodextrins under high-energy ball-milling conditions
Source: Beilstein J Org Chem. 2019 Jul 1;15:1448–59. doi: 10.3762/bjoc.15.145 (PMC6632222; doi:10.3762/bjoc.15.145)
Supplement: File 1 — Experimental details, and the NMR and MS spectra of the soluble products. [file Beilstein_J_Org_Chem-15-1448-s001.pdf]

BEILSTEIN JOURNAL OF ORGANIC CHEMISTRY

Supporting Information

for

Reaction of oxiranes with cyclodextrins under high-energy ball-milling conditions

László Jicsinszky, Federica Calsolaro, Katia Martina, Fabio Bucciol, Maela Manzoli and Giancarlo Cravotto

Beilstein J. Org. Chem. **2019,** *15,* 1448–1459. doi:10.3762/bjoc.15.145

Experimental details, and the NMR and MS spectra of the soluble products

License and Terms: This is a supporting information file under the terms of the Creative Commons Attribution License (http://creativecommons.org/licenses/by/4.0). Please note that the reuse, redistribution and reproduction in particular requires that the authors and source are credited.
The license is subject to the *Beilstein Journal of Organic Chemistry* terms and conditions: (https://www.beilstein-journals.org/bjoc)

Table of Contents

	Experimental scheme	S3
	Experimental details	S3
	Reagents	S3
		S3
	Synthesis	S5
	Synthesis of (2-hydroxy)propyl CDs	S5
Table S1	Summary of HPCD syntheses in HEBM.	S7
	Synthesis of CDPs	S8
Table S2	Summary of CD polymer syntheses in HEBM.	S9
	Reaction of (3-glycidyloxypropyl)trimethoxysilane (GPTS) and CDs	S10
Table S3	Summary of GPTS-derivatization of β- and γ-CD.	S11
	Adsorption experiments	S12

NMR spectra

Figure S1	^{1}H NMR of HP-β-CD, prepared in solution, DS ≈ 4.4	S13
Figure S2	^{1}H NMR of HP-β-CD, prepared in ball mill, DS ≈ 4.4, entry 1 of Table S1	S13
Figure S3	^{1}H NMR of HP-β-CD, prepared in ball mill, DS ≈ 5.6, entry 2 of Table S1	S14
Figure S4	^{1}H NMR of HP-β-CD, prepared in ball mill, DS ≈ 5.3, entry 3 of Table S1	S14
Figure S5	^{1}H NMR of HP-β-CD, prepared in ball mill, DS ≈ 3.7, entry 4 of Table S1	S15
Figure S6	^{1}H NMR of HP-γ-CD, prepared in solution, DS ≈ 4.5	S15
Figure S7	^{1}H NMR of HP-γ-CD, prepared in ball mill, DS ≈ 5.1, entry 5 of Table S1	S16
Figure S8	^{1}H NMR of HP-γ-CD, prepared in ball mill, DS ≈ 4.3, entry 6 of Table S1	S16
Figure S9	^{1}H NMR of HP-γ-CD, prepared in solution, DS ≈ 8.8, entry 7 of Table S1	S17
Figure S10	^{1}H NMR of HP-γ-CD, prepared in solution, DS ≈ 17.6, entry 8 of Table S1	S17
Figure S11	^{1}H NMR of GPTS-β-CD, prepared in solution, DS ≈ 2.3-2.6, entry 15 of Table S1	S18
Figure S12	^{1}H NMR of GPTS-γ-CD, prepared in solution, DS ≈ 2.5, entry 17 of Table S1	S18
Figure S13	^{13}C NMR of HP-β-CD, prepared in solution, DS ≈ 4.4	
Figure S14	^{13}C NMR of HP-β-CD, prepared in ball mill, DS ≈ 4.4, entry 1 of Table S1	S19
Figure S15	^{13}C NMR of HP-β-CD, prepared in ball mill, DS ≈ 5.6, entry 2 of Table S1	S20
Figure S16	^{13}C NMR of HP-β-CD, prepared in ball mill, DS ≈ 5.3, entry 3 of Table S1	S20
Figure S17	^{13}C NMR of HP-β-CD, prepared in ball mill, DS ≈ 3.7, entry 4 of Table S1	S21
Figure S18	^{13}C NMR of HP-γ-CD, prepared in solution, DS ≈ 4.5	S22
Figure S19	^{13}C NMR of HP-γ-CD, prepared in ball mill, DS ≈ 5.1, entry 5 of Table S1	S22
Figure S20	^{13}C NMR of HP-γ-CD, prepared in ball mill, DS ≈ 4.3, entry 6 of Table S1	S23
Figure S21	^{13}C NMR of HP-γ-CD, prepared in solution, DS ≈ 8.8, entry 7 of Table S1	S23
Figure S22	^{13}C NMR of HP-γ-CD, prepared in solution, DS ≈ 17.6, entry 8 of Table S1	S24
Figure S23	^{13}C NMR of GPTS-β-CD, prepared in solution, DS ≈ 2.3-2.6, entry 15 of Table S1	S25
Figure S24	^{13}C NMR of GPTS-γ-CD, prepared in solution, DS ≈ 2.5, entry 17 of Table S1	S25

MS spectra

Figure S25	ESIMS+ spectrum of HP-β-CD, prepared in solution, DS ≈ 4.4	S26
Figure S26	ESIMS+ spectrum of HP-β-CD, prepared in ball mill, DS ≈ 4.4, entry 1 of Table S1	S27
Figure S27	ESIMS+ spectrum of HP-β-CD, prepared in ball mill, DS ≈ 5.6, entry 2 of Table S1	S28
Figure S28	ESIMS+ spectrum of HP-β-CD, prepared in ball mill, DS ≈ 5.3, entry 3 of Table S1	S29
Figure S29	ESIMS+ spectrum of HP-β-CD, prepared in ball mill, DS ≈ 3.7, entry 4 of Table S1	S30
Figure S30	ESIMS+ spectrum of HP-γ-CD, prepared in solution, DS ≈ 4.5	S31
Figure S31	ESIMS+ spectrum of HP-γ-CD, prepared in ball mill, DS ≈ 5.1, entry 5 of Table S1	S32
Figure S32	ESIMS+ spectrum of HP-γ-CD, prepared in ball mill, DS ≈ 2.3-2.6, entry 6 of Table S1	S33
Figure S33	ESIMS+ spectrum of HP-γ-CD, prepared in solution, DS ≈ 8.8, entry 7 of Table S1	S34
Figure S34	ESIMS+ spectrum of HP-γ-CD, prepared in solution, DS ≈ 17.6, entry 8 of Table S1	S36
Figure S35	ESIMS+ spectrum of GPTS-β-CD, prepared in solution, DS ≈ 2.3-2.6, entry 15 of Table S1	S37
Figure S36	ESIMS+ spectrum of GPTS-γ-CD, prepared in solution, DS ≈ 2.3-2.6, entry 17 of Table S1	S38
Figure S37	ESIMS+ spectrum of background (10 mM NaCl)	S39

UV spectra

Figure S38	UV–vis spectra of CDPs* after 1 day equilibration of ≈ 20 mg CDP and 0.05 mM methyl orange solution	S40
Figure S39	UV–vis spectra of CDPs* after 2 weeks equilibration of ≈ 20 mg CDP and 0.05 mM methyl orange solution	S40
Figure S40	Soluble GPTS-βCD (entry 15* Table 5) caused blueshifts in 0.05 mM methyl orange solution	S41
Figure S41	Soluble GPTS-γ-CD (entry 16* Table 5) caused blueshifts in 0.05 mM methyl orange solution	S41

Low resolution SEM pictures of CDPs

Figure S42	100 μm resolution SEM picture of insoluble β-CDP (entry 9* of Table 5)	S42
Figure S43	100 μm resolution SEM picture of insoluble β-CDP (entry 10* of Table 5)	S42
Figure S44	100 μm resolution SEM picture of insoluble γ-CDP (entry 12* of Table 5)	S43
Figure S45	200 and 100 μm resolution SEM picture of insoluble β-CDP bead (entry 14* of Table 5)	S43

Particle size analysis

Figure S46	Particle size analysis of insoluble β-CDP (entry 9* Table 5)	S44
Figure S47	Particle size analysis of insoluble β-CDP (entry 10* Table 5)	S44
Figure S48	Particle size analysis of insoluble β-CDP (entry 11* Table 5)	S45
Figure S49	Particle size analysis of insoluble γ-CDP (entry 12* Table 5)	S45
Figure S50	Particle size analysis of insoluble γ-CDP (entry 13* Table 5)	S46

*Entry labels are corresponding to the body text, not the SI.

Experimental scheme

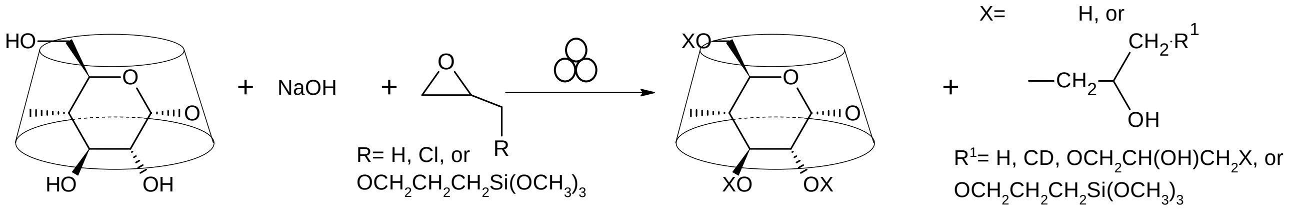

Scheme S1: The reaction of CDs with oxiranes.

Experimental details

Reagents

All reagents and organic solvents were used without further purification, except the ion-exchangers. β- and γ-CD hydrates were obtained from Wacker Chemie AG Div. Biosolutions, Germany, and when the complete removal of crystalline water was necessary, they were dried at 80–100 °C in the presence of KOH and P_2O_5. β-CD bead polymer (CYL-2011) is from Cyclolab Ltd., Budapest, Hungary. Organic solvents, (*R*/*S*)-1,2-epoxypropane (99.5%), (±)-epichlorohydrin (99.9%), (3-glycidyloxypropyl)trimethoxy-silane (98%), NaOH pellet (>97%), activated strong ion-exchangers (Amberlite IRA-402(OH) and Amberlite® IR-120(H)) were purchased from Sigma-Aldrich. TLC (Merck 5554 UV_{F254}) plates were obtained from Merck KGaA, Darmstadt, Germany). Because the reagents were used in excess, the amounts used were not corrected in relation to their contents, except the water content of the CDs. The ion-exchangers were freshly washed with water and methanol until the washing solutions became colourless and UV inactive. When neutralisation was performed in the cation-exchanger, a small amount (≈5%) of anion-exchanger was added to remove the corresponding degradation products of the ion-exchanger polymers.

Instruments and conditions

Reactions were carried out in a planetary ball mill (Retsch PM100 High Speed Planetary Ball Mill), using a 50 ml stainless steel jar and a stainless-steel ball mix (m = 44.1 g, in which ø= 5 mm, m = 28.1 g and 550 ø= 1–1.2 mm, m = 16.0 g) at 650 rpm for various time periods. The rotation direction was changed every 15 min (3 min during CD-Na salt

preparation) with 3 seconds of silent periods between the alternating rotations. The scale-up of the insoluble CDP was done in a 125 ml stainless steel jar with a mixture of larger balls (*m* = 236.2 g, in which 7 ø = 12–13 mm, *m* = 97.1 g and 70 ø = 7 mm, *m* = 145.1 g) at 650 rpm. The same balls were used for 2 min at 450 rpm to crack the CDP solids after purification and drying. The CD-sodium salts were cooled below −30 °C before the additions of the reagents with liquid nitrogen.

Centrifugation was performed at 4000 rpm for 30–60 min in 50 ml plastic centrifuge tubes.

For dialysation, a Slide-A-Lyzer 2k G2 (Thermofisher Scientific, Waltham, MA, USA) cassette was used in slowly stirred distilled water (500 ml).

Absence of chloride was checked by 0.1 M $AgNO_3$ solution after suspending the CDPs in 5% HNO_3 solution.

ESIMS experiments used 1 mM NaCl solutions and spectra were recorded in Waters Micromass ZQ equipment. Mass assignment was done with the help of mMass v5.5 software (Open Source Mass Spectrometry Tool, http://www.mmass.org).

NMR spectra were recorded in a Bruker Avance 300 MHz and JEOL 600 MHz. Evaluations used the ACD/NMR Processor Academic Edition Release 12.00 product version 12.01 build 39104, (Advanced Chemistry Development Inc., Toronto, Ontario, Canada).

UV–vis spectra were recorded in an Agilent UV Cary60 spectrometer. They were evaluated and figures were created using Spectragryph v1.2.10 software (Spectragryph Software for optical spectroscopy v1.2.10, Dr. Friedrich Menges, Obersdorf, Germany, https://www.effemm2.de).

Size-distribution experiments were performed in a Brookhaven 90Plus Particle Size Analyzer using the Quasi Elastic Light Scattering (QELS) method (Brookhaven Instruments Corporation, Holtsville, NY, USA).

SEM pictures were recorded in Zeiss equipment at 15 kV using samples adsorbed on gold metal.

TLC used a 7 cm running distance in a saturated chamber of 10:7 (v/v) 1,4-dioxane-conc. aqueous ammonia. Samples of solid materials were dissolved (or suspended in the case of insoluble polymers) to 2 % in water and spotted in 10 µL using a microsyringe (25 µL, Hamilton Bonaduz AG, Switzerland) and a dry N_2-stream, which allowed semiquantitative evaluations to be performed and confirmed the unsubstituted CD-contents below or close to the detection level (<0.2 µg, ≈0.1%).

The temperature of the jar was monitored periodically with an RS-8662 IR thermometer (RS Components Ltd., Corby, UK).

Membrane filtrations used 0.22 µm hydrophilic membrane (Unichro® PTFE/B, Cobetter Italy Srl, Agrate Brianza, Italy).

Drying of CDs were conducted at 80–90 °C under reduced pressure in the presence of KOH and P_2O_5 until constant weight was obtained (1–2 days).

Synthesis

Synthesis of (2-hydroxy)propyl CDs

a) Solution.

CD hydrate (0.010 mol, 13.2 g β-CD- or 14.4 g γ-CD-hydrate) was dissolved in aqueous (25 ml) NaOH (1.6 g, 0.040 mol) and immersed in an ice bath for 30 min. Propylene oxide (2.9 g, 3.5 ml, 0.060 mol) was then added slowly but not dropwise. The reaction mixture was vigorously stirred for 4 days in the water bath, which formed as the ice melted. The reaction mixture was neutralised with the cation-exchanger (25 g) at rt with overnight stirring, then clarified with charcoal (0.8 g) at rt for 30 min, filtered, washed with water (4 × 15 ml) and the almost colourless solution was freeze-dried. The solid was dissolved in MeOH (15 ml) and acetone (150 ml) and was added slowly under the action of the lab-cleaning ultrasonicator. The solid was filtered off and washed with acetone (3 × 15 ml) and then dried at 60–70 °C for two days. The (oligo)PG content of the isolated solids were

below the detection limits of NMR and MS. Yields: (12.3 g, DS ≈ 4.4, 89% HP-β-CD; 13.6 g, DS ≈ 4.5, 87% HP-γ-CD)

b) HEBM

CDs (0.002 mol, freshly dried or the hydrate) and NaOH (0.16 g, 0.004 mol) were milled for 15 min (5 × 3 min alternating directions). The jar temperature increased to 39–41 °C and the milled solids warmed to 55–60 °C. When the salts of the hydrates were prepared, the solids stuck to the jar wall and were removed with a spatula before cooling. The closed jar was cooled below –30 °C (using liquid N_2) and then propylene oxide (0.58 g, 0.7 ml, 0.010 mol) was added. The mixture was then milled for a couple of hours. When the milling was over, the solid was dissolved in MeOH (15 ml), transferred to an Erlenmeyer flask, and the balls and jar were washed with MeOH (4 × 5 ml). The methanolic solution/suspension was treated with a cation-exchanger (2.5 g) and charcoal (0.2 g), for 4 h at rt, filtered, washed with MeOH (4 × 5 ml), the solution was then concentrated in a rotavapor to ≈ 4.5–5 g, and acetone (30 ml) was added under ultrasonication. The solids were filtered off and washed with acetone (3 × 5 ml). The drying of the solids at 60–70 °C in an oven for two days gave white solids (2.2–2.6 g). The removal of the solvents from the mother liquors afforded more or less hygroscopic (depending on the PG content) white solids (0.2–0.3 g).

The recovery of the adsorbed compounds from the ion-exchangers and carbon made use of 50% aq. MeOH (3 × 20 ml). They were analysed using TLC, and the residue was in the range of 0.15–0.30 g after the removal of the solvents. The solids were practically insoluble in solvents at a tenths-of-a-per-cent level in water, 1:1 water/MeOH or EtOH, and DMF. TLC showed dominant monoHP CD content in the desorbed solids.

High DS HP-γ-CDs were prepared using identical amounts of freshly dried γ-CD as above (2.6 g, 0.002 mol), NaOH (0.16 g, 0.004 mol), and 10- or 20-molar fold of propylene oxide (1.16 g, 1.4 ml or 2.32 g, 2.8 ml), but the reaction times were longer, 8 hours, as

suggested by previous experiences. Once the milling was finished, the products were dissolved in MeOH (10 ml), neutralized with conc. HCl (≈0.3 ml), transferred to the dialysation cassette, and washed with MeOH (4 × 1.5 ml), then dialyzed for 8 hours. The external water was changed with fresh water and the dialysis was repeated overnight. The dialysate was then treated with charcoal (0.5 g) at rt for 1 h, filtered, and washed with water (3 × 2 ml). Freeze-drying afforded an off-white, non-hydroscopic solid (2.0 and 2.9 g, respectively) with a DS values of 8.8 and 17.6, as determined by ^{1}H NMR, and the sharp peaks of PGs could not be seen. ESMS of the products showed small no signals in the range of mono-, di-, tri- and tetra-PGs.

Summary of syntheses

Table S1: *Summary of HPCD syntheses in HEBM.*

No.	CD/oxirane ratio	Milling time [h]	B2M ratio[a]	Product[b] [g]	DS[c]	Yield[d] [%]
1	β/5	2	14.6	2.3	4.4	84
2	β/5	4	14.6	2.3	5.6	78
3	β/5	3.5	14.6	2.5	5.3	87
4	β-hydrate/5	3.5	13.0	2.4	3.7	89
5	γ/5	2	13.2	2.7	5.1	84
6	γ-hydrate/5	3.5	12.2	2.4	5.5	73
7	γ/10	8	8.5	2.0	8.8	56
8	γ/20	8	10.9	2.9	17.6	63

[a] Ball-to-mass (mass of balls/mass of reagents); [b] isolated, purified; [c] calculated from the integration of the anomeric-proton and CH_3 signals of ^{1}H-NMR spectra and corrected using the residual solvent content; [d] on the base of DS.

Synthesis of CDPs

The HEBM reactions were carried out as above using 0.002 mol CDs (2.3 g β-CD or 2.6 g γ-CD on dry basis), 0.021 mol (0.84 g) NaOH and 0.020 mol (≈1.85 g, 1.6 ml) epichlorohydrin. Once the milling was over, the yellow solids were sieved, suspended in water (40 ml), neutralised with 1 N HCl (1.1–1.2 ml) and the solid was separated and washed with water (4 × 15 ml) with centrifugation. The resulting solid was dried for a day in a 60–70 °C oven. The obtained rocks were milled (450 rpm, 2 min) to give a fine powder. Drying at 60–70 °C for 2 days gave an off-white solid (3.2–3.5 g), which was free of soluble CD derivatives. The supernatants were combined and freeze-dried. TLC of the obtained solids showed minimal charrable spots near the start and no starting CD and only traces of chloride.

The 10 times scale-up experiment was carried out in a similar manner, using larger balls and identical molar and solvent ratios, and resulted in 32.5 g of product (≈88% based on assumed complete utilisation and no hydrolysis of epichlorohydrin).

Attempts to prepare soluble polymers were performed as in the insoluble CDPs experiments, but with lower molar ratio of NaOH and epichlorohydrin. After milling, the reaction mixture was suspended in 15 ml water. The jar and balls were washed with water (5 × 3 ml), neutralized with conc. HCl (20–80 µl) and the solids were removed with centrifugation. The inorganic salts were removed by dialysation as it is described for the HP-γ-CD of high DS.

Table S2: *Summary of CD polymer syntheses in HEBM.*

No.	Used CD	Total milling time [h]	B2M ratio[a]	Product[b] [g]	Soluble part[c] [g]	Yield[d] [%]
9	β	6	8.8	3.3	<0.1	96
10[e]	β	9	4.5	32.4	1.4	88
11	β-hydrate	6	8.2	3.19	<0.1	91
12	γ	6	8.3	3.4	0.1	92
13	γ-hydrate	6	7.9	3.4	0.1	92
14[f]	β	9	11.7->8.8	0.9	1.3	–
15[g]	β	5	12.8	<0.1	2.2	75
16[h]	β	5	11.2	0.1	2.4[i]	76
17[h]	γ	5	10.3	0.2	2.5[i]	73

[a] Ball-to-mass (mass of balls/mass of reagents); [b] isolated, purified; [c] calculated from the freeze-dried washing-solution theoretical NaCl content; [d] assuming that all epichlorohydrin was used for crosslinking; [e] scale-up, used 125 ml jar; [f] epichlorohydrin was added in 3 portions: at the beginning, after 3 h milling and after 6 h milling; [g] 3.3 molar-fold epichlorohydrin; [h] 5 molar-fold epichlorohydrin; [i] not necessarily soluble but not sedimented and cannot be centrifuged.

Reaction of (3-glycidyloxypropyl)trimethoxysilane (GPTS) and CDs

a) Solution

The reaction was carried out on a 1.5 mmol scale (2.0 g β-CD-hydrate, 2.2 g γ-CD-hydrate) using 4.5 mmol GPTS (1.1 g, 1 ml), as in the propylene oxide reactions. The reaction mixture was then stirred at 60 °C for 2 days. The reaction mixture was neutralised with the cation-exchanger, filtered, washed with water (4 × 15 ml), then clarified with charcoal (0.8 g) at rt for 30 min and the almost colourless solution was freeze-dried. The solid was dissolved in water (10 ml) and dialysed overnight. The freeze-drying of the dialysate gave a white, light, and electrostatic solid (GPTS-β-CD: 1.6 (≈65–70%) GPTS-γ-CD: 2.2 g (≈75–80%), yields are calculated for DS = 2.0 (β-CD) and 2.5 (γ-CD), respectively).

b) HEBM

The reactions were carried as above, but on a 0.003 mol scale (3.4 g β-CD or 3.9 g γ-CD on dry basis), using 0.009 mol NaOH (0.36 g) and 0.009 mol GPTS (2.13 g, 2.0 ml). Once the milling was finished, the solids were sieved, resulting in 5.4-6.5 g yellow, very light solids. The solid was suspended in MeOH (30 ml) and conc. HCl (50 µl) was added. The solids were then filtered off and washed to neutral with MeOH (5 × 10 ml), were found to be insoluble in water, then washed with water (3 × 10 ml). The filtration of the aqueous washing did not used vacuum until the half of the liquid had dropped otherwise the filtration took more than a day. The drying (60–70 °C, 2 days) gave pale yellow solids, which were periodically disintegrated with spatula (GPTS-β-CD: 4.2 g, GPTS-γ-CD: 4.0 g). They did not contain unsubstituted CDs or any charrable organic materials by TLC.

***Table S3**: Summary of GPTS-derivatization of β- and γ-CD.*

No.	Used CD	Reaction time [h]	B2M ratio	Product[c] [g]	Yield[d] [%]
15	β	48[a]	N/A	1.6	57
16	β	3[b]	10	4.2	77
17	γ	48[a]	N/A	2.2	88
18	γ	3[b]	10	4.0	67

[a] Solution reaction, at 70–75 °C; [b] milling time; [c] isolated purified compounds, in solution, 1.5 mmol CD, in HEBM 3 mmol CD; [d] on the base of DS, as determined by ^{1}H-NMR, in cases of solution reactions and in HEBM reactions, assuming that all GPTS is attached to the CDs and all $Si(OMe)_3$ was converted to $Si(OH)_3$.

Adsorption experiments

The CDP and GPTS-CD adsorption experiments used a 0.050 mM methyl orange solution (pH 7.2–7.5). The studied compounds were added in various amounts to the solution (10 ml), were stirred for 1 day at 25 ± 1 °C, and allowed to sediment overnight. They were then filtered through a 0.22 µm hydrophilic membrane. The UV–vis spectra of the clear solutions were recorded without further dilution.

Acquisition Time (sec)	2.9046	***Comment***	single_pulse	***Date***	23 Oct 2018 09:58:58		
Date Stamp	23 Oct 2018 09:58:25			***File Name***	E:\Documents\!!Notebooks\!BM_Reactions\NMR\HPxCD\HPXCD_LJ00bSolution_Proton-1-1.esp		
Frequency (MHz)	600.17	***Nucleus***	1H	***Number of Transients***	8	***Origin***	ECA
Original Points Count	32768	***Owner***	delta	***Points Count***	262144	***Pulse Sequence***	proton.jxp
Solvent	D2O	***Spectrum Offset (Hz)***	3901.5425	***Sweep Width (Hz)***	11281.59	***Temperature (degree C)***	21.100

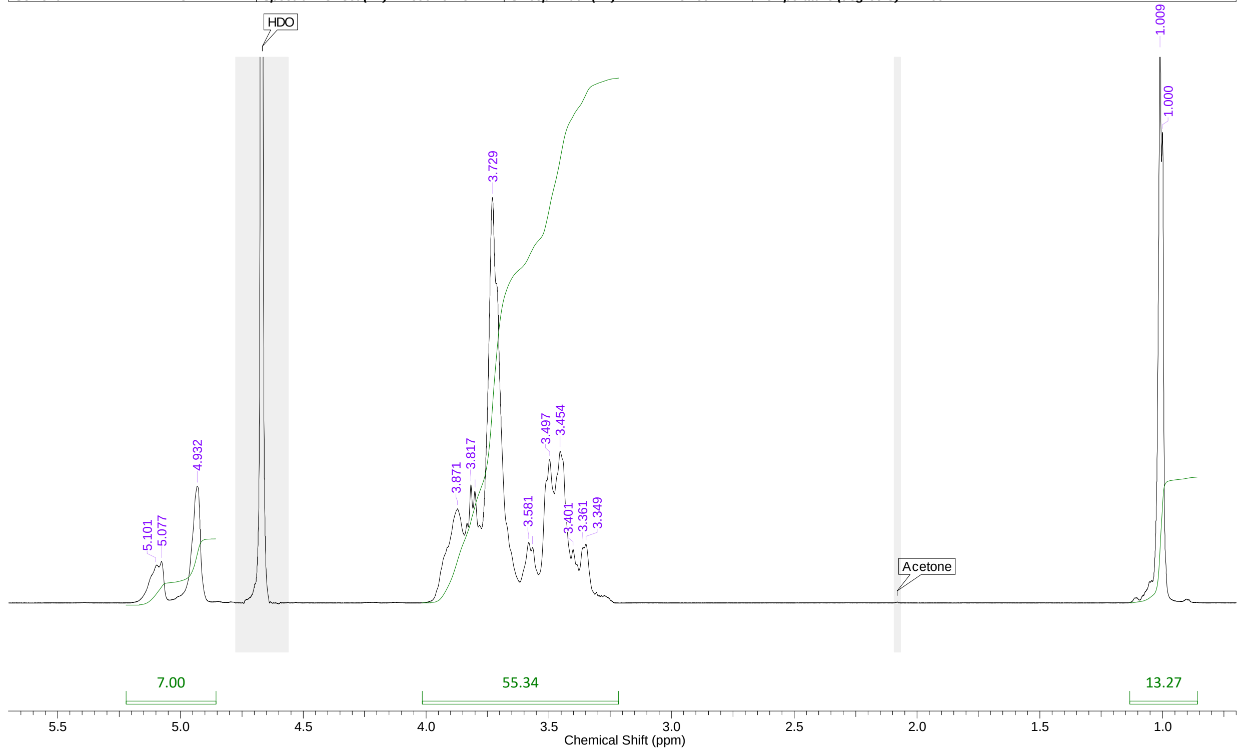

Figure S1: ^{1}H NMR of HP-β-CD, prepared in solution, DS ≈ 4.4

Acquisition Time (sec)	2.9046	***Comment***	single_pulse	***Date***	30 Oct 2018 11:28:13		
Date Stamp	30 Oct 2018 11:27:41			***File Name***	E:\Documents\!!Notebooks\!BM_Reactions\NMR\HPxCD\HPXCD_LJ01_Proton-1-1.esp		
Frequency (MHz)	600.17	***Nucleus***	1H	***Number of Transients***	8	***Origin***	ECA
Original Points Count	32768	***Owner***	delta	***Points Count***	131072	***Pulse Sequence***	proton.jxp
Solvent	D2O	***Spectrum Offset (Hz)***	3900.9656	***Sweep Width (Hz)***	11281.59	***Temperature (degree C)***	20.300

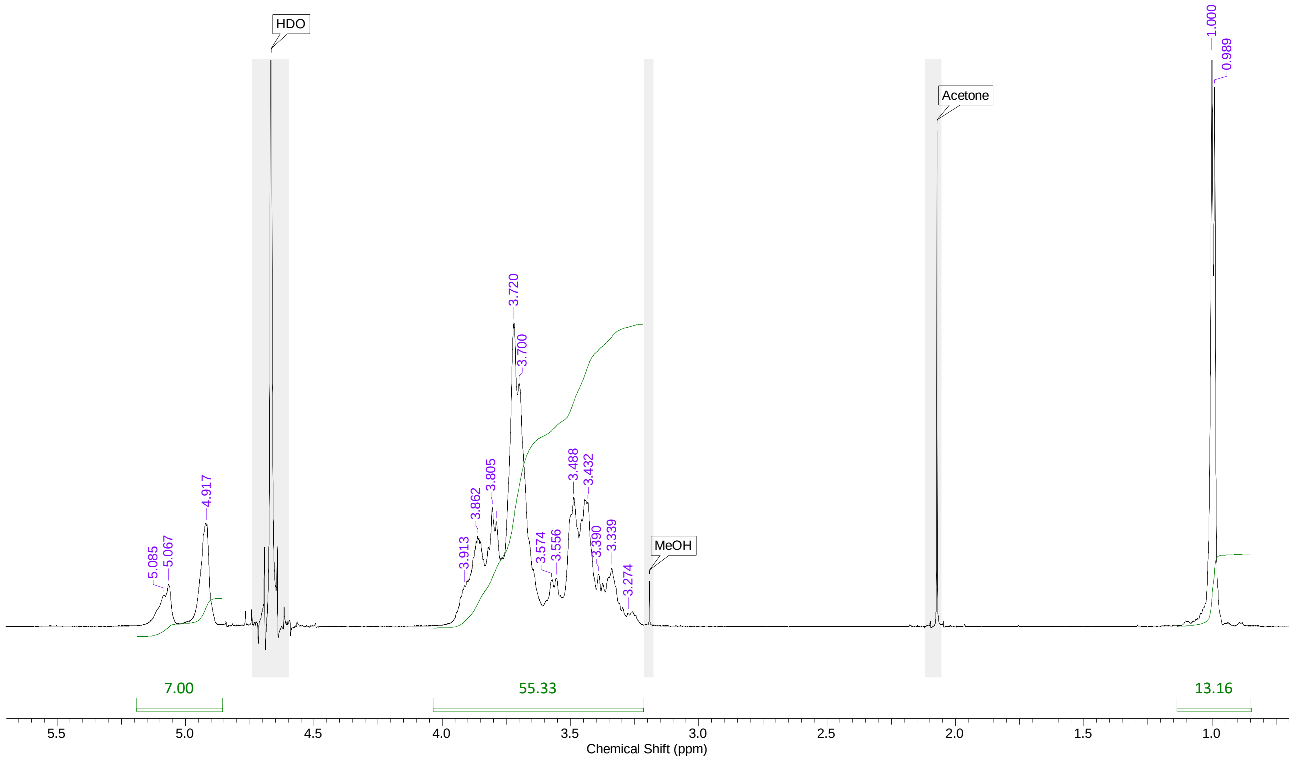

Figure S2: ^{1}H NMR of HP-β-CD, prepared in ball mill, DS ≈ 4.4, entry 1 of Table S1

Acquisition Time (sec)	2.9046	***Comment***	single_pulse	***Date***	26 Oct 2018 08:48:21		
Date Stamp	26 Oct 2018 08:47:49			***File Name***	E:\Documents\!!Notebooks\!BM_Reactions\NMR\HPxCD\HPxCD_LJ02_Proton-1-1.esp		
Frequency (MHz)	600.17	***Nucleus***	1H	***Number of Transients***	8	***Origin***	ECA
Original Points Count	32768	***Owner***	delta	***Points Count***	131072	***Pulse Sequence***	proton.jxp
Solvent	D2O	***Spectrum Offset (Hz)***	3901.2236	***Sweep Width (Hz)***	11281.59	***Temperature (degree C)***	21.300

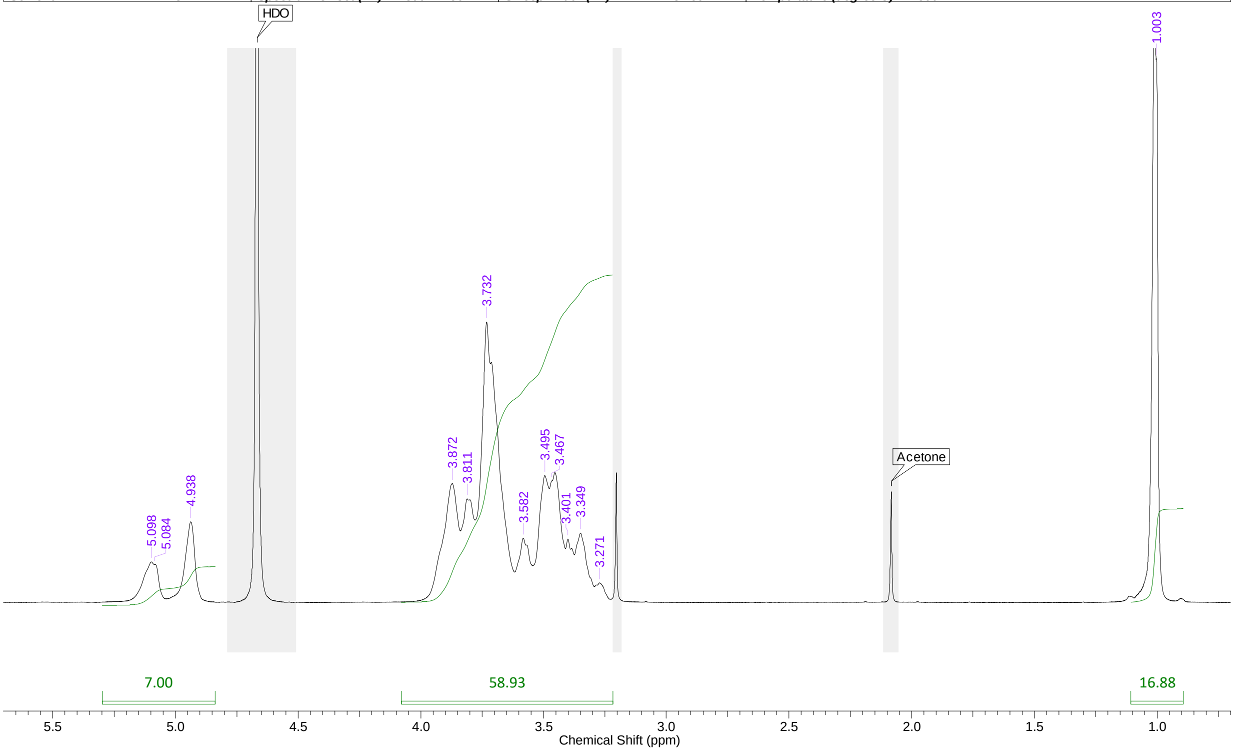

Figure S3: ^{1}H NMR of HP-β-CD, prepared in ball mill, DS ≈ 5.6, entry 2 of Table S1

Acquisition Time (sec)	2.9046	***Comment***	single_pulse	***Date***	26 Oct 2018 08:20:43		
Date Stamp	26 Oct 2018 08:20:10			***File Name***	E:\Documents\!!Notebooks\!BM_Reactions\NMR\HPxCD\HPxCD_LJ03_Proton-1-1.esp		
Frequency (MHz)	600.17	***Nucleus***	1H	***Number of Transients***	8	***Origin***	ECA
Original Points Count	32768	***Owner***	delta	***Points Count***	131072	***Pulse Sequence***	proton.jxp
Solvent	D2O	***Spectrum Offset (Hz)***	3901.2239	***Sweep Width (Hz)***	11281.59	***Temperature (degree C)***	21.200

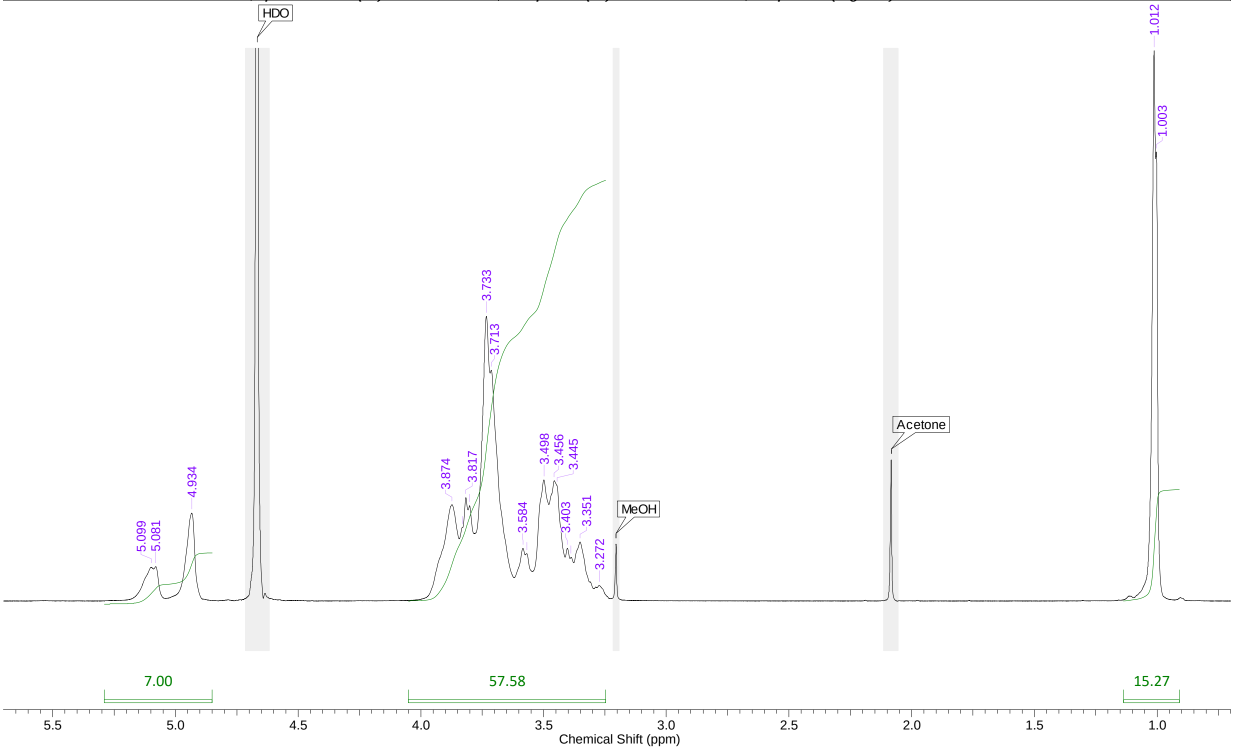

Figure S4: ^{1}H NMR of HP-β-CD, prepared in ball mill, DS ≈ 5.3, entry 3 of Table S1

Acquisition Time (sec)	2.9046	***Comment***	single_pulse	***Date***	30 Oct 2018 11:53:58		
Date Stamp	30 Oct 2018 11:53:25			***File Name***	E:\Documents\!!Notebooks\!BM_Reactions\NMR\HPxCD\4SI\proton\HPxCD_LJ05_Proton-1-1.es		
Frequency (MHz)	600.17	***Nucleus***	1H	***Number of Transients***	8	***Origin***	ECA
Original Points Count	32768	***Owner***	delta	***Points Count***	131072	***Pulse Sequence***	proton.jxp
Solvent	D2O	***Spectrum Offset (Hz)***	3901.0518	***Sweep Width (Hz)***	11281.59	***Temperature (degree C)***	20.300

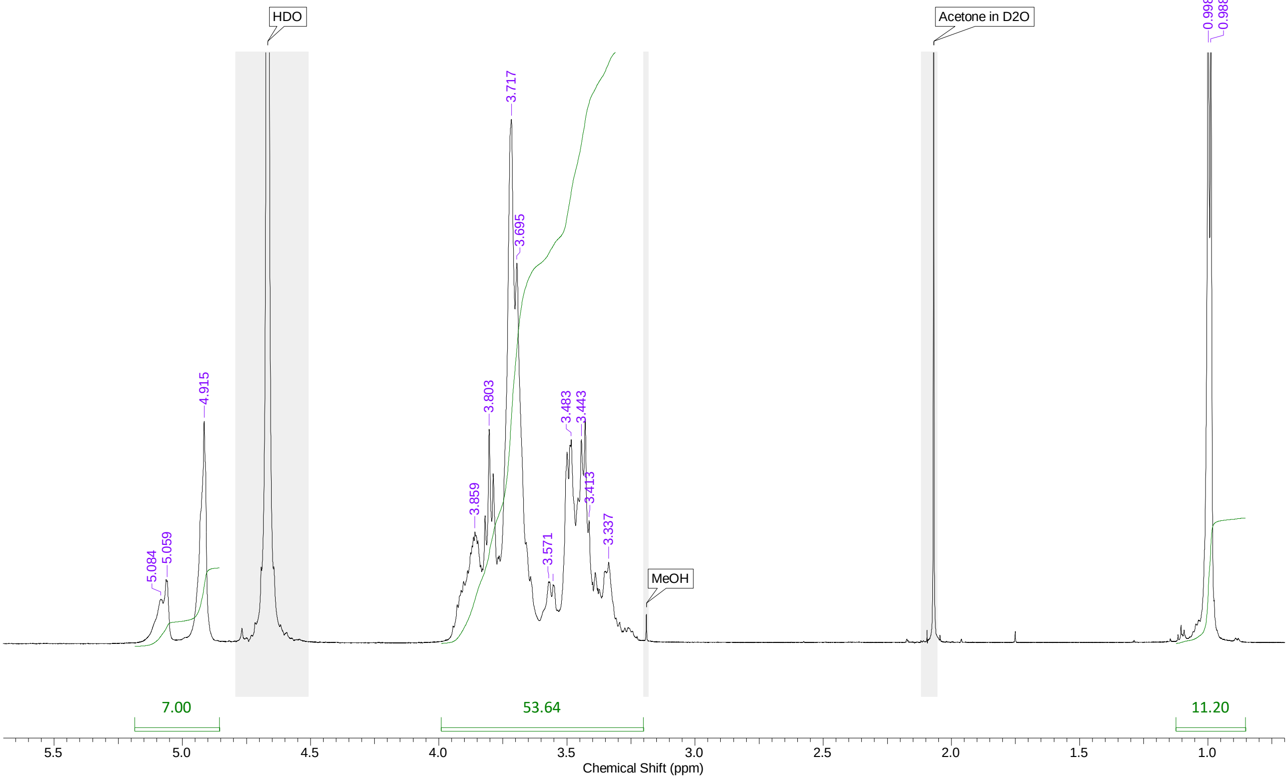

Figure S5: ^{1}H NMR of HP-β-CD, prepared in ball mill, DS ≈ 3.7, entry 4 of Table S1

Acquisition Time (sec)	2.9046	***Comment***	single_pulse	***Date***	17 Dec 2018 11:17:23		
Date Stamp	17 Dec 2018 11:16:50			***File Name***	E:\Documents\!!Notebooks\!BM_Reactions\NMR\HPxCD\HPxCD_LJ00gSolution_Proton-1-1.esp		
Frequency (MHz)	600.17	***Nucleus***	1H	***Number of Transients***	8	***Origin***	ECA
Original Points Count	32768	***Owner***	delta	***Points Count***	262144	***Pulse Sequence***	proton.jxp
Solvent	D2O	***Spectrum Offset (Hz)***	3902.1880	***Sweep Width (Hz)***	11281.59	***Temperature (degree C)***	18.200

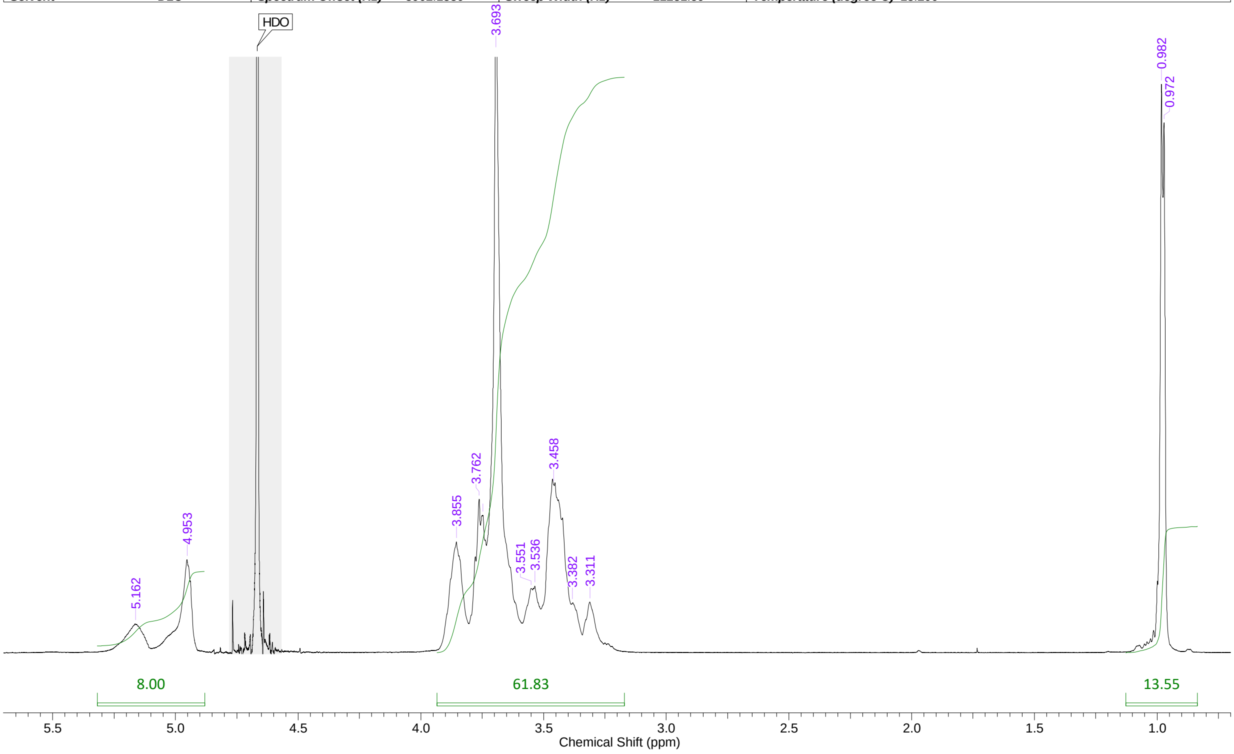

Figure S6: ^{1}H NMR of HP-γ-CD, prepared in solution, DS ≈ 4.5

**Acquisition Time (sec)**	2.9046	**Comment**	single_pulse	**Date**	30 Oct 2018 12:19:46		
**Date Stamp**	30 Oct 2018 12:19:13			**File Name**	E:\Documents\!!Notebooks\!BM_Reactions\NMR\HPxCD\HPXCD_LJ04_Proton-1-1.esp		
**Frequency (MHz)**	600.17	**Nucleus**	1H	**Number of Transients**	8	**Origin**	ECA
**Original Points Count**	32768	**Owner**	delta	**Points Count**	131072	**Pulse Sequence**	proton.jxp
**Solvent**	D2O	**Spectrum Offset (Hz)**	3907.0901	**Sweep Width (Hz)**	11281.59	**Temperature (degree C)**	20.600

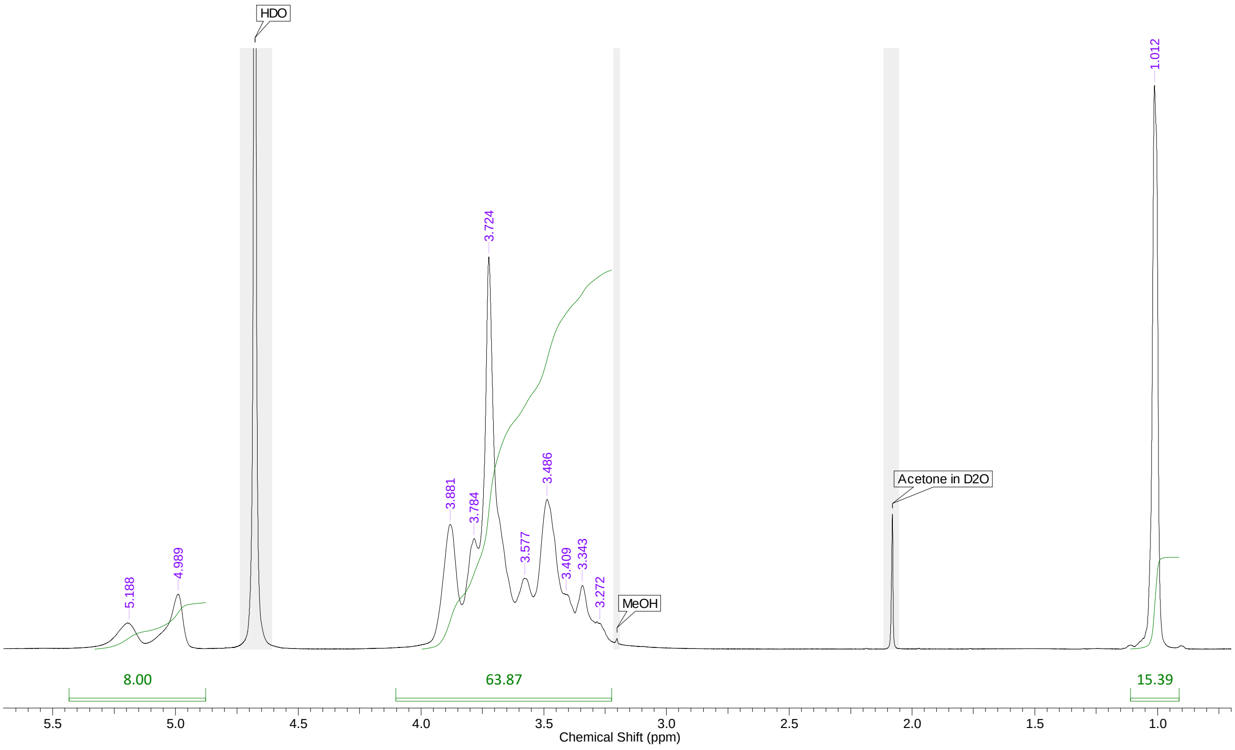

Figure S7: ^{1}H NMR of HP-γ-CD, prepared in ball mill, DS ≈ 5.1, entry 5 of Table S1

**Acquisition Time (sec)**	2.9046	**Comment**	single_pulse	**Date**	30 Oct 2018 11:53:58		
**Date Stamp**	30 Oct 2018 11:53:25			**File Name**	E:\Documents\!!Notebooks\!BM_Reactions\NMR\HPxCD\4SI\proton\HPxCD_LJ05_Proton-1-1.esp		
**Frequency (MHz)**	600.17	**Nucleus**	1H	**Number of Transients**	8	**Origin**	ECA
**Original Points Count**	32768	**Owner**	delta	**Points Count**	131072	**Pulse Sequence**	proton.jxp
**Solvent**	D2O	**Spectrum Offset (Hz)**	3901.0518	**Sweep Width (Hz)**	11281.59	**Temperature (degree C)**	20.300

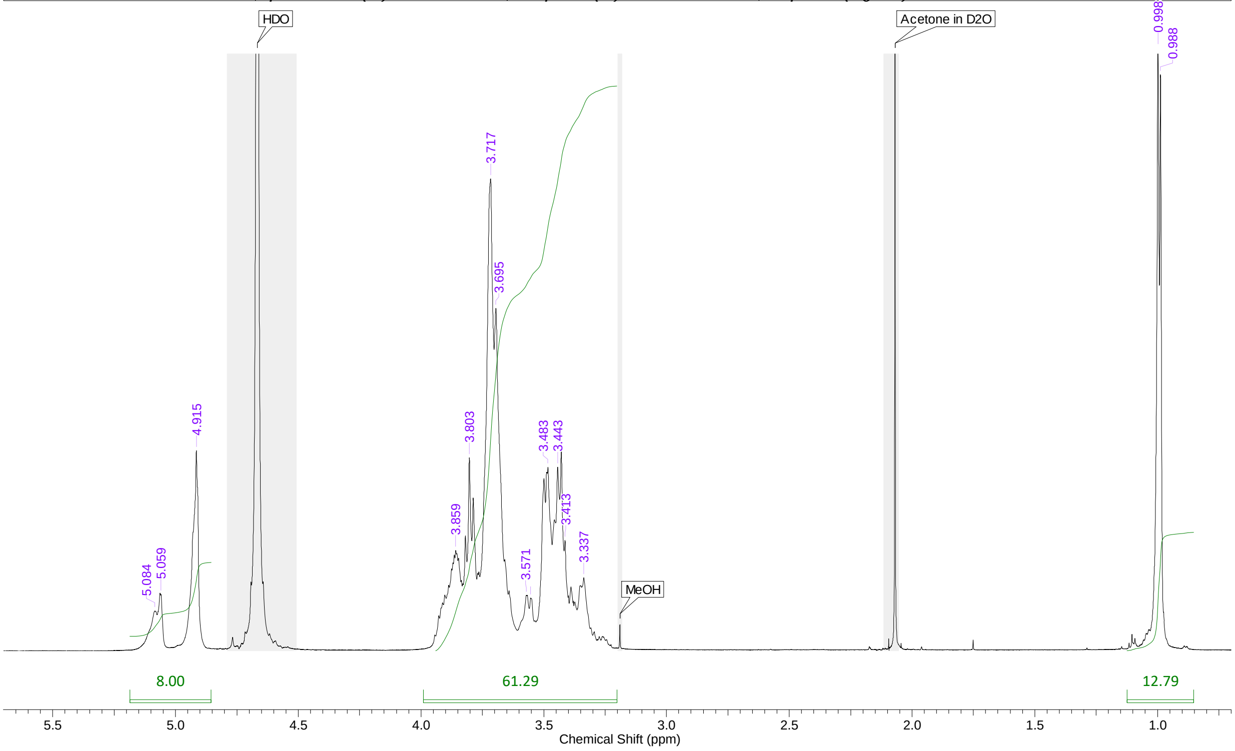

Figure S8: ^{1}H NMR of HP-γ-CD, prepared in ball mill, DS ≈ 4.3, entry 6 of Table S1

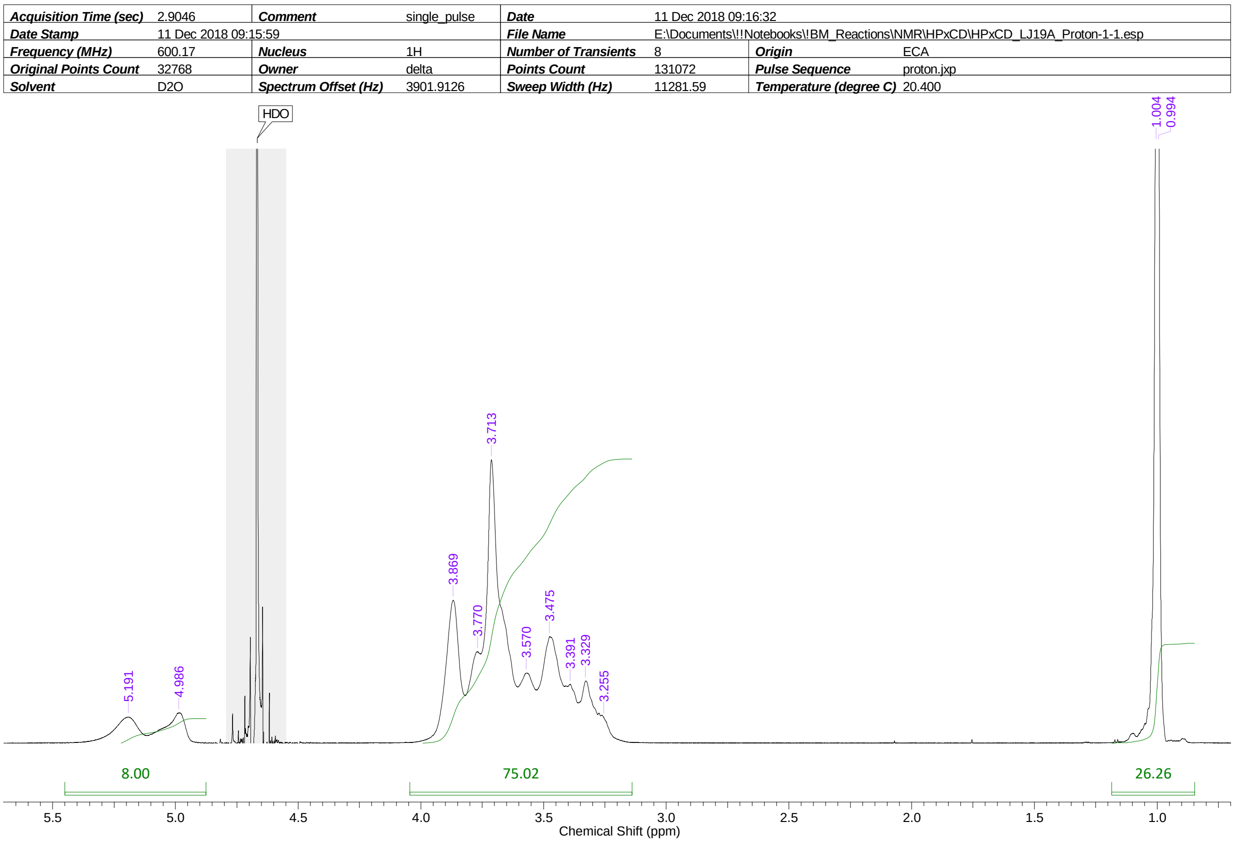

Figure S9: ^{1}H NMR of HP-γ-CD, prepared in solution, DS ≈ 8.8, entry 7 of Table S1

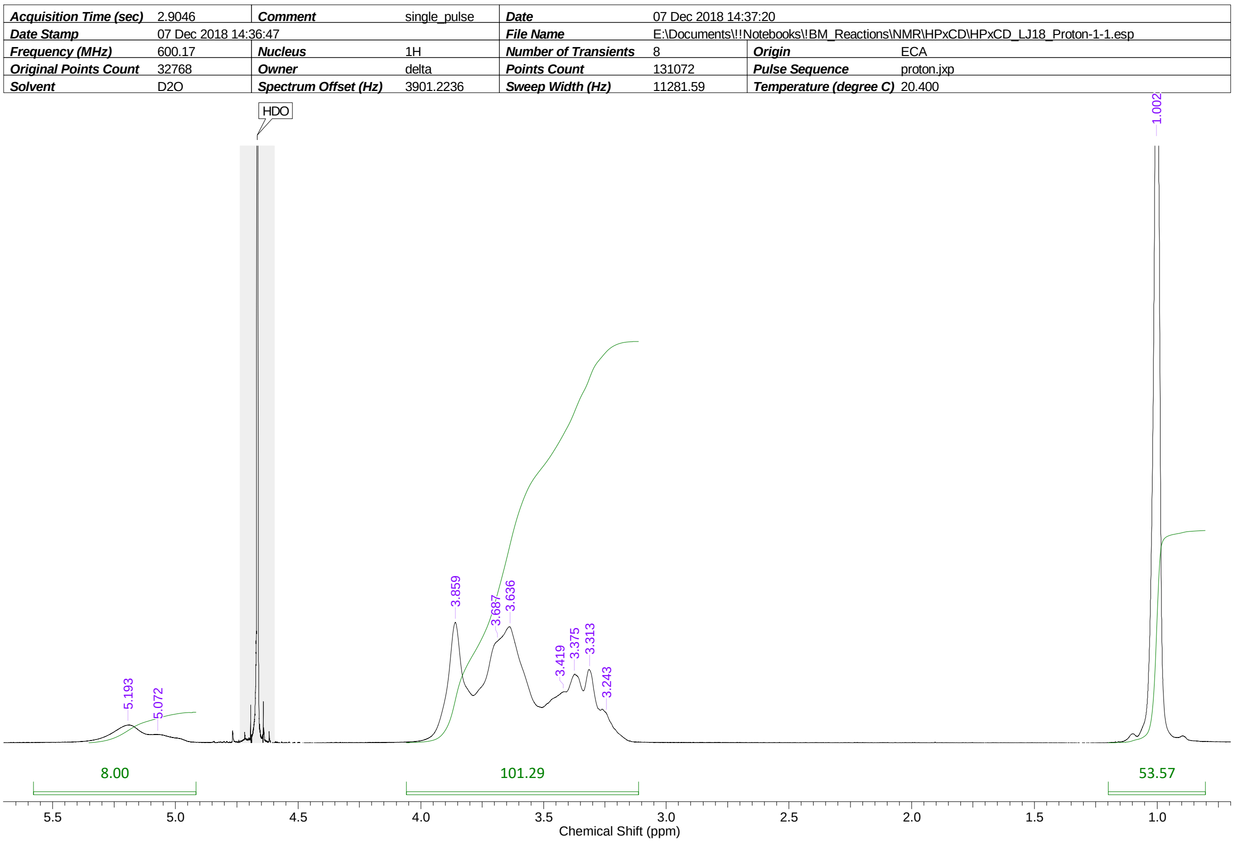

Figure S10: ^{1}H NMR of HP-γ-CD, prepared in solution, DS ≈ 17.6, entry 8 of Table S1

Acquisition Time (sec)	2.9046	***Comment***	single_pulse	***Date***	05 Dec 2018 11:42:49		
Date Stamp	05 Dec 2018 11:42:16						
File Name	E:\Documents\!!Notebooks\!BM_Reactions\NMR\HPxCD\4SI\GPTSbCD_rpt600 LJ07_Proton-1-1_a.esp					***Frequency (MHz)***	600.17
Nucleus	1H	***Number of Transients***	8	***Origin***	ECA	***Original Points Count***	32768
Owner	delta	***Points Count***	131072	***Pulse Sequence***	proton.jxp	***Solvent***	D2O
Spectrum Offset (Hz)	3902.0845	***Sweep Width (Hz)***	11281.59	***Temperature (degree C)***	19.600		

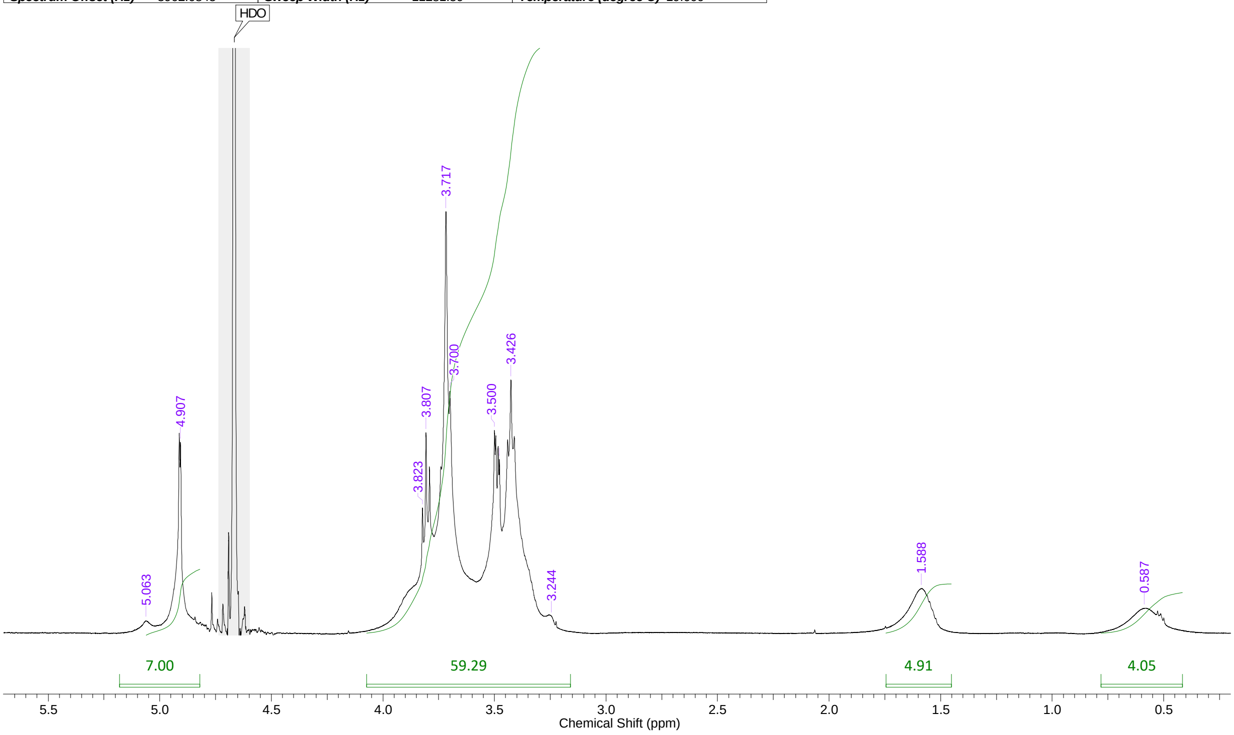

Figure S11: ^{1}H NMR of GPTS-β-CD, prepared in solution, DS ≈ 2.3-2.6, entry 15 of Table S1

Acquisition Time (sec)	3.6438	***Comment***	GPTSCD-LJ02_1H_D2O_100119_25617_rg=00			***Date***	10 Jan 2019 12:54:40
Date Stamp	10 Jan 2019 12:54:40			***File Name***	E:\Documents\!!!!!UniTo_publ\!!!!2018_BJOC_Epoxy\HPxCD\GPTSCD-LJ02\1\fid		
Frequency (MHz)	300.13	***Nucleus***	1H	***Number of Transients***	16	***Origin***	spect
Original Points Count	16384	***Owner***	root	***Points Count***	131072	***Pulse Sequence***	zg
Receiver Gain	1000.00	***SW(cyclical) (Hz)***	4496.40	***Solvent***	CHLOROFORM-d		
Spectrum Offset (Hz)	1942.8433	***Spectrum Type***	STANDARD	***Sweep Width (Hz)***	4496.37	***Temperature (degree C)***	19.260

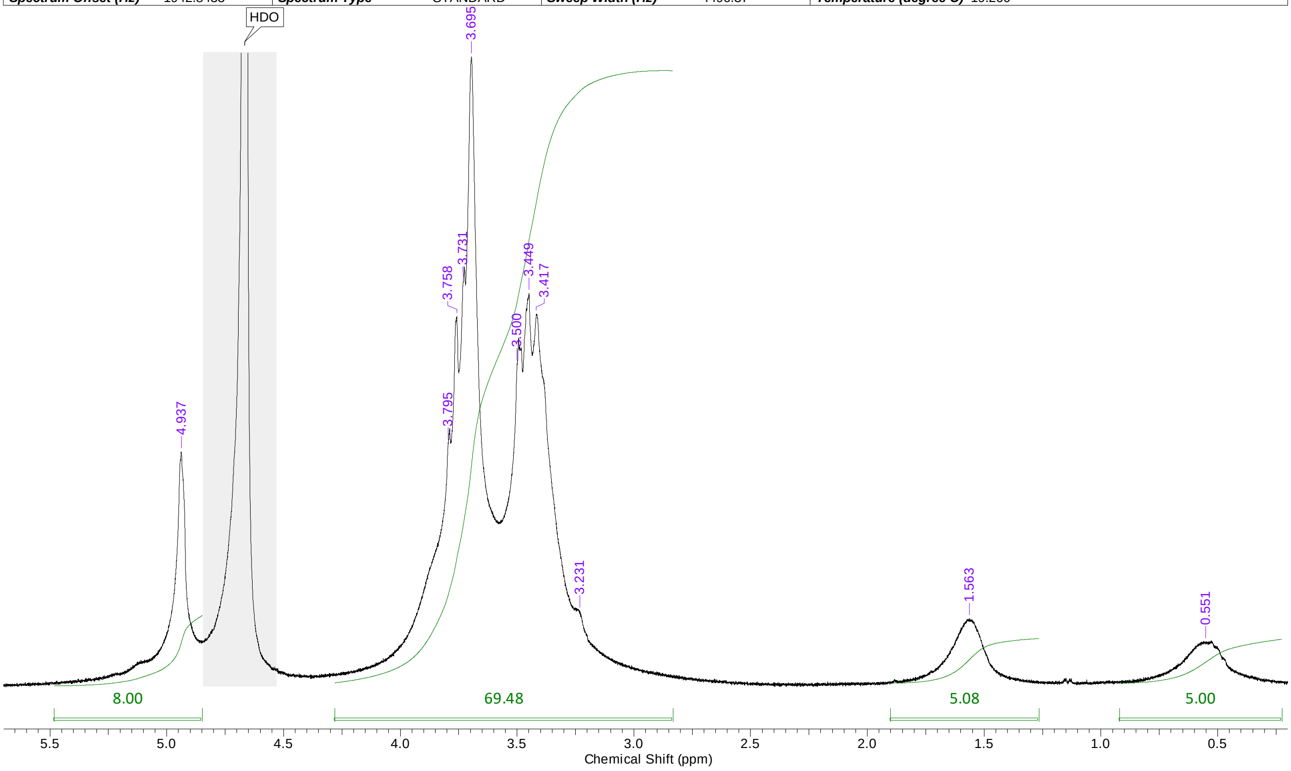

Figure S12: ^{1}H NMR of GPTS-γ-CD, prepared in solution, DS ≈ 2.5, entry 17 of Table S1

Acquisition Time (sec)	0.6921	***Comment***	single pulse decoupled gated NOE			***Date***	26 Oct 2018 11:37:23
Date Stamp	23 Oct 2018 10:02:55			***File Name***	E:\Documents\!!Notebooks\!BM_Reactions\NMR\HPxCD\Carbon\HPxCD_LJ00bSolution_Carbon-1-4.esp		
Frequency (MHz)	150.91	***Nucleus***	13C	***Number of Transients***	512	***Origin***	ECA
Original Points Count	32768	***Owner***	delta	***Points Count***	1048576	***Pulse Sequence***	carbon.jxp
Solvent	D2O	***Spectrum Offset (Hz)***	16600.4766	***Sweep Width (Hz)***	47348.49	***Temperature (degree C)***	21.200

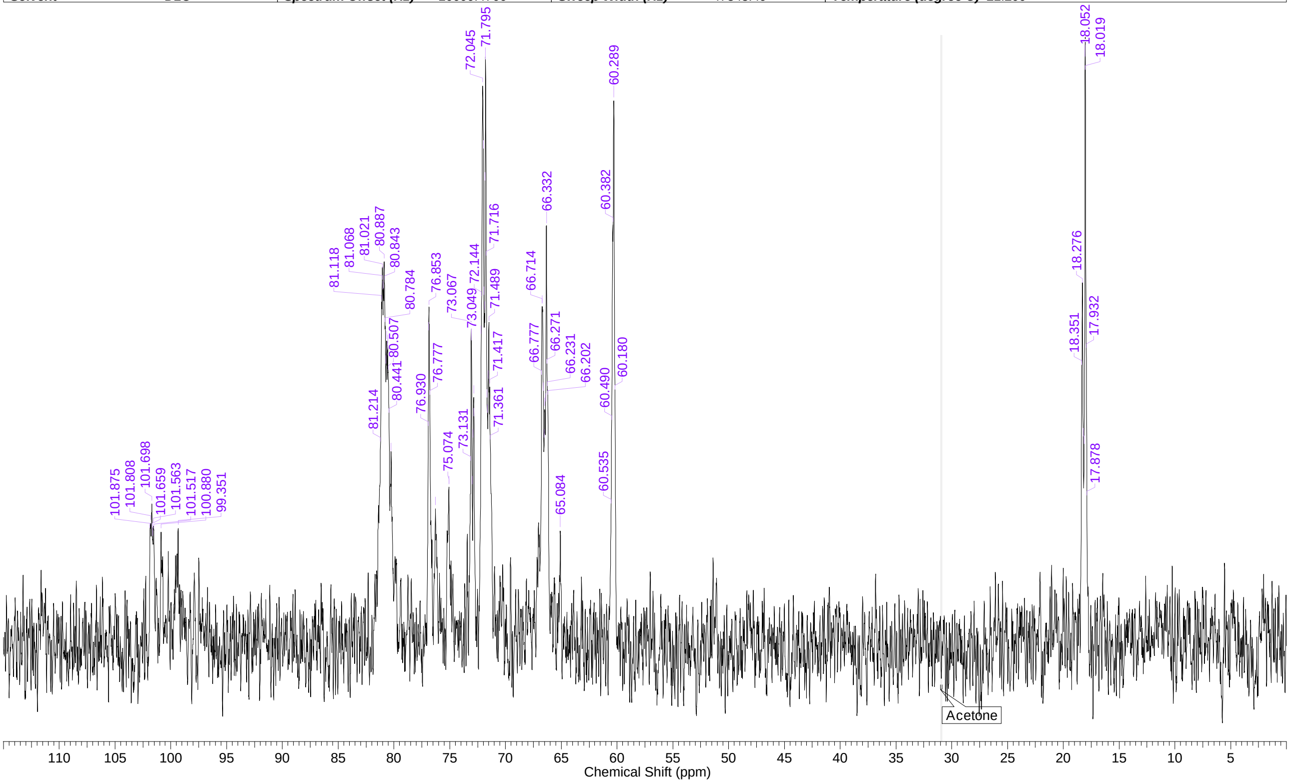

Figure S13: ^{13}C NMR of HP-β-CD, prepared in solution, DS ≈ 4.4

Acquisition Time (sec)	0.6921	***Comment***	single pulse decoupled gated NOE			***Date***	30 Oct 2018 11:48:21
Date Stamp	30 Oct 2018 11:30:44			***File Name***	E:\Documents\!!Notebooks\!BM_Reactions\NMR\HPxCD\Carbon\HPxCD_LJ01_Carbon-1-1.esp		
Frequency (MHz)	150.91	***Nucleus***	13C	***Number of Transients***	512	***Origin***	ECA
Original Points Count	32768	***Owner***	delta	***Points Count***	1048576	***Pulse Sequence***	carbon.jxp
Solvent	D2O	***Spectrum Offset (Hz)***	16694.3477	***Sweep Width (Hz)***	47348.49	***Temperature (degree C)***	20.400

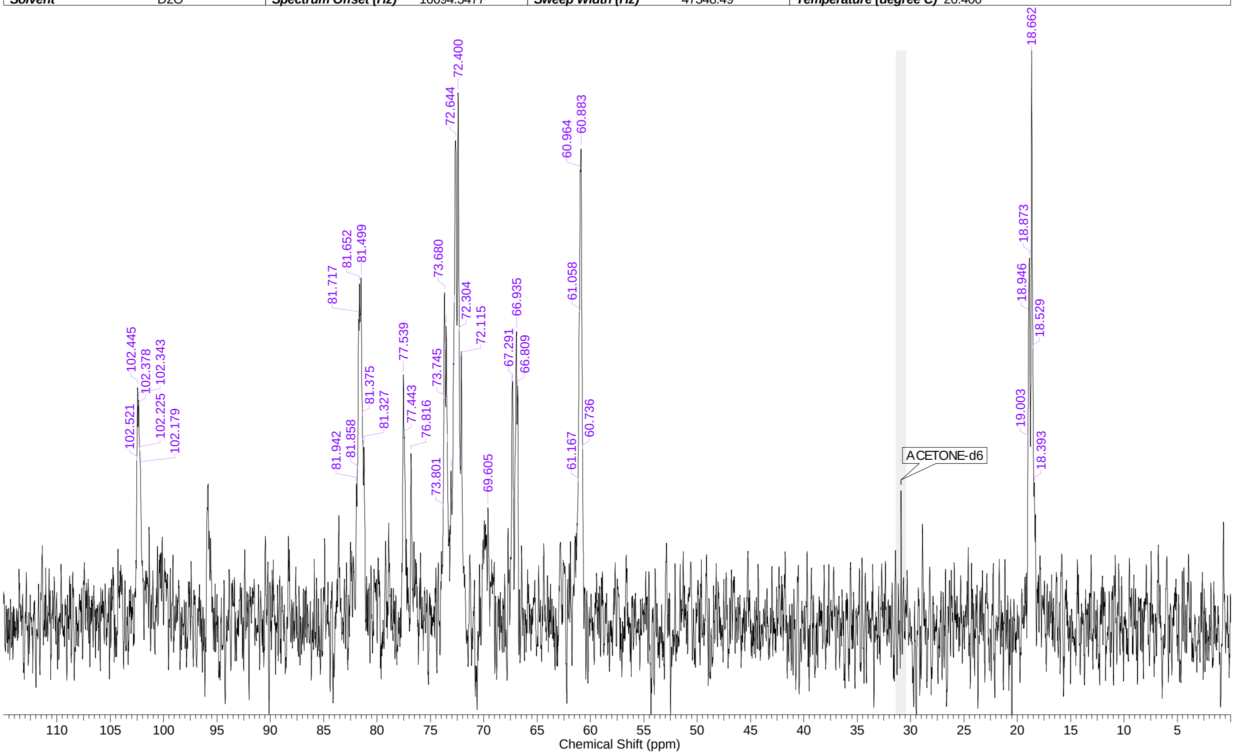

Figure S14: ^{13}C NMR of HP-β-CD, prepared in ball mill, DS ≈ 4.4, entry 1 of Table S1

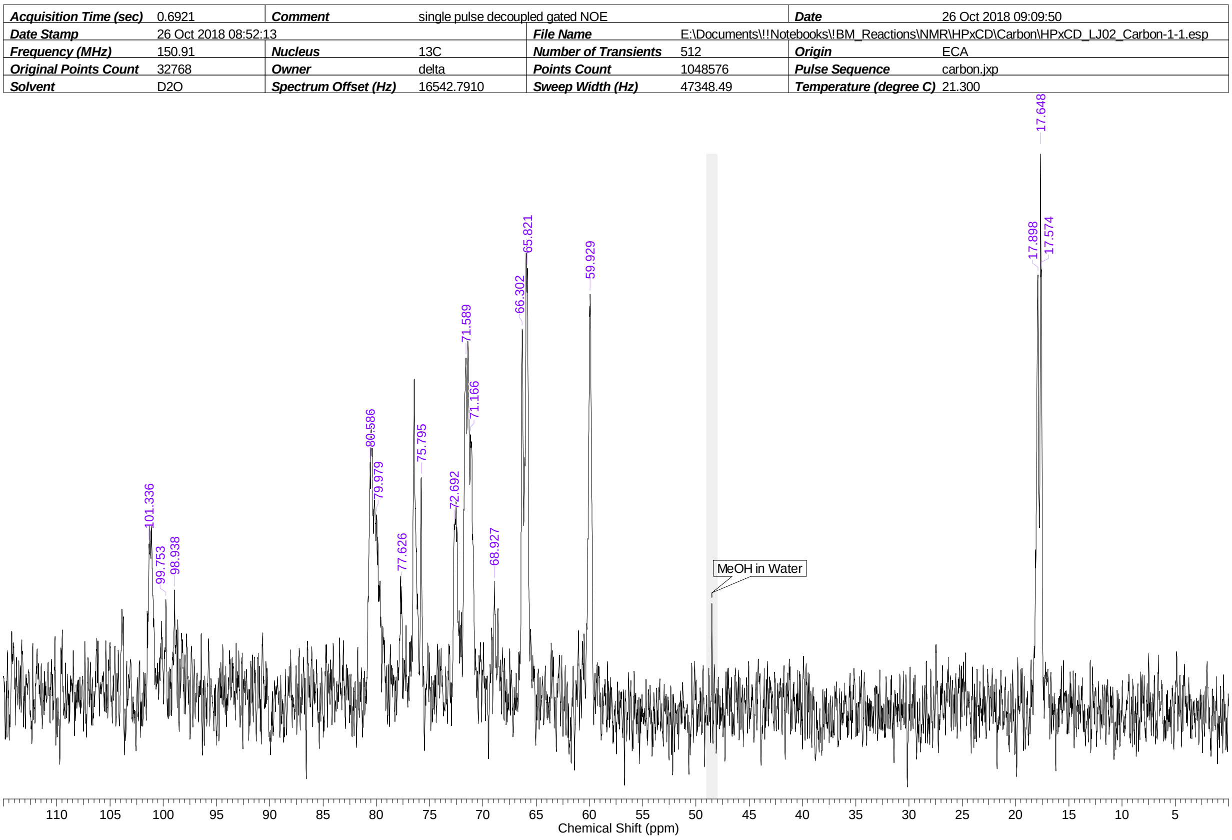

Figure S15: ^{13}C NMR of HP-β-CD, prepared in ball mill, DS ≈ 5.6, entry 2 of Table S1

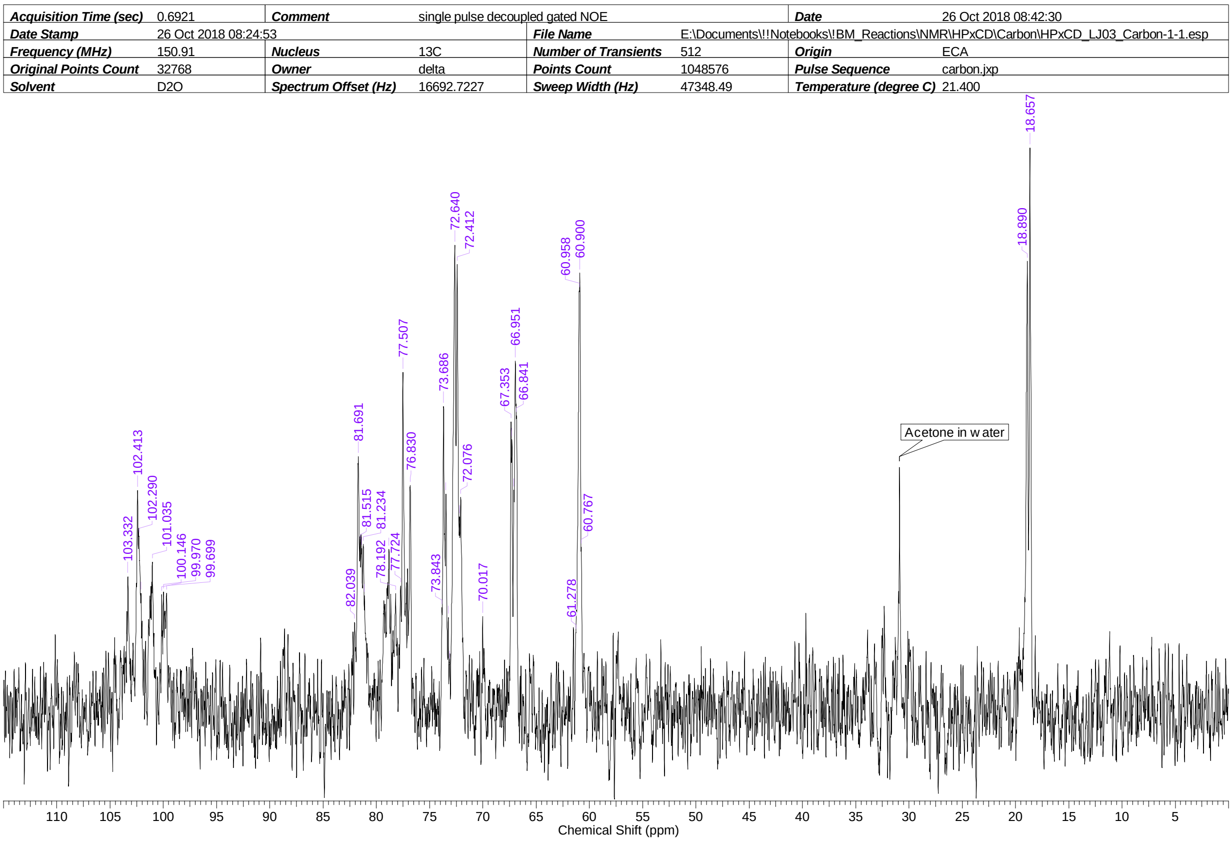

Figure S16: ^{13}C NMR of HP-β-CD, prepared in ball mill, DS ≈ 5.3, entry 3 of Table S1

Acquisition Time (sec)	0.6921	***Comment***	single pulse decoupled gated NOE			***Date***	30 Oct 2018 12:14:04
Date Stamp	30 Oct 2018 11:56:27			***File Name***	E:\Documents\!!Notebooks\!BM_Reactions\NMR\HPxCD\Carbon\HPxCD_LJ05_Carbon-1-1.esp		
Frequency (MHz)	150.91	***Nucleus***	13C	***Number of Transients***	512	***Origin***	ECA
Original Points Count	32768	***Owner***	delta	***Points Count***	1048576	***Pulse Sequence***	carbon.jxp
Solvent	D2O	***Spectrum Offset (Hz)***	16695.2969	***Sweep Width (Hz)***	47348.49	***Temperature (degree C)***	20.400

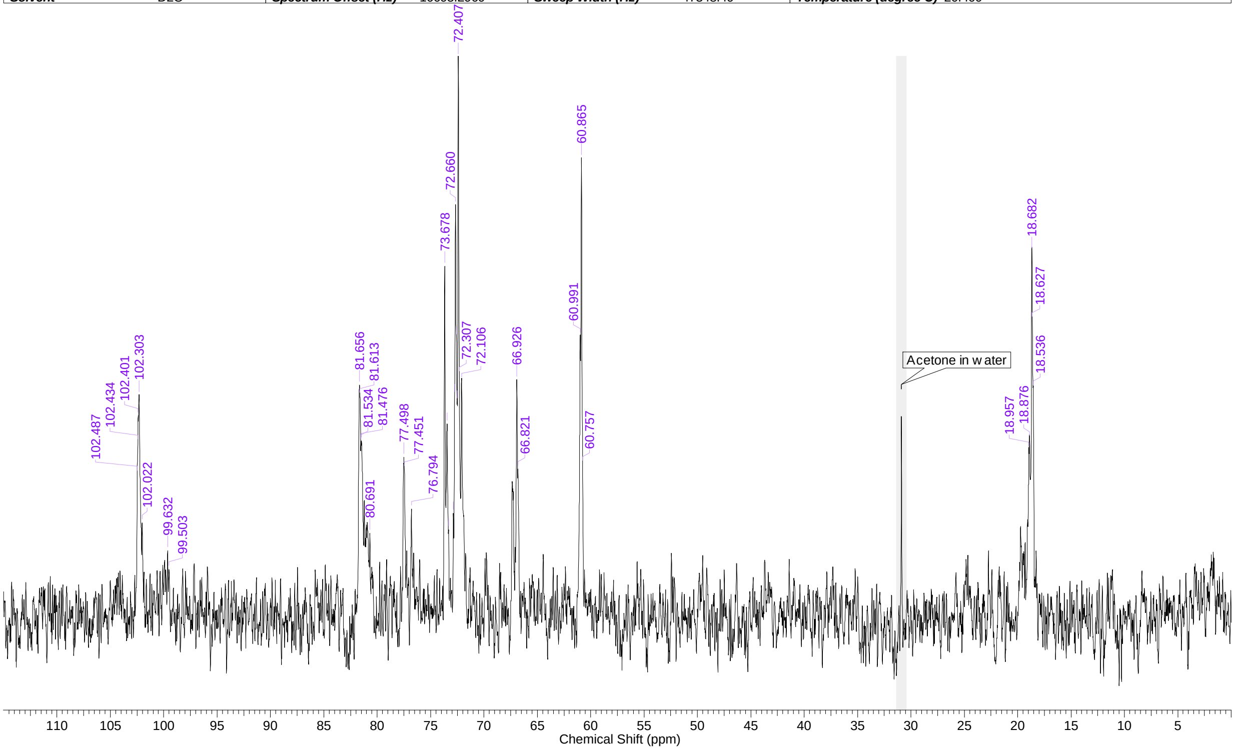

Figure S17: ^{13}C NMR of HP-β-CD, prepared in ball mill, DS ≈ 3.7, entry 4 of Table S1

Acquisition Time (sec)	0.6921	***Comment***	single pulse decoupled gated NOE			***Date***	17 Dec 2018 11:37:35
Date Stamp	17 Dec 2018 11:19:58			***File Name***	E:\Documents\!!Notebooks\!BM_Reactions\NMR\HPxCD\HPxCD_LJ00gSolution_Carbon-1-1.esp		
Frequency (MHz)	150.91	***Nucleus***	13C	***Number of Transients***	512	***Origin***	ECA
Original Points Count	32768	***Owner***	delta	***Points Count***	262144	***Pulse Sequence***	carbon.jxp
Solvent	D2O	***Spectrum Offset (Hz)***	16600.4766	***Sweep Width (Hz)***	47348.49	***Temperature (degree C)***	18.400

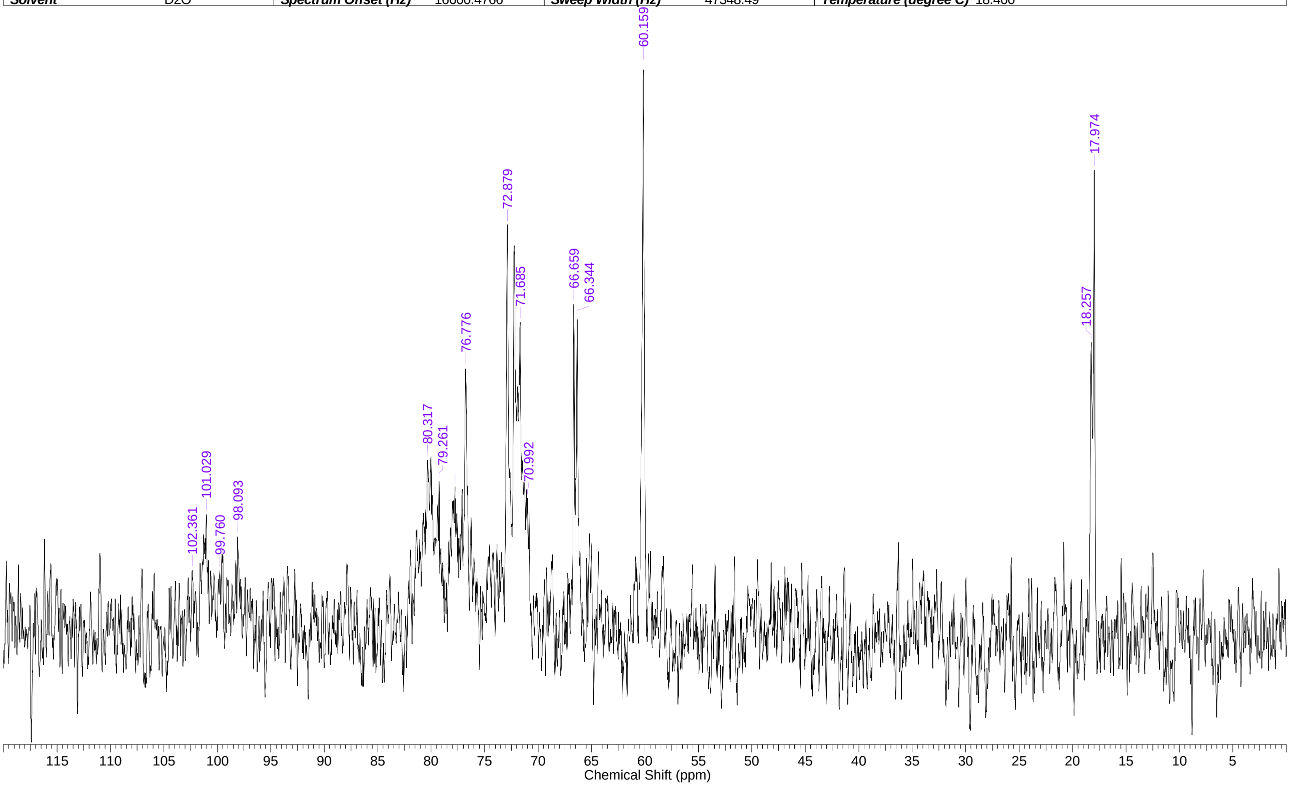

Figure S18: ^{13}C NMR of HP-γ-CD, prepared in solution, DS ≈ 4.5

Acquisition Time (sec)	0.6921	***Comment***	single pulse decoupled gated NOE			***Date***	30 Oct 2018 12:41:13
Date Stamp	30 Oct 2018 12:23:36			***File Name***	E:\Documents\!!Notebooks\!BM_Reactions\NMR\HPxCD\HPXCD_LJ04_Carbon-1-1.esp		
Frequency (MHz)	150.91	***Nucleus***	13C	***Number of Transients***	512	***Origin***	ECA
Original Points Count	32768	***Owner***	delta	***Points Count***	1048576	***Pulse Sequence***	carbon.jxp
Solvent	D2O	***Spectrum Offset (Hz)***	16695.8379	***Sweep Width (Hz)***	47348.49	***Temperature (degree C)***	20.900

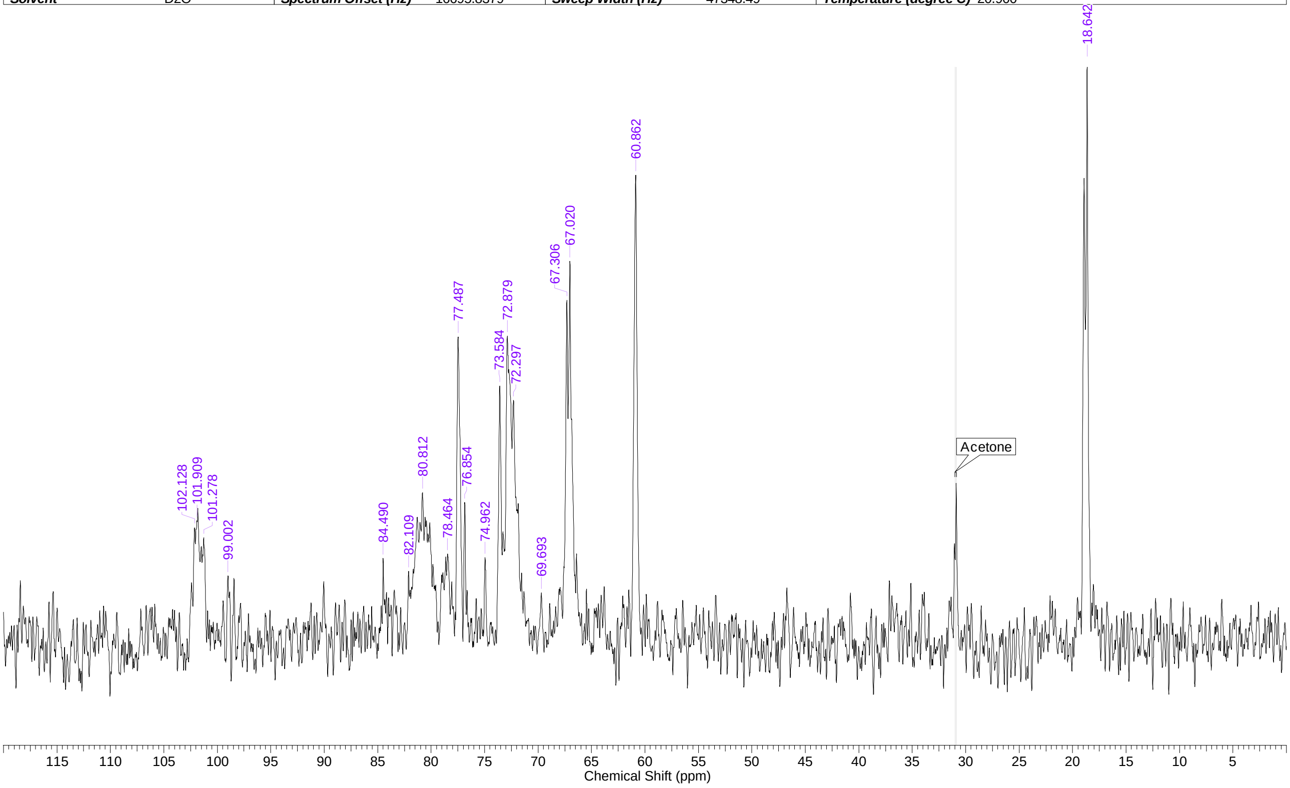

Figure S19: ^{13}C NMR of HP-γ-CD, prepared in ball mill, DS ≈ 5.1, entry 5 of Table S1

Acquisition Time (sec)	0.6921	***Comment***	single pulse decoupled gated NOE			***Date***	22 Nov 2018 09:50:00
Date Stamp	22 Nov 2018 09:32:23			***File Name***	E:\Documents\!!Notebooks\!BM_Reactions\NMR\HPxCD\Carbon\HPxCD_LJ06_Carbon-1-1.esp		
Frequency (MHz)	150.91	***Nucleus***	13C	***Number of Transients***	512	***Origin***	ECA
Original Points Count	32768	***Owner***	delta	***Points Count***	1048576	***Pulse Sequence***	carbon.jxp
Solvent	D2O	***Spectrum Offset (Hz)***	16695.1152	***Sweep Width (Hz)***	47348.49	***Temperature (degree C)***	20.700

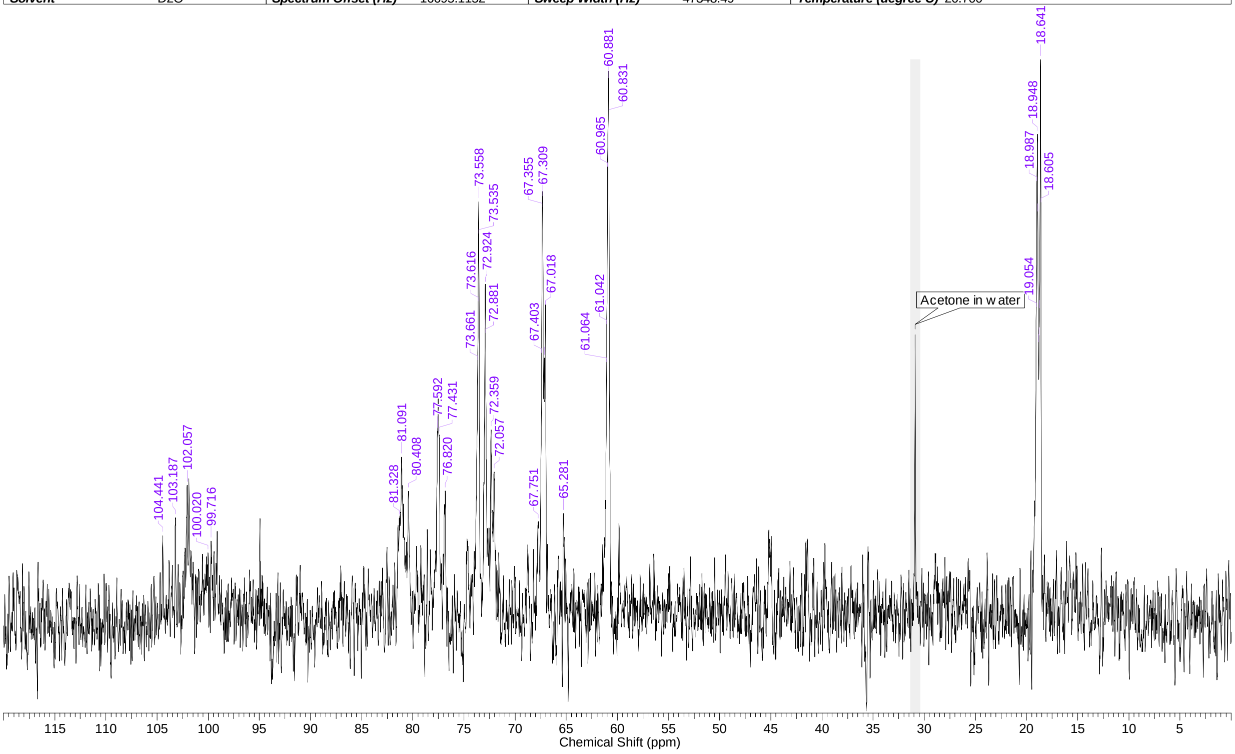

Figure S20: ^{13}C NMR of HP-γ-CD, prepared in ball mill, DS ≈ 4.3, entry 6 of Table S1

Acquisition Time (sec)	0.6921	***Comment***	single pulse decoupled gated NOE			***Date***	11 Dec 2018 09:36:30
Date Stamp	11 Dec 2018 09:18:53			***File Name***	E:\Documents\!!Notebooks\!BM_Reactions\NMR\HPxCD\HPxCD_LJ19A_Carbon-1-1.esp		
Frequency (MHz)	150.91	***Nucleus***	13C	***Number of Transients***	512	***Origin***	ECA
Original Points Count	32768	***Owner***	delta	***Points Count***	524288	***Pulse Sequence***	carbon.jxp
Solvent	D2O	***Spectrum Offset (Hz)***	16600.4766	***Sweep Width (Hz)***	47348.49	***Temperature (degree C)***	20.500

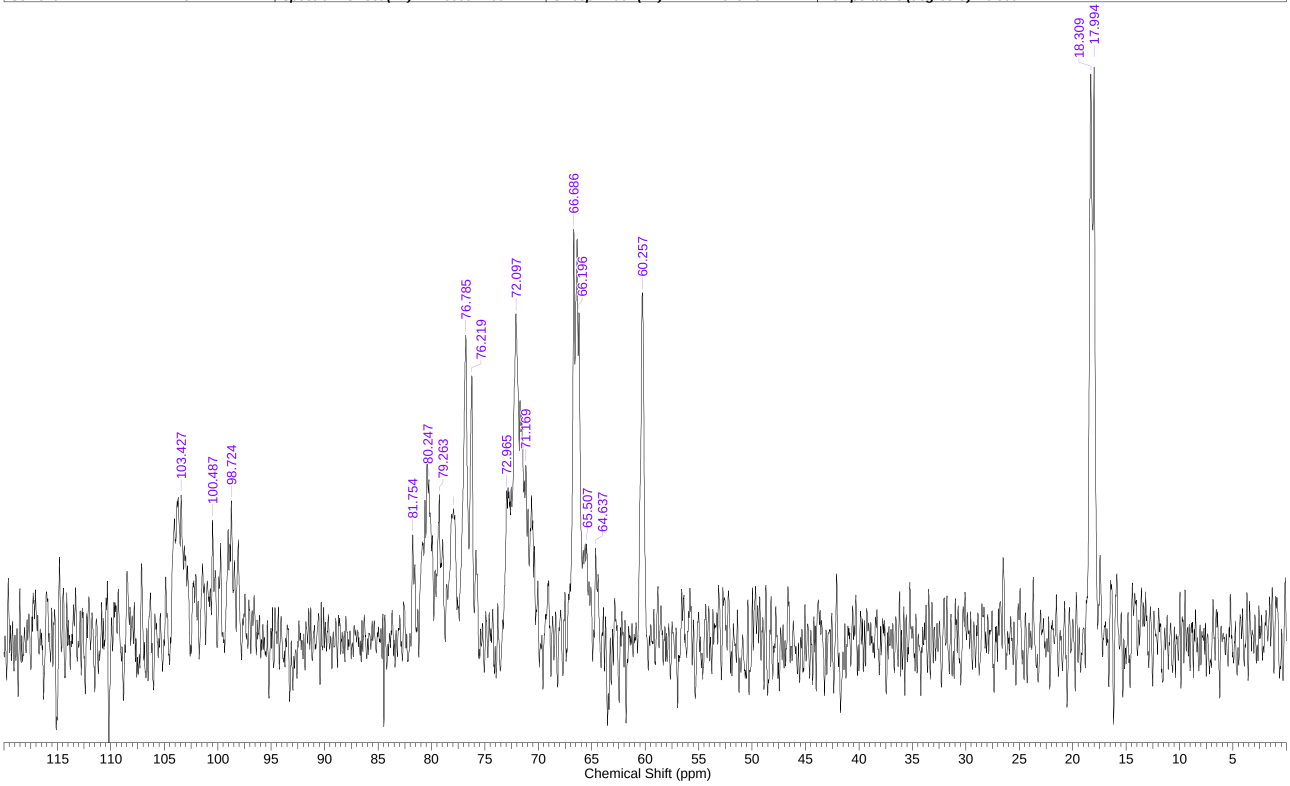

Figure S21: ^{13}C NMR of HP-γ-CD, prepared in solution, DS ≈ 8.8, entry 7 of Table S1

Acquisition Time (sec)	0.6921	***Comment***	single pulse decoupled gated NOE			***Date***	07 Dec 2018 14:57:14
Date Stamp	07 Dec 2018 14:39:37			***File Name***	E:\Documents\!!Notebooks\!BM_Reactions\NMR\HPxCD\HPxCD_LJ18_Carbon-1-1.esp		
Frequency (MHz)	150.91	***Nucleus***	13C	***Number of Transients***	512	***Origin***	ECA
Original Points Count	32768	***Owner***	delta	***Points Count***	262144	***Pulse Sequence***	carbon.jxp
Solvent	D2O	***Spectrum Offset (Hz)***	16600.4766	***Sweep Width (Hz)***	47348.49	***Temperature (degree C)***	20.700

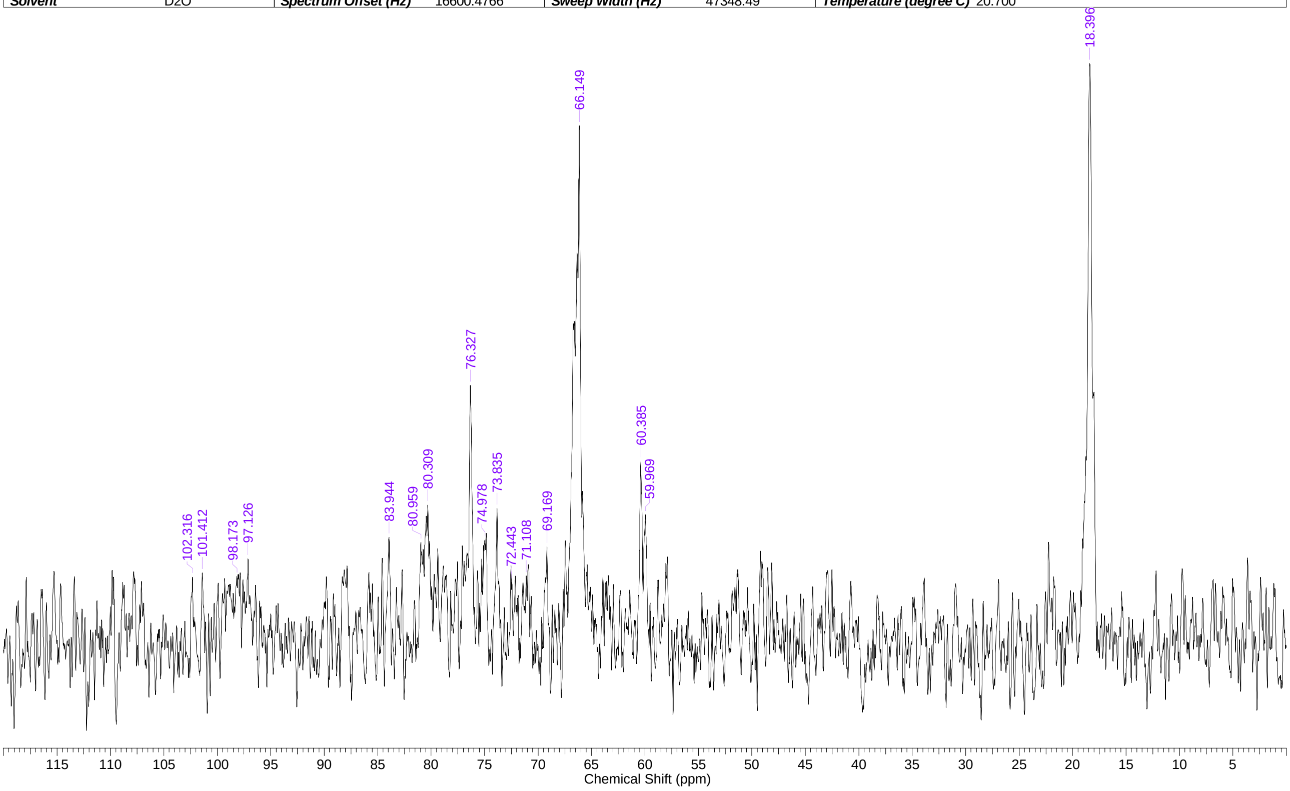

Figure S22: ^{13}C NMR of HP-γ-CD, prepared in solution, DS ≈ 17.6, entry 8 of Table S1

**Acquisition Time (sec)**	0.6921	**Comment**	single pulse decoupled gated NOE			**Date**	05 Dec 2018 12:02:56
**Date Stamp**	05 Dec 2018 11:45:19						
**File Name**	E:\Documents\!!!!!UniTo_publ\!!!!2018_BJOC_Epoxy\HPxCD\GPTSbCD_rpt600 LJ07_Carbon-1-1.jdf					**Frequency (MHz)**	150.91
**Nucleus**	13C	**Number of Transients**	512	**Origin**	ECA	**Original Points Count**	32768
**Owner**	delta	**Points Count**	262144	**Pulse Sequence**	carbon.jxp	**Solvent**	D2O
**Spectrum Offset (Hz)**	16600.4766	**Sweep Width (Hz)**	47348.49	**Temperature (degree C)**	20.300		

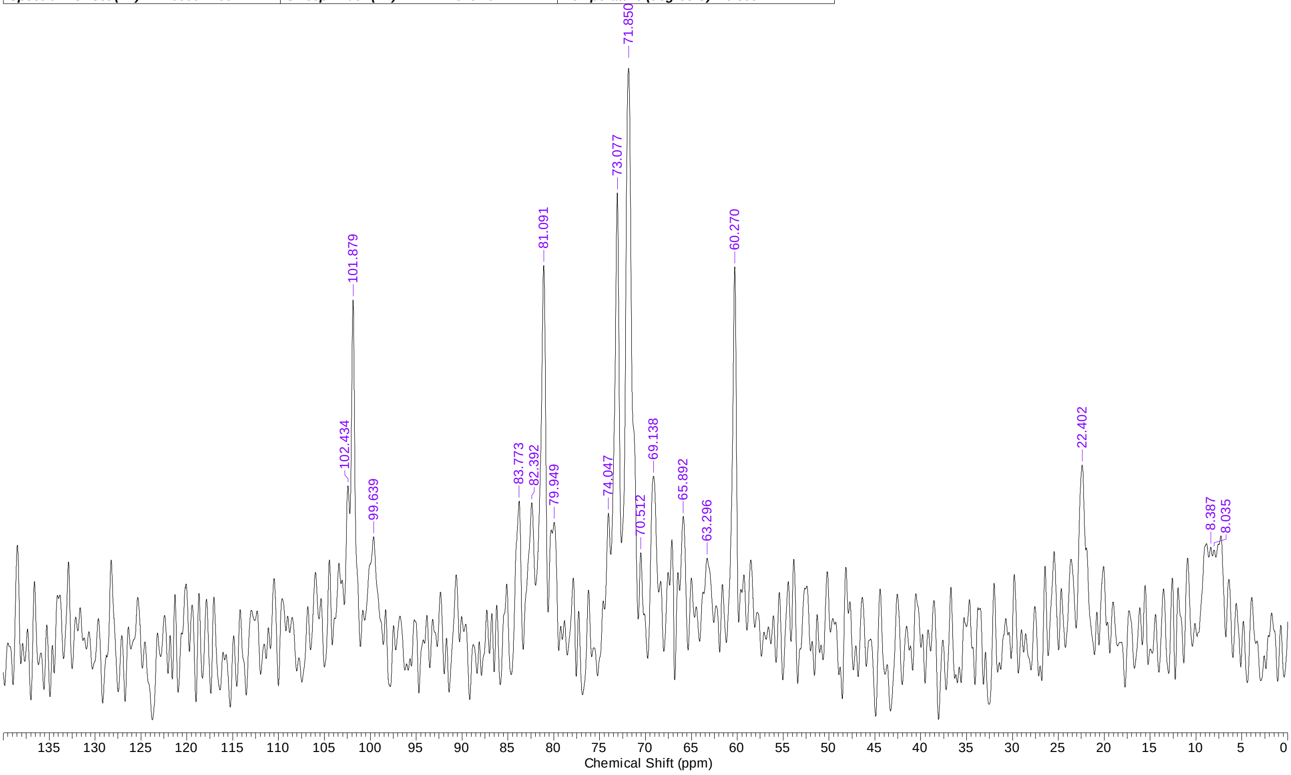

Figure S23: ^{13}C NMR of GPTS-β-CD, prepared in solution, DS ≈ 2.3-2.6, entry 15 of Table S1

**Acquisition Time (sec)**	0.6921	**Comment**	single pulse decoupled gated NOE			**Date**	21 Jan 2019 09:49:06
**Date Stamp**	21 Jan 2019 09:31:29			**File Name**	E:\Documents\!!!!!UniTo_publ\!!!!2018_BJOC_Epoxy\HPxCD\4SI\GPTS_LJ02S_Carbon-1-1.jdf		
**Frequency (MHz)**	150.91	**Nucleus**	13C	**Number of Transients**	512	**Origin**	scc
**Original Points Count**	32768	**Owner**	delta	**Points Count**	262144	**Pulse Sequence**	carbon.jxp
**Solvent**	D2O	**Spectrum Offset (Hz)**	16600.4766	**Sweep Width (Hz)**	47348.49	**Temperature (degree C)**	20.800

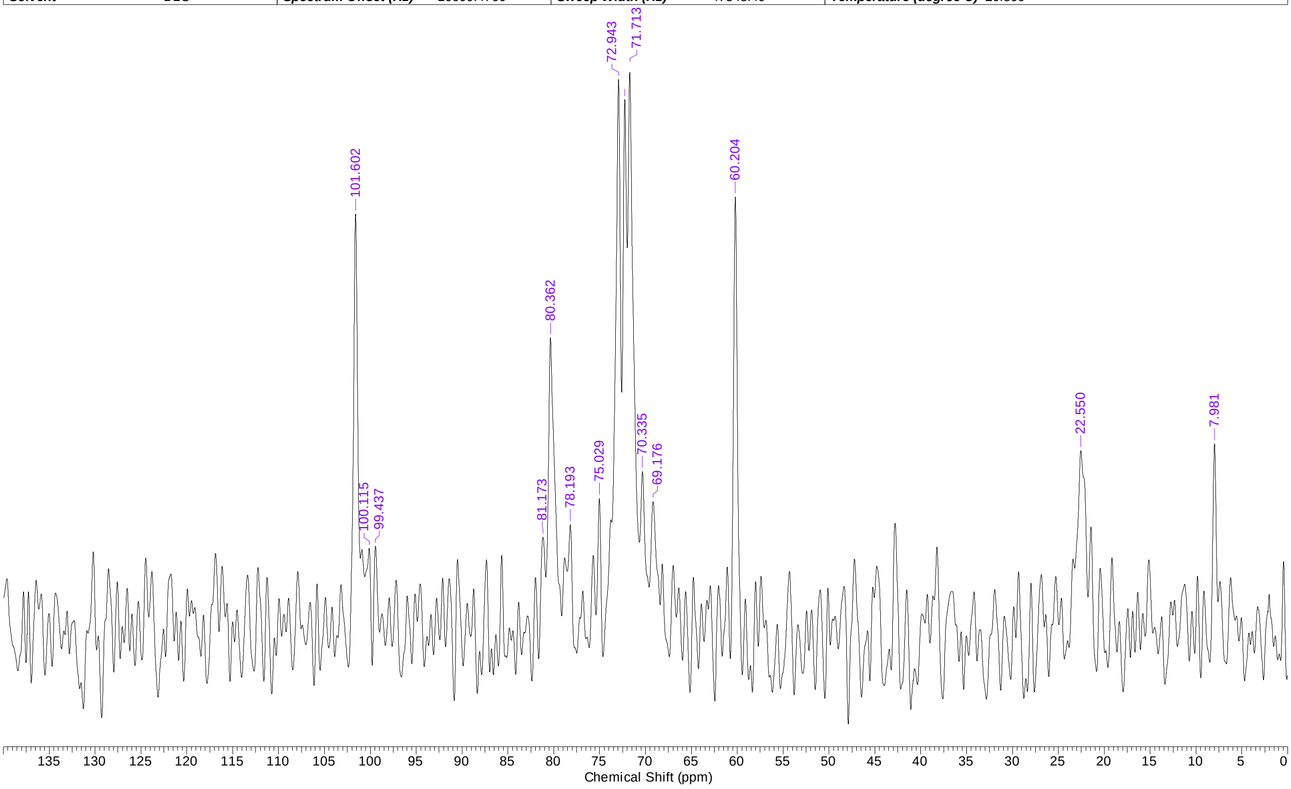

Figure S24: ^{13}C NMR of GPTS-γ-CD, prepared in solution, DS ≈ 2.5, entry 17 of Table S1

mMass Report: HPxCD_LJ00bSolution_Na(+)

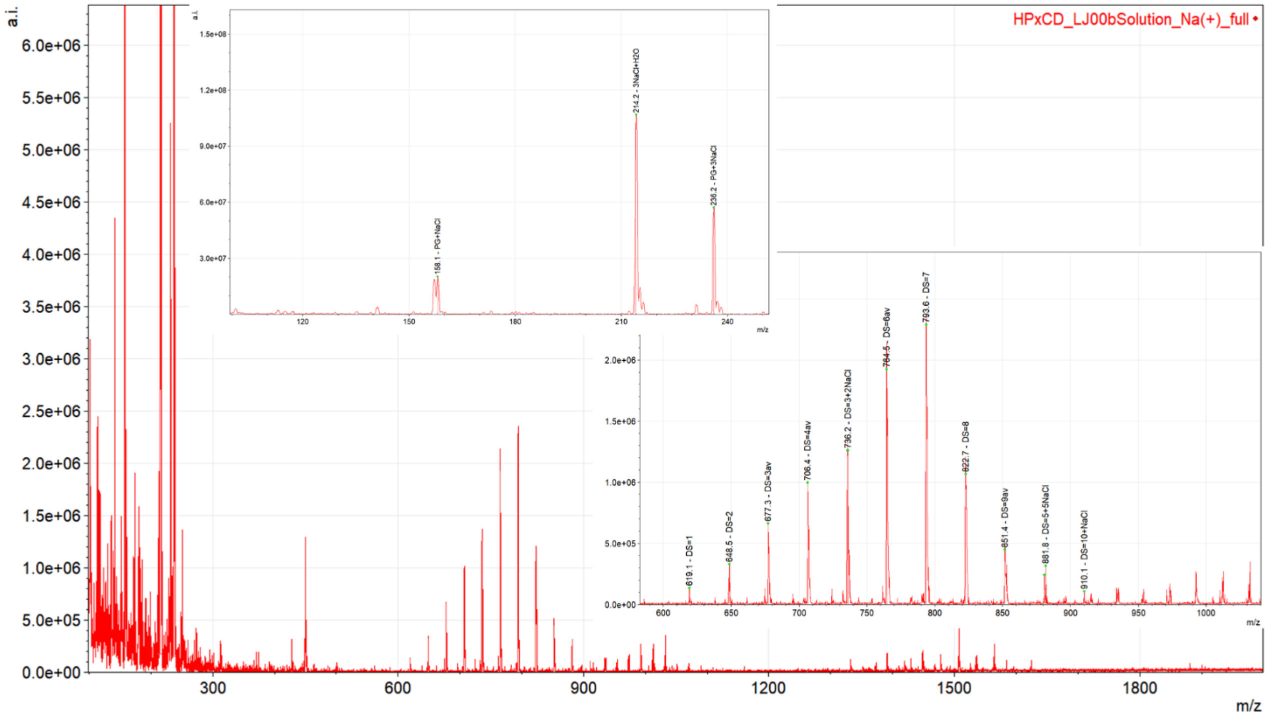

Annotations

Meas. m/z	Calc. m/z	δ (Da)	Int.	Rel. Int. (%)	z	Annotation	Formula
619.1478	619.1950	0.59	19920760	18.67	1	PG+NaCl	(HOC3H7O)1(NaCl)1Na
648.5330	648.5614	-0.66	106718751	100.00	1	3NaCl+H2O	(HOC3H7O)0(NaCl)3(H2O)Na
648.5330	648.7430	-0.20	56964063	53.38	2	PG+6NaCl	(HOC3H7O)1(NaCl)6Na2
677.2554	677.1953	-0.05	127508	5.59	2	DS=1	βCD((C3H6O))1Na2
677.2554	677.6011	-0.03	322055	14.13	2	DS=2	βCD((C3H6O))2Na2
677.2554	677.2369	-0.21	322055	14.13	2	DS=1+NaCl	βCD((C3H6O))1(NaCl)1Na2
706.3877	706.6407	0.06	656751	28.82	2	DS=2+NaCl	βCD((C3H6O))2(NaCl)1Na2
706.3877	706.2162	-0.35	656751	28.82	2	DS=3av	βCD((C3H6O))3Na2
706.3877	707.2657	0.02	656751	28.82	2	DS=3	βCD((C3H6O))3Na2
735.5659	735.6803	-0.25	988569	43.38	2	DS=4av	βCD((C3H6O))4Na2
736.1586	736.0434	0.17	988569	43.38	2	DS=3+NaCl	βCD((C3H6O))3(NaCl)1Na2
764.5388	764.7200	-0.88	988569	43.38	2	DS=4	βCD((C3H6O))4Na2
764.5388	764.2997	-0.11	1253439	55.00	2	DS=5	βCD((C3H6O))5Na2
764.5388	764.9015	0.12	1253439	55.00	2	DS=3+2NaCl	βCD((C3H6O))3(NaCl)2Na2
764.5388	764.7200	-0.18	1917492	84.13	2	DS=6	βCD((C3H6O))6Na2
793.5650	793.7596	0.24	1917492	84.13	2	DS=6	βCD((C3H6O))6Na2
822.6593	822.7992	-0.36	1917492	84.13	2	DS=5+NaCl	βCD((C3H6O))5(NaCl)1Na2
851.4263	852.8468	-0.18	1917492	84.13	2	DS=6av	βCD((C3H6O))6Na2
851.4263	851.3625	-0.19	2279115	100.00	2	DS=7	βCD((C3H6O))7Na2
851.4263	851.3209	-0.14	1056473	46.35	2	DS=8	βCD((C3H6O))8Na2
880.5594	880.8785	-1.42	439051	19.26	2	DS=9av	βCD((C3H6O))9Na2
881.7559	881.7861	0.06	439051	19.26	2	DS=9	βCD((C3H6O))9Na2
910.0811	910.0996	0.11	439051	19.26	2	DS=8+NaCl	βCD((C3H6O))8(NaCl)1Na2
619.1478	619.1950	-0.32	235206	10.32	2	DS=10	βCD((C3H6O))10Na2
648.5330	648.5614	-0.03	307266	13.48	2	DS=5+5NaCl	βCD((C3H6O))5(NaCl)5Na2
648.5330	648.7430	-0.02	98678	4.33	2	DS=10+NaCl	βCD((C3H6O))10(NaCl)1Na2

Figure S25: ESIMS+ spectrum of HP-β-CD, prepared in solution, DS ≈ 4.4

mMass Report: HPxCD_LJ01_Na(+)

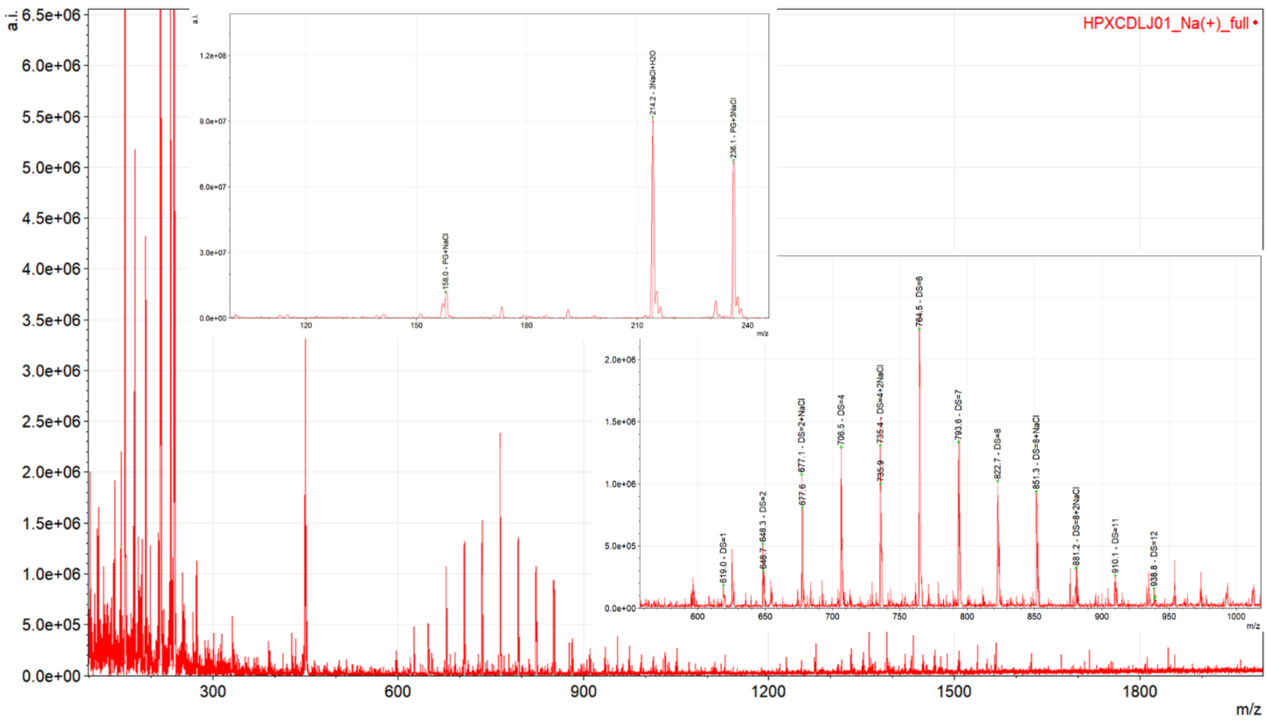

Annotations

Meas. m/z	Calc. m/z	δ (Da)	Int.	Rel. Int. (%)	z	Annotation	Formula
157.9510	157.5261	0.42	11956340	12.98	1	PG+NaCl	(HOC3H7O)1(NaCl)1Na
214.1693	214.8757	-0.71	92096838	100.00	1	3NaCl+H2O	(HOC3H7O)0(NaCl)3(H2O)Na
236.1077	236.3634	-0.26	72231795	78.43	2	PG+6NaCl	(HOC3H7O)1(NaCl)6Na2
619.0146	619.1950	-0.18	167043	7.50	2	DS=1	βCD((C3H6O))1Na2
648.2683	648.2160	0.05	496761	22.30	2	DS=2	βCD((C3H6O))2Na2
648.6614	648.7430	-0.08	278133	12.48	2	DS=1+NaCl	βCD((C3H6O))1(NaCl)1Na2
677.1205	677.1953	-0.07	1058518	47.51	2	DS=2+NaCl	βCD((C3H6O))2(NaCl)1Na2
677.6315	677.6011	0.03	790394	35.48	2	DS=3	βCD((C3H6O))3Na2
706.5121	706.2578	0.25	1274855	57.22	2	DS=4	βCD((C3H6O))4Na2
735.4001	735.2371	0.16	1289699	57.89	2	DS=4+2NaCl	βCD((C3H6O))4(NaCl)1Na2
735.9000	735.6803	0.22	1000000	44.89	2	DS=5	βCD((C3H6O))5Na2
764.5115	764.7200	-0.21	2227854	100.00	2	DS=6	βCD((C3H6O))6Na2
793.6056	793.7596	-0.15	1314285	58.99	2	DS=7	βCD((C3H6O))7Na2
822.6610	822.7992	-0.14	1000715	44.92	2	DS=8	βCD((C3H6O))8Na2
851.3192	851.3209	-0.00	918202	41.21	2	DS=8+NaCl	βCD((C3H6O))8(NaCl)1Na2
880.8074	880.3834	0.42	299163	13.43	2	DS=9	βCD((C3H6O))10Na2
881.1624	881.2415	-0.08	305001	13.69	2	DS=8+2NaCl	βCD((C3H6O))8(NaCl)2Na2
910.0806	909.9181	0.16	241730	10.85	2	DS=11	βCD((C3H6O))11Na2
938.7934	938.9577	-0.16	134933	6.06	2	DS=12	βCD((C3H6O))12Na2
939.1401	939.1393	0.00	54193	2.43	2	DS=11+NaCl	βCD((C3H6O))11(NaCl)1Na2
939.8323	939.8654	-0.03	69320	3.11	2	DS=7+5NaCl	βCD((C3H6O))7(NaCl)5Na2

Figure S26: ESIMS+ spectrum of HP-β-CD, prepared in ball mill, DS ≈ 4.4, entry 1 of Table S1

mMass Report: HPxCD_LJ02_Na(+)

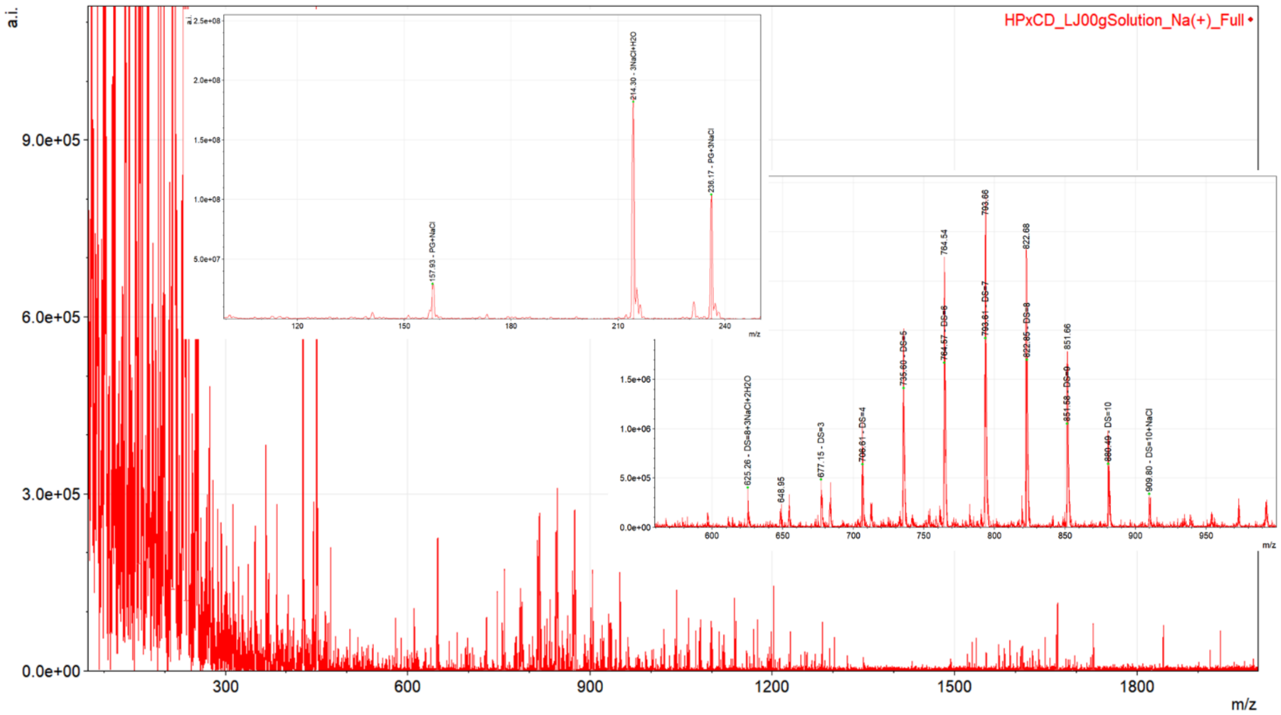

α

Annotations

Meas. m/z	Calc. m/z	δ (Da)	Int.	Rel. Int. (%)	z	Annotation	Formula
157.9333	157.5261	0.41	28928628	15.90	1	PG+NaCl	(HOC3H7O)1(NaCl)1Na
214.2978	214.8757	-0.58	181972693	100.00	1	3NaCl+H2O	(HOC3H7O)0(NaCl)3(H2O)1Na1
236.1670	236.3634	-0.20	104086399	57.20	2	PG+6NaCl	(HOC3H7O)1(NaCl)6Na2
625.2633	625.8564	-0.59	394072	20.62	3	DS=8+3NaCl+2H2O	βCD((C3H6O))8(NaCl)3Na3(H2O)2
677.1484	677.6011	-0.45	474508	24.83	2	DS=3	βCD((C3H6O))3Na2
706.6121	706.6407	-0.03	629808	32.96	2	DS=4	βCD((C3H6O))4Na2
735.6010	735.6803	-0.08	1403950	73.47	2	DS=5	βCD((C3H6O))5Na2
764.5748	764.7200	-0.15	1660653	86.90	2	DS=6	βCD((C3H6O))6Na2
793.6056	793.7596	-0.15	1911033	100.00	2	DS=7	βCD((C3H6O))7Na2
822.8531	822.7992	0.05	1691128	88.49	2	DS=8	βCD((C3H6O))8Na2
851.5765	851.8388	-0.26	1041009	54.47	2	DS=9	βCD((C3H6O))9Na2
880.4904	880.8785	-0.39	636383	33.30	2	DS=10	βCD((C3H6O))10Na2
909.8049	910.0996	-0.29	329071	17.22	2	DS=10+NaCl	βCD((C3H6O))10(NaCl)1Na2

Figure S27: ESIMS+ spectrum of HP-β-CD, prepared in ball mill, DS ≈ 5.6, entry 2 of Table S1

mMass Report: HPxCD_LJ03_Na(+)

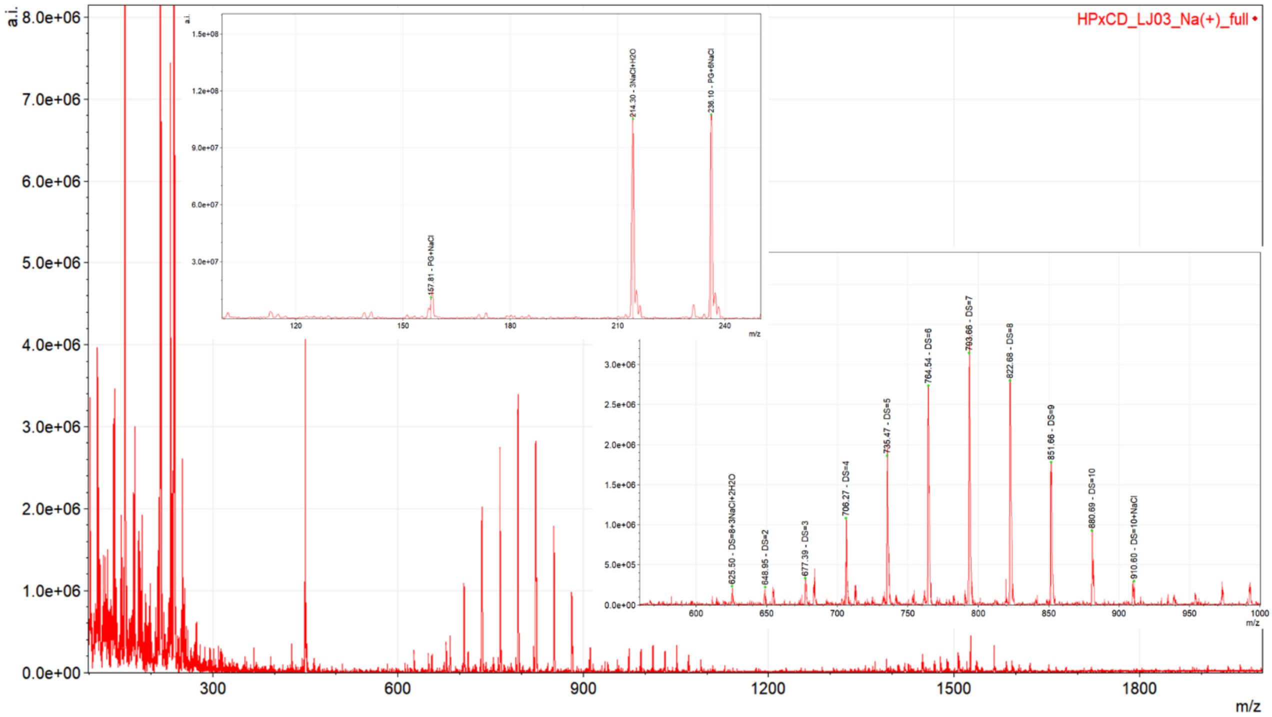

Annotations

Meas. m/z	Calc. m/z	δ (Da)	Int.	Rel. Int. (%)	z	Annotation	Formula
157.8145	157.5261	0.29	10820307	10.08	1	PG+NaCl	(HOC3H7O)1(NaCl)1Na
214.3047	214.8757	-0.57	105012705	97.83	1	3NaCl+H2O	(HOC3H7O)0(NaCl)3(H2O)1Na
236.0989	236.3634	-0.26	107338765	100.00	2	PG+6NaCl	(HOC3H7O)1(NaCl)6Na2
625.5008	625.8564	-0.36	227149	7.25	3	DS=8+3NaCl+2H2O	βCD((C3H6O))8(NaCl)3Na3(H2O)2
648.9451	648.5614	0.38	216735	6.91	2	DS=2	βCD((C3H6O))2Na2
677.3874	677.6011	-0.21	326080	10.40	2	DS=3	βCD((C3H6O))3Na2
706.2663	706.6407	-0.37	1072448	34.21	2	DS=4	βCD((C3H6O))4Na2
735.4736	735.6803	-0.21	1855827	59.20	2	DS=5	βCD((C3H6O))5Na2
764.5399	764.7200	-0.18	2730386	87.10	2	DS=6	βCD((C3H6O))6Na2
793.6562	793.7596	-0.10	3134950	100.00	2	DS=7	βCD((C3H6O))7Na2
822.6798	822.7992	-0.12	2792273	89.07	2	DS=8	βCD((C3H6O))8Na2
851.6629	851.8388	-0.18	1773895	56.58	2	DS=9	βCD((C3H6O))9Na2
880.6897	880.8785	-0.19	916957	29.25	2	DS=10	βCD((C3H6O))10Na2
910.6001	910.0996	0.50	287461	9.17	2	DS=10+NaCl	βCD((C3H6O))10(NaCl)1Na2

Figure S28: ESIMS+ spectrum of HP-β-CD, prepared in ball mill, DS ≈ 5.3, entry 3 of Table S1

mMass Report: HPxCD_LJ05_Na(+)

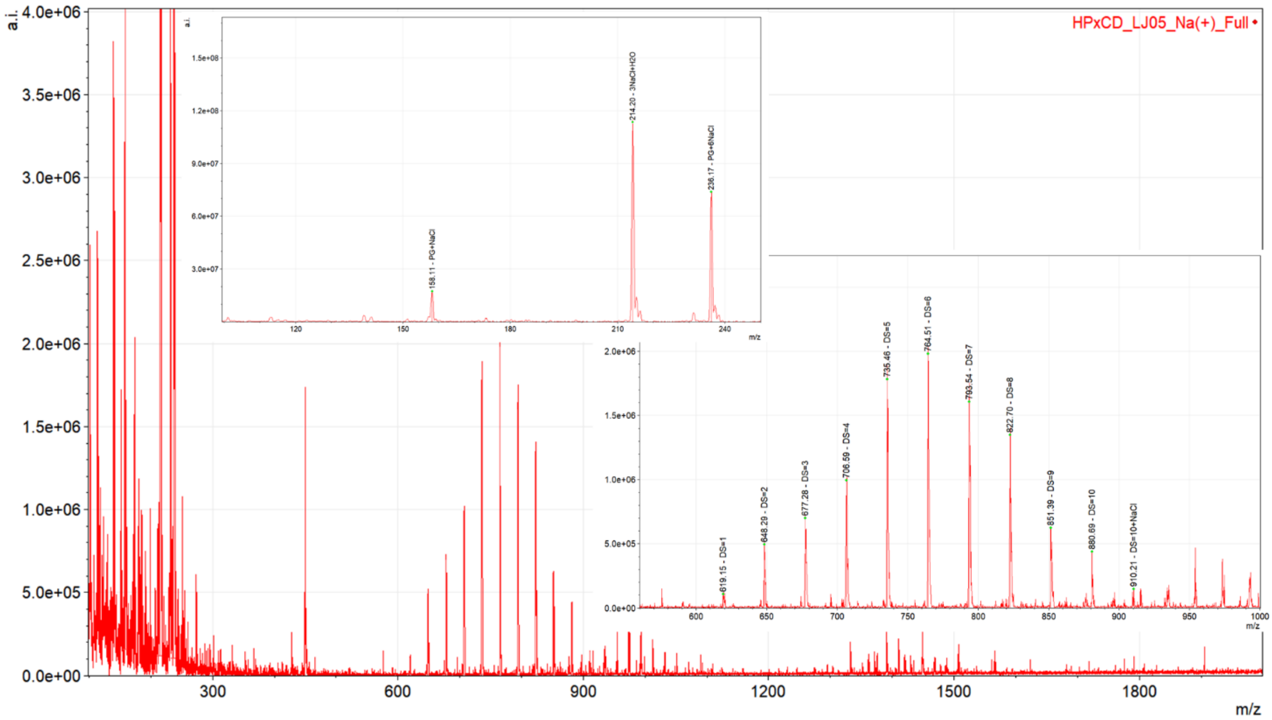

Annotations

Meas. m/z	Calc. m/z	δ (Da)	Int.	Rel. Int. (%)	z	Annotation	Formula
158.1063	157.5261	0.58	17191158	15.15	1	PG+NaCl	(HOC3H7O)1(NaCl)1Na
214.2026	214.8757	-0.67	113495239	100.00	1	3NaCl+H2O	(HOC3H7O)0(NaCl)3(H2O)1Na
236.1691	236.3634	-0.19	73937942	65.15	2	PG+6NaCl	(HOC3H7O)1(NaCl)6Na2
619.1461	619.1950	-0.05	102484	5.19	2	DS=1	βCD((C3H6O))1Na2
648.2908	648.5614	-0.27	489318	24.80	2	DS=2	βCD((C3H6O))2Na2
677.2753	677.6011	-0.33	692534	35.10	2	DS=3	βCD((C3H6O))3Na2
706.5949	706.6407	-0.05	987141	50.02	2	DS=4	βCD((C3H6O))4Na2
735.4606	735.6803	-0.22	1775787	89.99	2	DS=5	βCD((C3H6O))5Na2
764.5142	764.7200	-0.21	1973299	100.00	2	DS=6	βCD((C3H6O))6Na2
793.5373	793.7596	-0.22	1599230	81.04	2	DS=7	βCD((C3H6O))7Na2
822.7015	822.7992	-0.10	1342781	68.05	2	DS=8	βCD((C3H6O))8Na2
851.3854	851.8388	-0.45	615076	31.17	2	DS=9	βCD((C3H6O))9Na2
880.6901	880.8785	-0.19	431972	21.89	2	DS=10	βCD((C3H6O))10Na2
910.2080	910.0996	0.11	139377	7.06	2	DS=10+NaCl	βCD((C3H6O))10(NaCl)1Na2

Figure S29: ESIMS+ spectrum of HP-β-CD, prepared in ball mill, DS ≈ 3.7, entry 4 of Table S1

mMass Report: HPxCD_LJ00gSolution_Na(+)

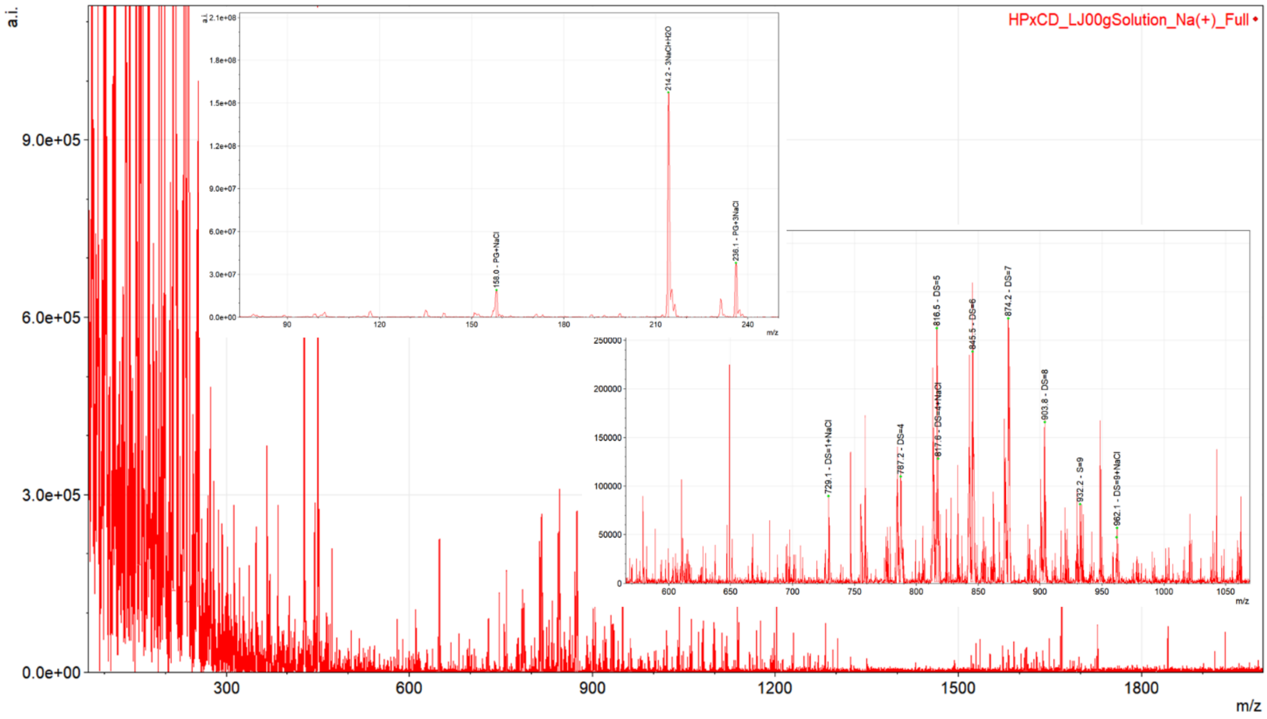

Annotations

Meas. m/z	Calc. m/z	δ (Da)	Int.	Rel. Int. (%)	z	Annotation	Formula
158.0422	157.5261	0.52	18818063	11.95	1	PG+NaCl	(HOC3H7O)1(NaCl)1Na
214.1530	214.8757	-0.72	157518790	100.00	1	3NaCl+H2O	(HOC3H7O)0(NaCl)3(H2O)Na
236.1220	236.3634	-0.24	37965920	24.10	2	PG+6NaCl	(HOC3H7O)1(NaCl)6Na2
729.0900	729.2008	-0.11	88606	32.66	2	DS=1+NaCl	γCD((C3H6O))1(NaCl)1Na2
787.2478	787.2842	-0.04	108668	40.06	2	DS=4	γCD((C3H6O))4Na2
816.5119	816.7508	-0.24	261415	96.36	2	DS=5	γCD((C3H6O))5Na2
817.6079	817.2714	0.34	126850	46.76	2	DS=4+NaCl	γCD((C3H6O))4(NaCl)1Na2
845.4774	845.7904	-0.31	237079	87.39	2	DS=6	γCD((C3H6O))6Na2
874.1817	874.3470	-0.17	271289	100.00	2	DS=7	γCD((C3H6O))7Na2
903.8482	903.8696	-0.02	164320	60.57	2	DS=8	γCD((C3H6O))8Na2
932.2436	932.3889	-0.15	80166	29.55	2	S=9	γCD((C3H6O))9Na2
961.8796	961.9489	-0.07	46042	16.97	2	DS=10	γCD((C3H6O))10Na2
962.1476	962.1304	0.02	55777	20.56	2	DS=9+NaCl	γCD((C3H6O))9(NaCl)1Na2

Figure S30: ESIMS+ spectrum of HP-γ-CD, prepared in solution, DS ≈ 4.5

mMass Report: HPxCD_LJ04_Na(+)

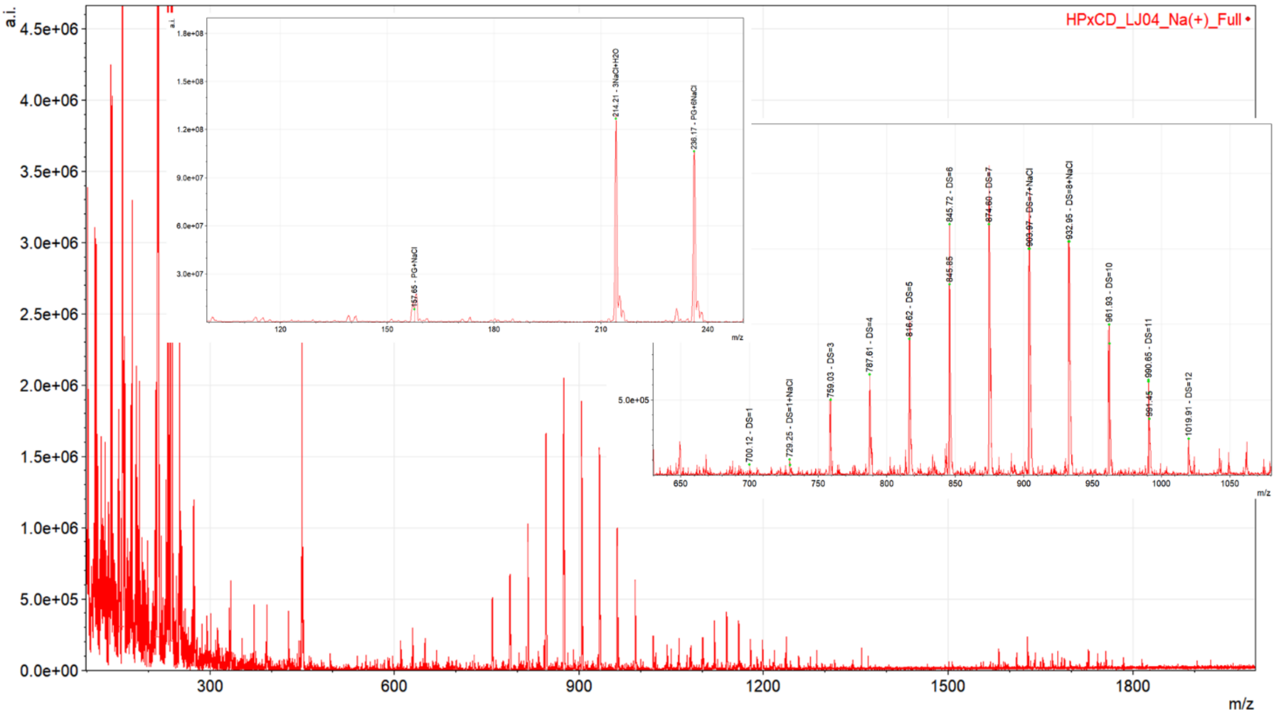

Annotations

Meas. m/z	Calc. m/z	δ (Da)	Int.	Rel. Int. (%)	z	Annotation	Formula
157.6543	157.5261	0.13	7777779	6.14	1	PG+NaCl	(HOC3H7O)1(NaCl)1Na
214.2137	214.8757	-0.66	126600013	100.00	1	3NaCl+H2O	(HOC3H7O)0(NaCl)3(H2O)1Na
236.1681	236.3634	-0.19	106251082	83.93	2	PG+6NaCl	(HOC3H7O)1(NaCl)6Na2
700.1152	700.2214	-0.11	63305	3.83	2	DS=1	γCD((C3H6O))1Na2
729.2460	729.2008	0.05	97918	5.92	2	DS=1+NaCl	γCD((C3H6O))1(NaCl)1Na2
729.6389	729.6319	0.01	59350	3.59	2	DS=2	γCD((C3H6O))2Na2
759.0225	758.8530	0.17	492656	29.78	2	DS=2+NaCl	γCD((C3H6O))2(NaCl)1Na2
759.0225	759.0345	-0.01	492656	29.78	2	DS=1+2NaCl	γCD((C3H6O))1(NaCl)2Na2
759.0290	759.2711	-0.24	492656	29.78	2	DS=3	γCD((C3H6O))3Na2
787.6110	787.7111	-0.10	659707	39.88	2	DS=4	γCD((C3H6O))4Na2
816.6161	816.7508	-0.13	894564	54.08	2	DS=5	γCD((C3H6O))5Na2
845.7237	845.7904	-0.07	1654158	100.00	2	DS=6	γCD((C3H6O))6Na2
845.8540	845.9719	-0.12	1256053	75.93	2	DS=5+NaCl	γCD((C3H6O))5(NaCl)1Na2
874.5962	874.8300	-0.23	1653547	99.96	2	DS=7	γCD((C3H6O))7Na2
903.5628	903.8696	-0.31	1491679	90.18	2	DS=8	γCD((C3H6O))8Na2
903.9663	904.0512	-0.08	1491679	90.18	2	DS=7+NaCl	γCD((C3H6O))7(NaCl)1Na2
932.4208	932.3889	0.03	1538132	92.99	2	DS=9	γCD((C3H6O))9Na2
932.9475	933.0908	-0.14	1538132	92.99	2	DS=8+NaCl	γCD((C3H6O))8(NaCl)1Na2
961.4027	961.4098	-0.01	953256	57.63	2	DS=10	γCD((C3H6O))10Na2
961.9307	961.9489	-0.02	989198	59.80	2	DS=10	γCD((C3H6O))10Na2
962.1992	962.1304	0.07	862505	52.14	2	DS=9+NaCl	γCD((C3H6O))9(NaCl)1Na2
990.6458	990.3891	0.26	611525	36.97	2	DS=10+NaCl	γCD((C3H6O))10(NaCl)1Na2
990.6541	990.3891	0.27	621600	37.58	2	DS=11	γCD((C3H6O))10(NaCl)1Na2
990.9192	991.1701	-0.25	534881	32.34	2	DS=10+NaCl	γCD((C3H6O))10(NaCl)1Na2
991.4468	991.3516	0.10	364099	22.01	2	DS=9+2NaCl	γCD((C3H6O))9(NaCl)2Na2
1019.9065	1020.2097	-0.30	231810	14.01	2	DS=11+NaCl	γCD((C3H6O))11(NaCl)1Na2
1019.9065	1020.0282	-0.12	231810	14.01	2	DS=12	γCD((C3H6O))12Na2

Figure S31: ESIMS+ spectrum of HP-γ-CD, prepared in ball mill, DS ≈ 5.1, entry 5 of Table S1

mMass Report: HPxCD_LJ06_Na(+)

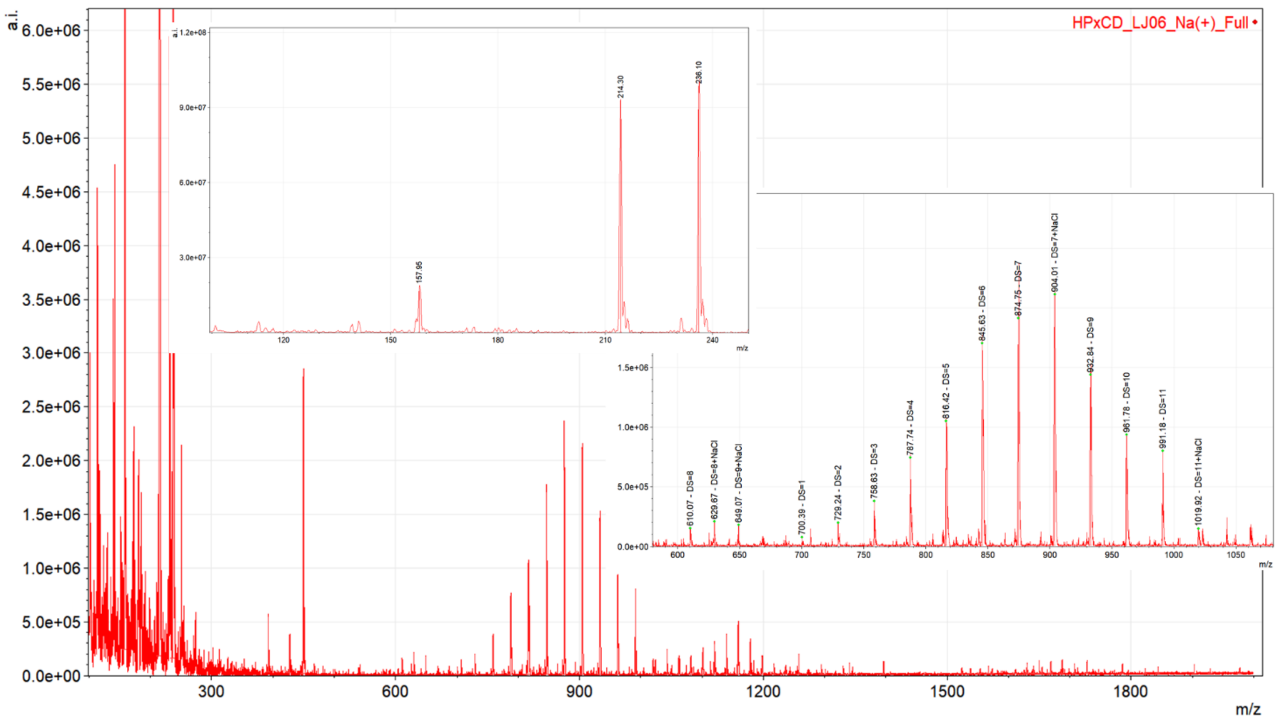

Annotations

Meas. m/z	Calc. m/z	δ (Da)	Int.	Rel. Int. (%)	z	Annotation	Formula
157.9481	157.5261	0.42	18429744	18.70	1	PG+NaCl	(HOC3H7O)1(NaCl)1Na
214.3031	214.8757	-0.57	92731767	94.09	1	3NaCl+H2O	(HOC3H7O)0(NaCl)3(H2O)1Na
236.0955	236.3634	-0.27	98557859	100.00	2	PG+6NaCL	(HOC3H7O)1(NaCl)6Na2
610.0685	610.3639	-0.30	145268	5.24	3	DS=7+NaCl	γCD((C3H6O))7(NaCl)1K0Na3
610.0685	610.2428	-0.17	145268	5.24	3	DS=8	γCD((C3H6O))8K0Na3
629.6651	629.7236	-0.06	202253	7.30	3	DS=8+NaCl	γCD((C3H6O))8(NaCl)1K0Na3
649.0694	649.0834	-0.01	172873	6.24	3	DS=9+NaCl	γCD((C3H6O))9(NaCl)1K0Na3
700.3863	700.2214	0.16	71869	2.59	2	DS=1	γCD((C3H6O))1K0Na2
729.2434	729.2424	0.00	193087	6.97	2	DS=2	γCD((C3H6O))2K0Na2
758.6272	758.6715	-0.04	372330	13.44	2	DS=3	γCD((C3H6O))3K0Na2
787.7376	787.7111	0.03	738249	26.65	2	DS=4	γCD((C3H6O))4K0Na2
816.4240	816.3052	0.12	1043114	37.66	2	DS=5	γCD((C3H6O))5K0Na2
845.6274	845.7904	-0.16	1696576	61.25	2	DS=6	γCD((C3H6O))6K0Na2
874.7544	874.8300	-0.08	1905663	68.80	2	DS=7	γCD((C3H6O))7K0Na2
904.0054	903.8696	0.14	2105242	76.00	2	DS=7+NaCl	γCD((C3H6O))8K0Na2
932.8383	932.9093	-0.07	1430665	51.65	2	DS=9	γCD((C3H6O))9K0Na2
961.7763	961.9489	-0.17	928360	33.52	2	DS=10	γCD((C3H6O))10K0Na2
991.1842	991.1701	0.01	793795	28.66	2	DS=11	γCD((C3H6O))10(NaCl)1K0Na2
1019.9153	1020.2097	-0.29	138110	4.99	2	DS12	γCD((C3H6O))11(NaCl)1Na2
1019.9153	1020.0282	-0.11	138110	4.99	2	DS=11+NaCl	γCD((C3H6O))12Na2

Figure S32: ESIMS+ spectrum of HP-γ-CD, prepared in ball mill, DS ≈ 2.3-2.6, entry 6 of Table S1

mMass Report: HPxCD_LJ_19A_Na(+)

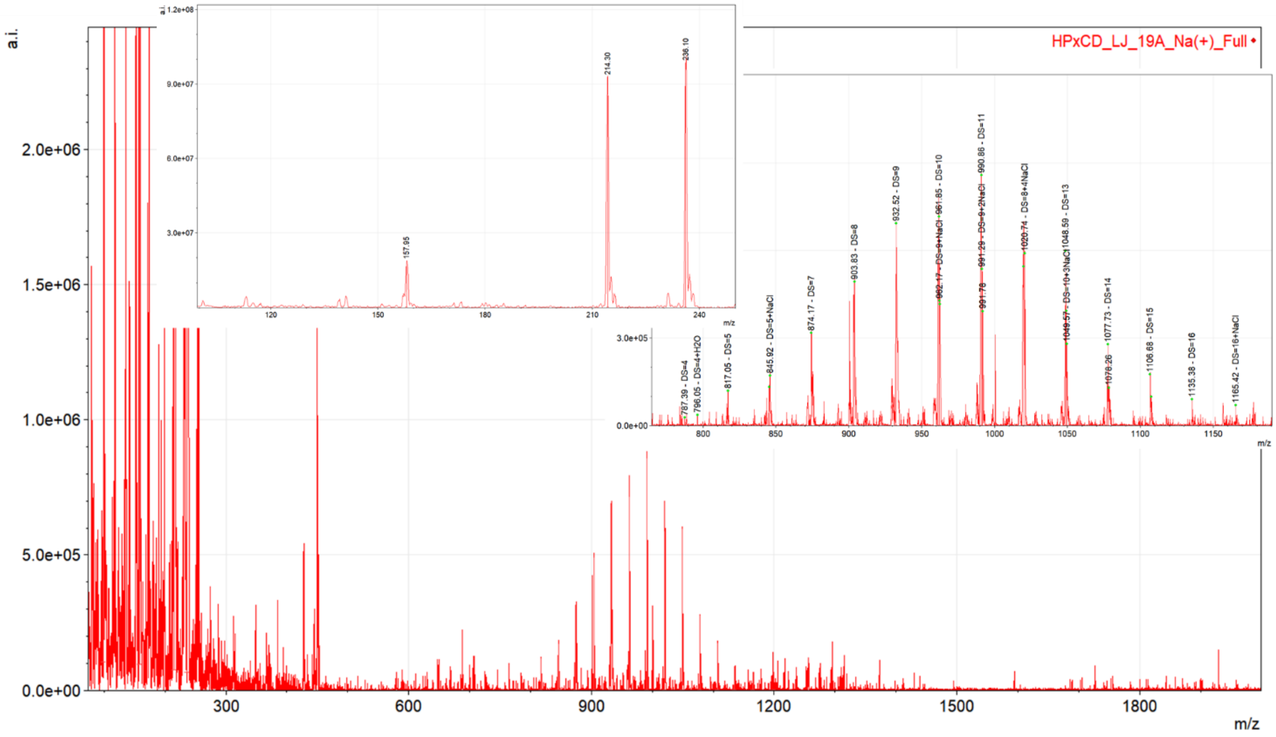

Annotations

Meas. m/z	Calc. m/z	δ (Da)	Int.	Rel. Int. (%)	z	Annotation	Formula
98.8757	99.0417	-0.17	2550262	2.99	1	PG	(HOC3H7O)1Na
157.5508	157.5261	0.02	6239618	7.31	1	PG+NacL	(HOC3H7O)1(NaCl)1Na
214.3169	214.8757	-0.56	85380316	100.00	1	3NaCl+H2O	(HOC3H7O)0(NaCl)3(H2O)1Na1
236.1148	236.3634	-0.25	47422778	55.54	2	PG+6NaCl	(HOC3H7O)1(NaCl)6Na2
787.3877	787.2842	0.10	26350	3.07	2	DS=4	γCD((C3H6O))4Na2
796.0466	796.2895	-0.24	34977	4.08	2	DS=4+H2O	γCD((C3H6O))4(H2O)Na2
817.0540	816.7508	0.30	117669	13.72	2	DS=5	γCD((C3H6O))5Na2
845.3076	845.3261	-0.02	131115	15.28	2	DS=6	γCD((C3H6O))6Na2
845.9204	845.9719	-0.05	170817	19.91	2	DS=5+NaCl	γCD((C3H6O))5(NaCl)1Na2
874.1707	874.3470	-0.18	316827	36.93	2	DS=7	γCD((C3H6O))7Na2
903.8309	903.8696	-0.04	492181	57.37	2	DS=8	γCD((C3H6O))8Na2
932.5170	932.3889	0.13	692288	80.70	2	DS=9	γCD((C3H6O))9Na2
961.8548	961.9489	-0.09	715236	83.38	2	DS=10	γCD((C3H6O))10Na2
962.1653	962.1304	0.03	426353	49.70	2	DS=9+NaCl	γCD((C3H6O))9(NaCl)1Na2
962.3852	962.3119	0.07	414394	48.31	2	DS=8+2NaCl	γCD((C3H6O))8(NaCl)2Na2
990.8650	990.9885	-0.12	857833	100.00	2	DS=11	γCD((C3H6O))11Na2
991.2923	991.3516	-0.06	535545	62.43	2	DS=9+2NaCl	γCD((C3H6O))9(NaCl)2Na2
991.7849	991.7146	0.07	390839	45.56	2	DS=7+4NaCl	γCD((C3H6O))7(NaCl)4Na2
1019.8180	1020.0282	-0.21	543556	63.36	2	DS=12	γCD((C3H6O))12Na2
1020.7439	1020.7543	-0.01	589476	68.72	2	DS=8+4NaCl	γCD((C3H6O))8(NaCl)4Na2
1048.5929	1048.4726	0.12	597552	69.66	2	DS=13	γCD((C3H6O))13Na2
1049.1531	1049.2493	-0.10	392309	45.73	2	DS=12+NaCl	γCD((C3H6O))12(NaCl)1Na2
1049.5679	1049.6124	-0.04	278137	32.42	2	DS=10+3NaCl	γCD((C3H6O))10(NaCl)3Na2
1077.7306	1077.4936	0.24	276695	32.26	2	DS=14	γCD((C3H6O))14Na2
1078.2637	1078.2889	-0.03	128397	14.97	2	DS713+NaCl	γCD((C3H6O))13(NaCl)1Na2
1106.6772	1106.5145	0.16	173814	20.26	2	DS=15	γCD((C3H6O))15Na2
1107.3660	1107.3286	0.04	97765	11.40	2	DS714+NaCl	γCD((C3H6O))14(NaCl)1Na2
1135.3800	1135.5354	-0.16	89165	10.39	2	DS=16	γCD((C3H6O))16Na2
1165.4189	1165.4078	0.01	67713	7.89	2	DS=16+NaCl	γCD((C3H6O))16(NaCl)1Na2

Figure S33: ESIMS+ spectrum of HP-γ-CD, prepared in solution, DS ≈ 8.8, entry 7 of Table S1

mMass Report: HPxCD_LJ_18_Na(+)

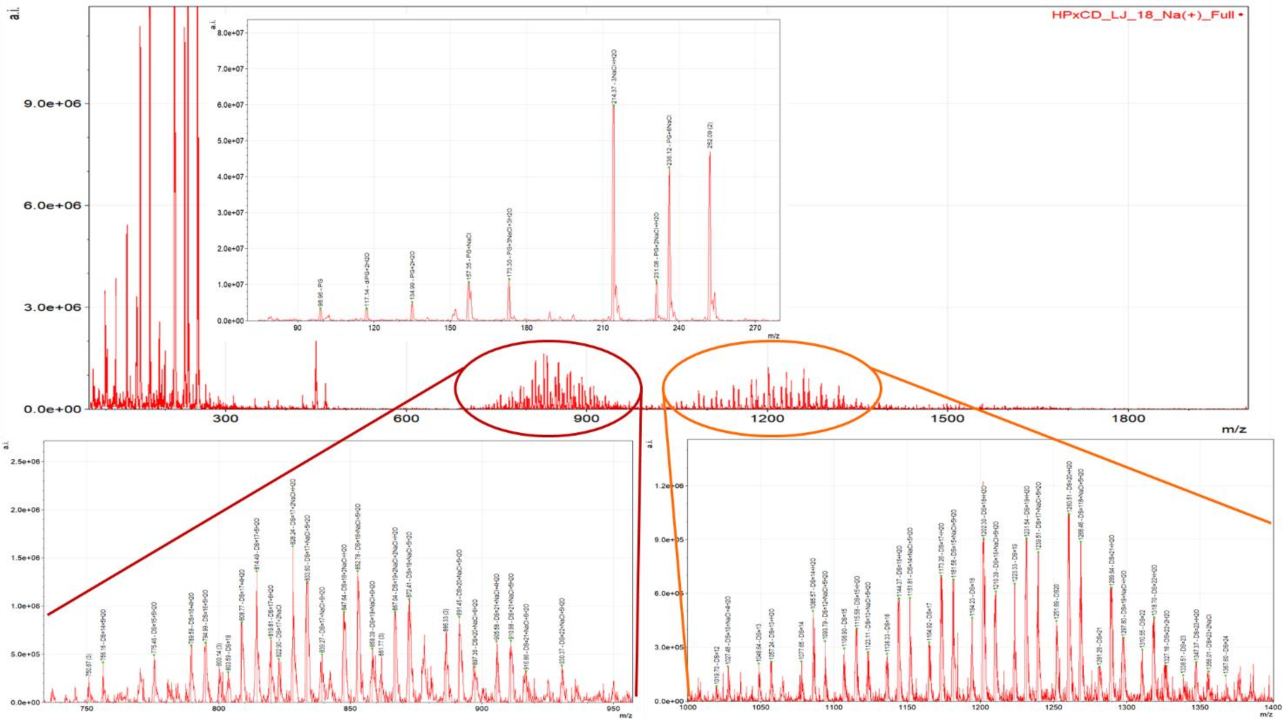

Annotations

Meas. m/z	Calc. m/z	δ (Da)	Int.	Rel. Int. (%)	z	Annotation	Formula
98.9559	99.0838	-0.13	3301857	5.53	1	PG	(HOC3H7O)1Na
117.1431	117.0522	0.09	3251338	5.44	2	diPG+2H2O	(HOC3H7O)2(H2O)2Na2
134.9887	135.0628	-0.07	5092347	8.52	1	PG+2H2O	(HOC3H7O)1(H2O)2Na
157.3464	157.5261	-0.18	10573582	17.70	1	PG+NaCl	(HOC3H7O)1(NaCl)1Na1
173.2961	173.9614	-0.67	11457590	19.18	2	PG+3NaCl+3H2O	(HOCH2CHOHCH)1(NaCl)3(H2O)3Na2
214.3746	214.8757	-0.50	59737319	100.00	1	3NaCl+H2O	(HOC3H7O)0(NaCl)3(H2O)1Na1
231.0808	230.9538	0.13	11195004	18.74	1	PG+2NaCl+H2O	(HOCH2CHOHCH)1(NaCl)2(H2O)1Na1
236.1154	236.3634	-0.25	42587610	71.29	2	PG+6NaCl	(HOC3H7O)1(NaCl)6Na2
252.0905	251.8940	0.15	406223	25.27	3	DS=14+5H2O	γCD((C3H6O))14(H2O)5Na3
756.1600	756.0097	0.10	478721	29.78	3	DS=15+5H2O	γCD((C3H6O))15(H2O)5Na3
775.4579	775.3570	0.44	577957	35.96	3	DS=16+4H2O	γCD((C3H6O))16(H2O)4Na3
789.5858	789.1412	-0.16	606139	37.71	3	DS=16+5H2O	γCD((C3H6O))16(H2O)5Na3
794.9851	795.1463	-0.26	301850	18.78	3	DS=18	γCD((C3H6O))18Na3
803.5851	803.8404	0.27	817703	50.87	3	DS=17+4H2O	γCD((C3H6O))17(H2O)4Na3
808.7665	808.5010	-0.02	1348571	83.90	3	DS=17+5H2O	γCD((C3H6O))17(H2O)5Na3
814.4867	814.5061	-0.25	652902	40.62	3	DS=17+6H2O	γCD((C3H6O))17(H2O)6Na3
819.8085	820.0551	0.23	438983	27.31	3	DS=17+2NaCl	γCD((C3H6O))17(NaCl)2Na3
822.9030	822.6731	-0.44	1607390	100.00	3	DS=17+2NaCl+H2O	γCD((C3H6O))17(NaCl)2(H2O)1Na3
828.2414	828.6766	0.23	1229594	76.50	3	DS=17+NaCl+5H2O	γCD((C3H6O))17(NaCl)1(H2O)5Na3
833.6012	833.3711	-0.11	483769	30.10	3	DS=17+NaCl+6H2O	γCD((C3H6O))17(NaCl)1(H2O)6Na3
839.2686	839.3747	-0.38	925201	57.56	3	DS=18+2NaCl+H2O	γCD((C3H6O))18(NaCl)2(H2O)1Na3
847.6427	848.0239	0.06	1346012	83.74	3	DS=18+NaCl+5H2O	γCD((C3H6O))18(NaCl)1(H2O)5Na3
852.7832	852.7184	-0.33	546441	34.00	3	DS=18+NaCl+6H2O	γCD((C3H6O))18(NaCl)1(H2O)6Na3
858.3893	858.7219	-0.33	921253	57.31	3	DS=19+2NaCl+2NaCl+H2O	γCD((C3H6O))19(NaCl)2(H2O)1Na3
867.0439	867.3712	-0.30	1055192	65.65	3	DS=19+NaCl+5H2O	γCD((C3H6O))19(NaCl)1(H2O)5Na3
872.4056	872.7064	0.04	873900	54.37	3	DS=20+NaCl+5H2O	γCD((C3H6O))20(NaCl)1(H2O)5Na3

Meas. m/z	Calc. m/z	δ (Da)	Int.	Rel. Int. (%)	z	Annotation	Formula
891.4535	891.4130	-0.06	355883	22.14	3	DS=20+NaCl+6H2O	γCD((C3H6O))20(NaCl)1(H2O)6Na3
897.3554	897.4165	0.16	622526	38.73	3	DS=21+NaCl+4H2O	γCD((C3H6O))21(NaCl)1(H2O)4Na3
905.5823	905.4208	0.22	624566	38.86	3	DS=21+NaCl+5H2O	γCD((C3H6O))21(NaCl)1(H2O)5Na3
910.9818	910.7603	0.10	305379	19.00	3	DS=21+NaCl+6H2O	γCD((C3H6O))21(NaCl)1(H2O)6Na3
916.8607	916.7638	0.27	368258	22.91	3	DS=22+NaCl+5H2O	γCD((C3H6O))22(NaCl)1(H2O)5Na3
930.3748	930.1076	-0.33	70677	4.40	2	DS=12	γCD((C3H6O))12Na2
1019.6985	1020.0282	0.28	181904	11.32	2	DS=10+NaCl+4H2O	γCD((C3H6O))10(NaCl)1(H2O)4Na2
1027.4834	1027.2006	0.17	198707	12.36	2	DS=13	γCD((C3H6O))13Na2
1048.6396	1048.4726	-0.24	209016	13.00	2	DS=13+H2O	γCD((C3H6O))13(H2O)1Na2
1057.2350	1057.4779	0.16	212051	13.19	2	DS=14	γCD((C3H6O))14Na2
1077.6549	1077.4936	-0.92	493236	30.69	2	DS=14+H2O	γCD((C3H6O))14(H2O)1Na2
1085.5742	1086.4989	-0.50	323650	20.14	2	DS=12+NaCl+5H2O	γCD((C3H6O))12(NaCl)1(H2O)5Na2
1093.7881	1094.2875	0.38	283411	17.63	2	DS=15	γCD((C3H6O))15Na2
1106.8959	1106.5145	0.06	392591	24.42	2	DS=15+H2O	γCD((C3H6O))15(H2O)1Na2
1115.5834	1115.5198	-0.22	263872	16.42	2	DS=13+NaCl+5H2O	γCD((C3H6O))13(NaCl)1(H2O)5Na2
1123.1075	1123.3272	0.14	254316	15.82	2	DS=16	γCD((C3H6O))16Na2
1136.3293	1136.1867	-0.17	562700	35.01	2	DS=16+H2O	γCD((C3H6O))16(H2O)1Na2
1144.3735	1144.5407	0.31	566152	35.22	2	DS=14+NaCl+5H2O	γCD((C3H6O))14(NaCl)1(H2O)5Na2
1151.8103	1151.4993	-0.30	317659	19.76	2	DS=17	γCD((C3H6O))17Na2
1164.9232	1165.2263	-0.30	689614	42.90	2	DS=17+H2O	γCD((C3H6O))17(H2O)1Na2
1173.2573	1173.5616	0.18	668345	41.58	2	DS=15+NaCl+5H2O	γCD((C3H6O))15(NaCl)1(H2O)5Na2
1181.5849	1181.4064	-0.04	455692	28.35	2	DS=18	γCD((C3H6O))18Na2
1194.2276	1194.2659	-0.29	897849	55.86	2	DS=18+H2O	γCD((C3H6O))18(H2O)1Na2
1202.2972	1202.5826	-0.06	601406	37.42	2	DS=16+NaCl+5H2O	γCD((C3H6O))16(NaCl)1(H2O)5Na2
1210.3891	1210.4460	0.03	645909	40.18	2	DS=19	γCD((C3H6O))19Na2
1223.3338	1223.3056	-0.07	897544	55.84	2	DS=19+H2O	γCD((C3H6O))19(H2O)1Na2
1231.5365	1231.6035	0.02	822986	51.20	2	DS=17+NaCl+5H2O	γCD((C3H6O))17(NaCl)1(H2O)5Na2
1239.5057	1239.4857	0.27	438744	27.30	2	DS20	γCD((C3H6O))20Na2
1251.8938	1251.6192	-0.12	1035721	64.43	2	DS=20+H2O	γCD((C3H6O))20(H2O)1Na2
1260.5064	1260.6244	-0.06	881210	54.82	2	DS=118+NaCl+5H2O	γCD((C3H6O))18(NaCl)1(H2O)5Na2
1268.4650	1268.5253	-0.11	183075	11.39	2	DS=21	γCD((C3H6O))21Na2
1281.2764	1281.3848	-0.00	625041	38.89	2	DS=21+H2O	γCD((C3H6O))21(H2O)1Na2
1289.6423	1289.6454	0.24	345156	21.47	2	DS=19+NaCl+H2O	γCD((C3H6O))19(NaCl)1(H2O)5Na2
1297.8027	1297.5649	0.12	279267	17.37	2	DS=22	γCD((C3H6O))22Na2
1310.5477	1310.4244	0.04	459385	28.58	2	DS=22+H2O	γCD((C3H6O))22(H2O)1Na2
1318.7035	1318.6663	-0.49	188576	11.73	2	DS=22+2H2O	γCD((C3H6O))22(H2O)2Na2
1327.1843	1327.6716	-0.17	138263	8.60	2	DS=23	γCD((C3H6O))23Na2
1338.5118	1338.6820	-0.31	209497	13.03	2	DS=23+H2O	γCD((C3H6O))23(H2O)1Na2
1347.3728	1347.6872	-0.68	159799	9.94	2	DS=23+2NaCl	γCD((C3H6O))23(H2O)2Na2
1356.0092	1356.6925	-0.10	135441	8.43	2	DS=24	γCD((C3H6O))24Na2

Figure S34: ESIMS+ spectrum of HP-γ-CD, prepared in solution, DS ≈ 17.6, entry 8 of Table S1

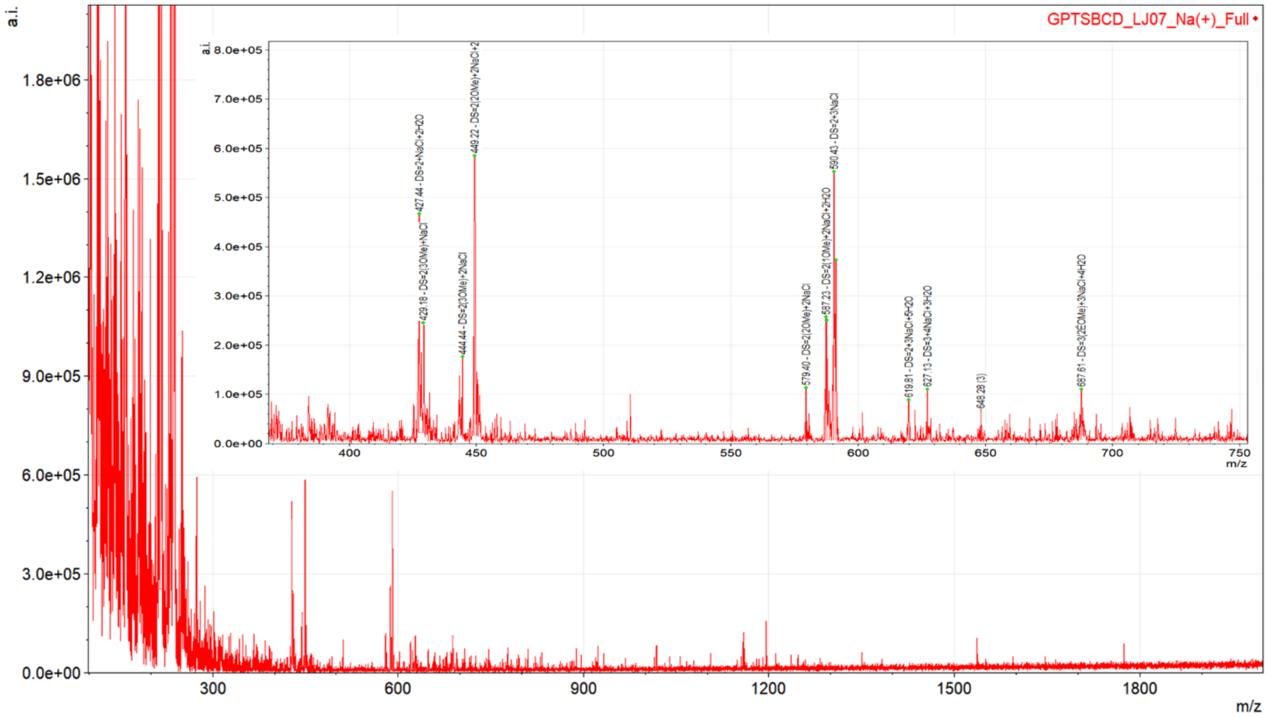

Annotations

Meas. m/z	Calc. m/z	δ (Da)	Int.	Rel. Int. (%)	z	Annotation	Formula
214.2112	214.8757	-0.66	159888174	--	1	3NaCl+H2O	(NaCl)3(H2O)1Na1
427.4405	427.6110	-0.17	444238	78.15	4	DS=2+NaCl+2H2O	βCD((C3H6O)O(CH2)3Si(OH)3)2(NaCl)1(H2O)2Na4
429.1763	429.1175	0.06	223137	39.26	4	DS=2(3OMe)+NaCl	βCD((C3H6O)O(CH2)3Si(OH)3)2(NaCl)1Na4(CH2)3
444.4377	444.1098	0.33	157582	27.72	4	DS=2(3OMe)+2NaCl	βCD((C3H6O)O(CH2)3Si(OH)3)2(NaCl)2Na4(CH2)3
449.2214	449.1085	0.11	568415	100.00	4	DS=2(2OMe)+2NaCl+2H2O	βCD((C3H6O)O(CH2)3Si(OH)3)2(NaCl)2(H2O)2Na4(CH2)2
579.3967	579.1412	0.26	105175	18.50	3	DS=2(2OMe)+2NaCl	βCD((C3H6O)O(CH2)3Si(OH)3)2(NaCl)2Na3(CH2)2
587.2319	587.1424	0.09	249028	43.81	3	DS=2(1OMe)+2NaCl+2H2O	βCD((C3H6O)O(CH2)3Si(OH)3)2(NaCl)2(H2O)2Na3(CH2)1
587.6319	587.8080	-0.18	242412	42.65	3	DS=2+2NaCl+3H2O	βCD((C3H6O)O(CH2)3Si(OH)3)2(NaCl)2(H2O)3Na3
590.4259	589.9375	0.49	544308	95.76	3	DS=2+3NaCl	βCD((C3H6O)O(CH2)3Si(OH)3)2(NaCl)3Na3
591.2254	591.1483	0.08	365672	64.33	3	DS=2(2OMe)+2NaCl+2H2O	βCD((C3H6O)O(CH2)3Si(OH)3)2(NaCl)2(H2O)2Na3(CH2)2
619.8051	619.9630	-0.16	80998	14.25	3	DS=2+3NaCl+5H2O	βCD((C3H6O)O(CH2)3Si(OH)3)2(NaCl)3(H2O)5Na3
627.1267	627.4335	-0.31	102753	18.08	3	DS=3+4NaCl+3H2O	βCD((C3H6O)O(CH2)3Si(OH)3)2(NaCl)4(H2O)3Na3
687.6131	687.4978	0.12	101702	17.89	3	DS=3(2ÉOMe)+3NaCl+4H2O	βCD((C3H6O)O(CH2)3Si(OH)3)3(NaCl)3(H2O)4Na3(CH2)2
1017.9092	1018.2810	-0.37	72577	12.77	2	DS=3+4NaCl+2H2O	βCD((C3H6O)O(CH2)3Si(OH)3)3(NaCl)4(H2O)2Na2

Figure S35: ESIMS+ spectrum of GPTS-β-CD, prepared in solution, DS ≈ 2.3-2.6, entry 15 of Table S1

mMass

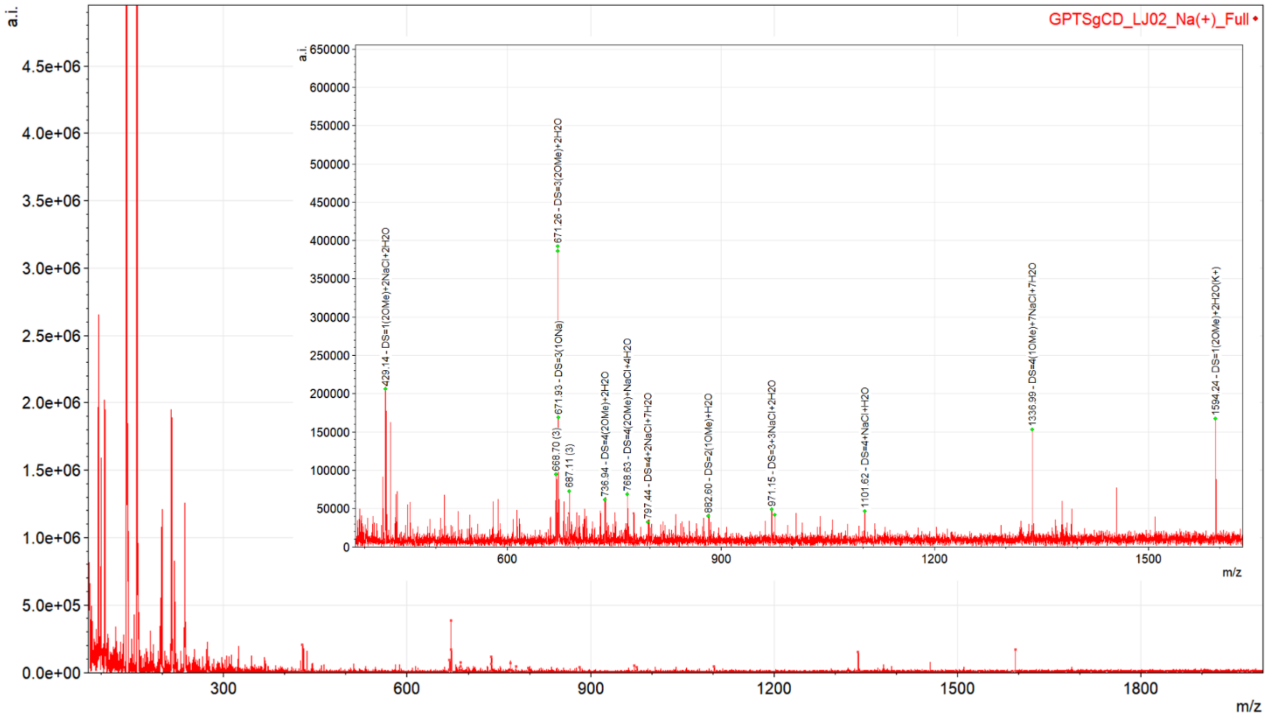

Annotations

Meas. m/z	Calc. m/z	δ (Da)	Int.	Rel. Int. (%)	z	Annotation	Formula
429.1416	429.3596	-0.22	197333	51.21	**4**	DS=1(2OMe)+2NaCl+2H2O	γCD((C3H6O)O(CH2)3Si(OH)3)1(NaCl)2(H2O)2Na2(CH2)2
668.7011	668.6461	0.06	87433	22.69	**3**	DS=3+3H2O	γCD((C3H6O)O(CH2)3Si(OH)3)3(H2O)3Na3
671.1221	671.0977	0.02	379176	98.39	**3**	DS=2+NaCl+GPTS	γCD((C3H6O)O(CH2)3Si(OH)3)1(NaCl)1Na4C12H26O9Si2
671.2575	671.5498	-0.29	385365	100.00	**3**	DS=3(2OMe)+2H2O	γCD((C3H6O)O(CH2)3Si(OH)3)3(H2O)2Na3(CH2)2
671.9297	672.1809	-0.25	161798	41.99	**3**	DS=3(1ONa)	γCD((C3H6O)O(CH2)3Si(OH)2ONa)3Na3
687.1122	687.3482	-0.24	65575	17.02	**3**	DS=3(4OMe)+3H2O	γCD((C3H6O)O(CH2)3Si(OH)3)3(H2O)3Na3(CH2)4
736.9423	737.0807	-0.14	54221	14.07	**3**	DS=4(2OMe)+2H2O	γCD((C3H6O)O(CH2)3Si(OH)3)4(H2O)2Na3(CH2)2
768.6318	768.5717	0.06	61777	16.03	**3**	DS=4(2OMe)+NaCl+4H2O	γCD((C3H6O)O(CH2)3Si(OH)3)4(NaCl)1(H2O)4Na3(CH2)2
797.4376	797.7246	-0.29	24728	6.42	**3**	DS=4+2NaCl+7H2O	γCD((C3H6O)O(CH2)3Si(OH)3)4(NaCl)2(H2O)7Na3
882.6021	882.8395	-0.24	32587	8.46	**2**	DS=2(1OMe)+H2O	γCD((C3H6O)O(CH2)3Si(OH)3)2(H2O)1Na2(CH2)1
971.1550	971.2179	-0.06	41055	10.65	**2**	DS=3+3NaCl+2H2O	γCD((C3H6O)O(CH2)3Si(OH)3)2(NaCl)3(H2O)2Na2
975.7309	975.5220	0.21	34013	8.83	**2**	DS=2(3OMe)+3NaCl	γCD((C3H6O)O(CH2)3Si(OH)3)2(NaCl)3Na2(CH2)3
1101.6198	1101.3213	0.30	38545	10.00	**2**	DS=4+NaCl+H2O	γCD((C3H6O)O(CH2)3Si(OH)3)4(NaCl)1(H2O)1Na2
1336.9925	1336.6994	0.29	144327	37.45	**2**	DS=4(1OMe)+7NaCl+7H2O	γCD((C3H6O)O(CH2)3Si(OH)3)4(NaCl)7(H2O)7Na2(CH2)1
1594.2360	1594.5070	-0.27	157920	40.98	**1**	DS=1(2OMe)+2H2O(K+)	γCD((C3H6O)O(CH2)3Si(OH)3)1(H2O)2K(CH2)2

Figure S36: ESIMS+ spectrum of GPTS-γ-CD, prepared in solution, DS ≈ 2.3-2.6, entry 17 of Table S1

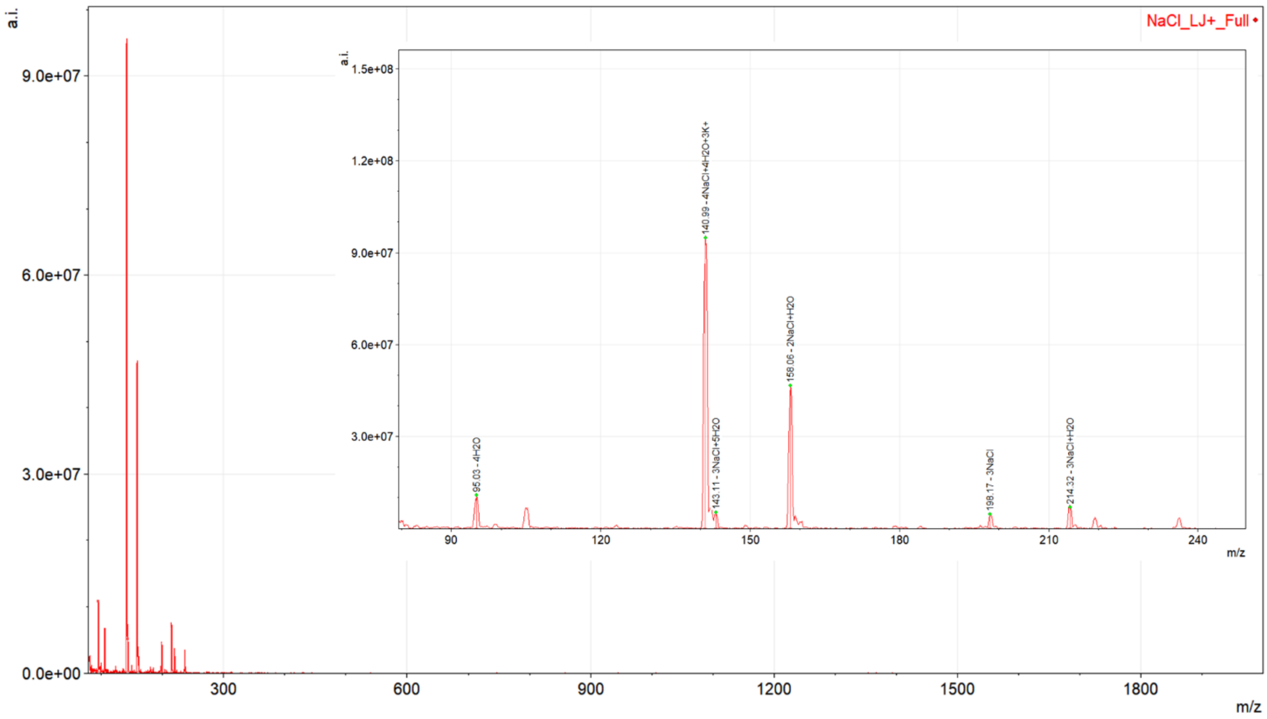

Figure S37: ESIMS+ spectrum of background (10 mM NaCl)

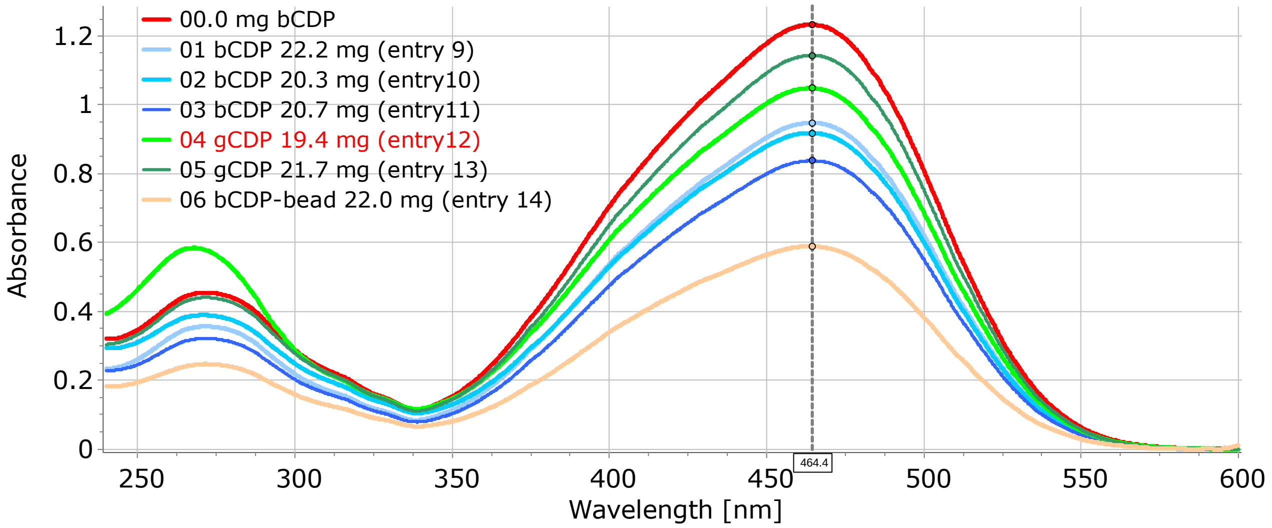

Figure S38: UV–vis spectra of CDPs after 1 day equilibration of ≈20 mg CDP and 0.05 mM methyl orange solution*

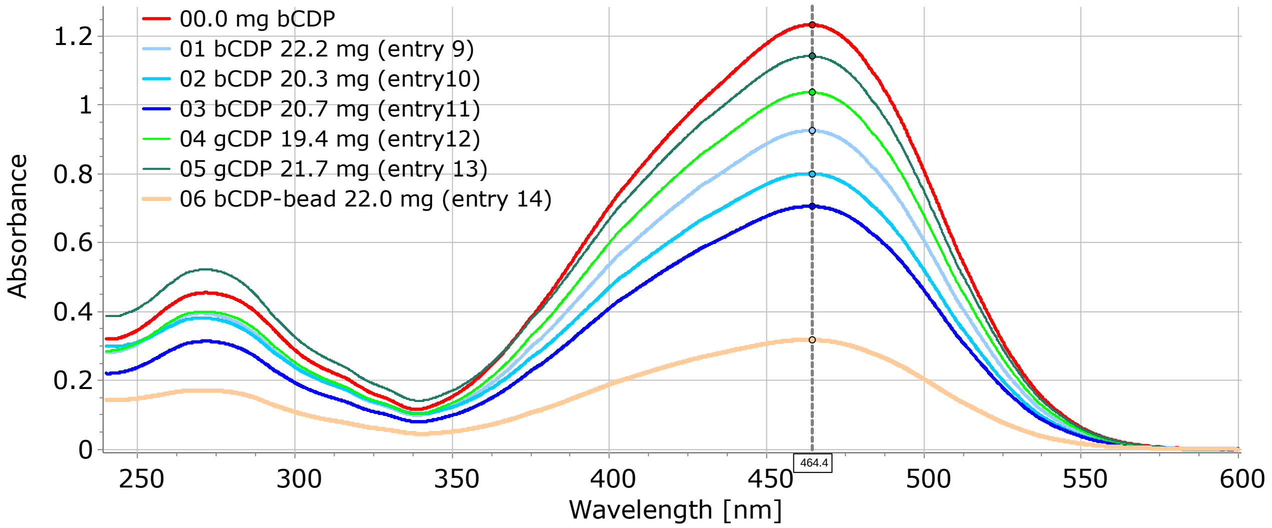

Figure S39: UV–vis spectra of CDPs after 2 weeks equilibration of ≈20 mg CDP and 0.05 mM methyl orange solution*

*Entry labels are corresponding to the body text, not the SI.

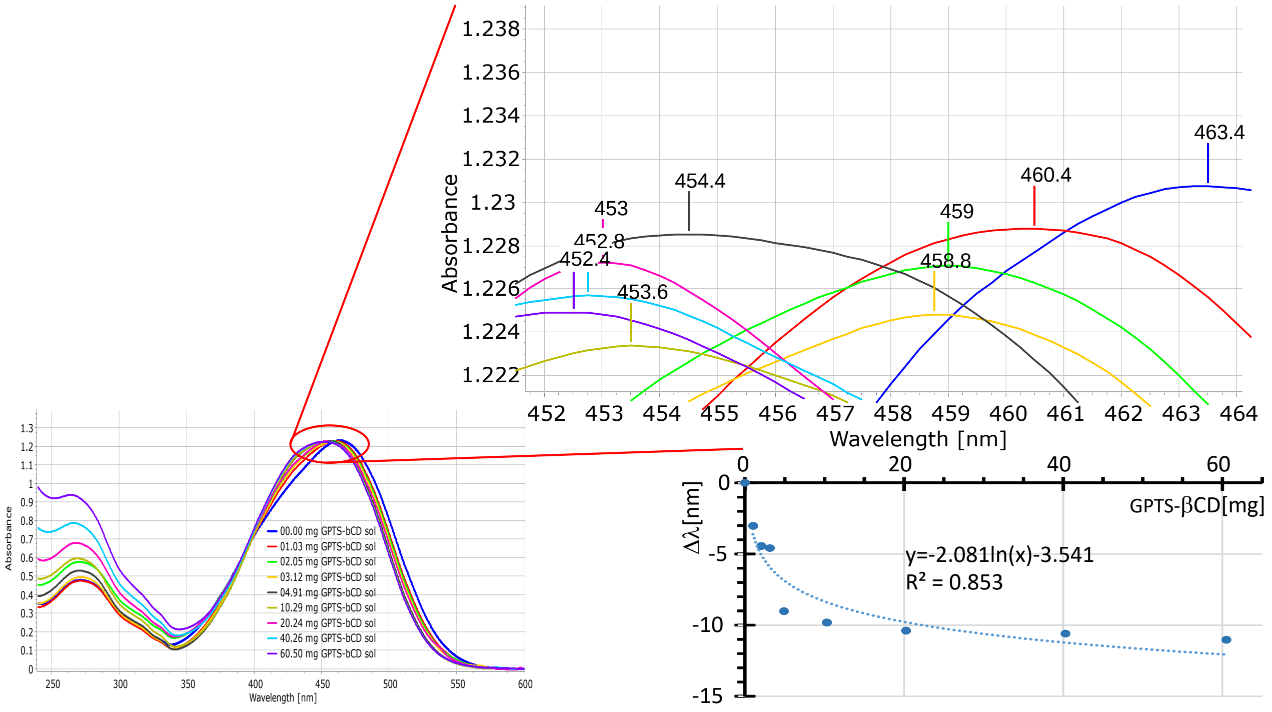

Figure S40: Soluble GPTS-β-CD (entry 15 Table 5) caused blueshifts in 0.05 mM methyl orange solution*

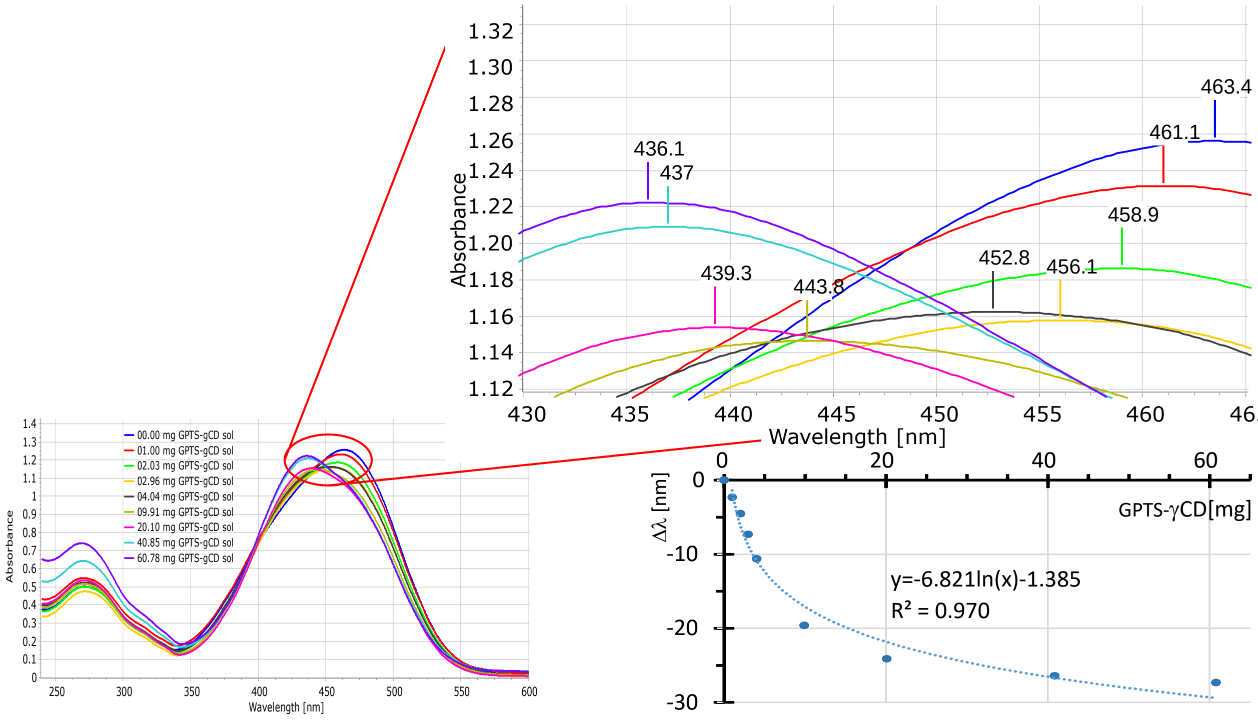

Figure S41: Soluble GPTS-γ-CD (entry 16 Table 5) caused blueshifts in 0.05 mM methyl orange solution*

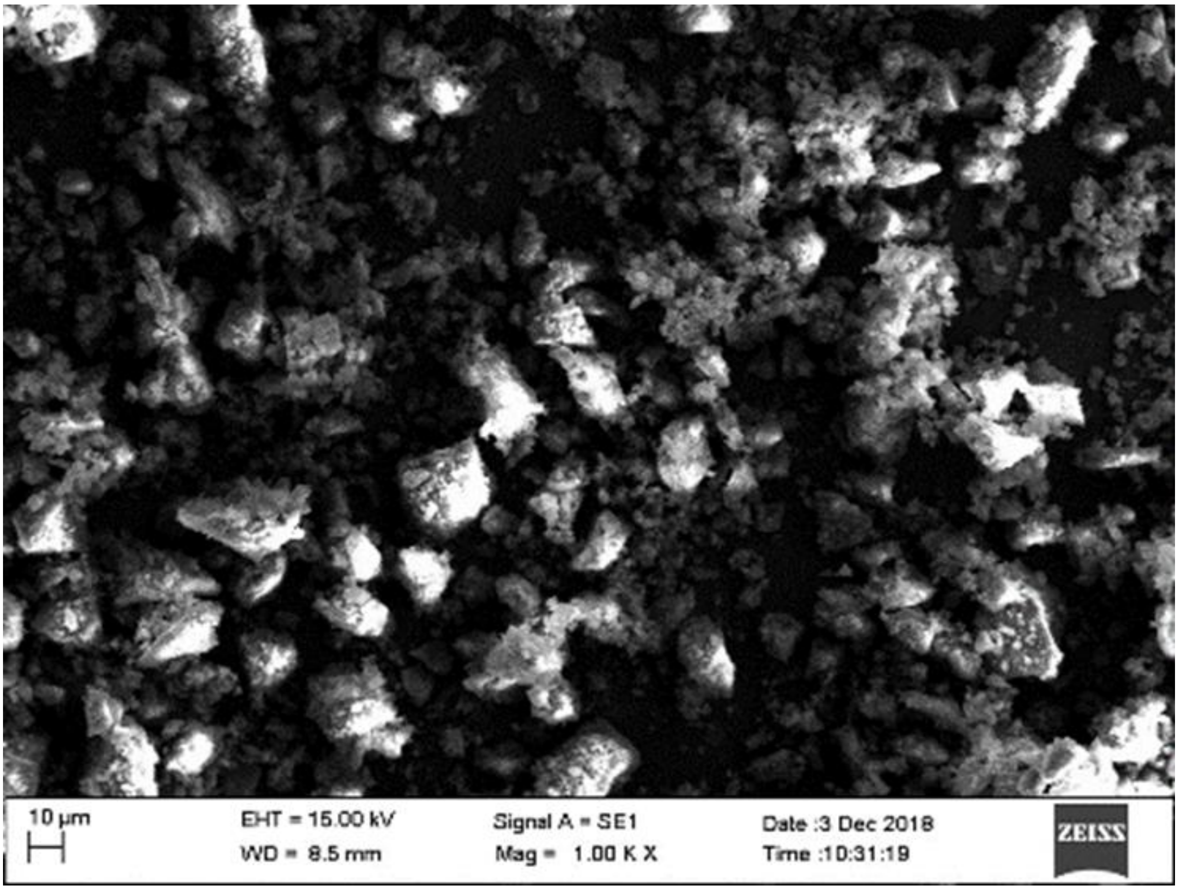

Figure S42: 100 µm resolution SEM picture of insoluble β-CDP (entry 9 of Table 5)*

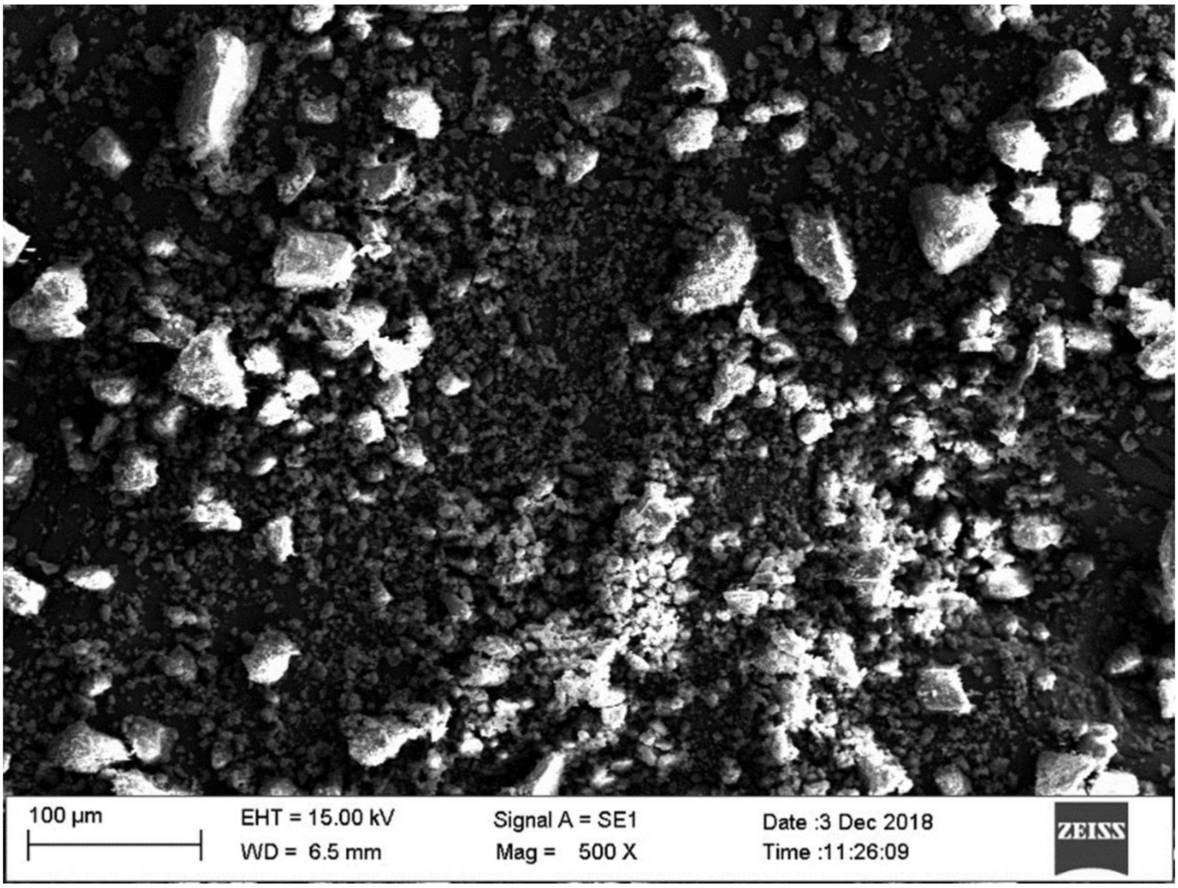

Figure S43: 100 µm resolution SEM picture of insoluble β-CDP (entry 10 of Table 5)*

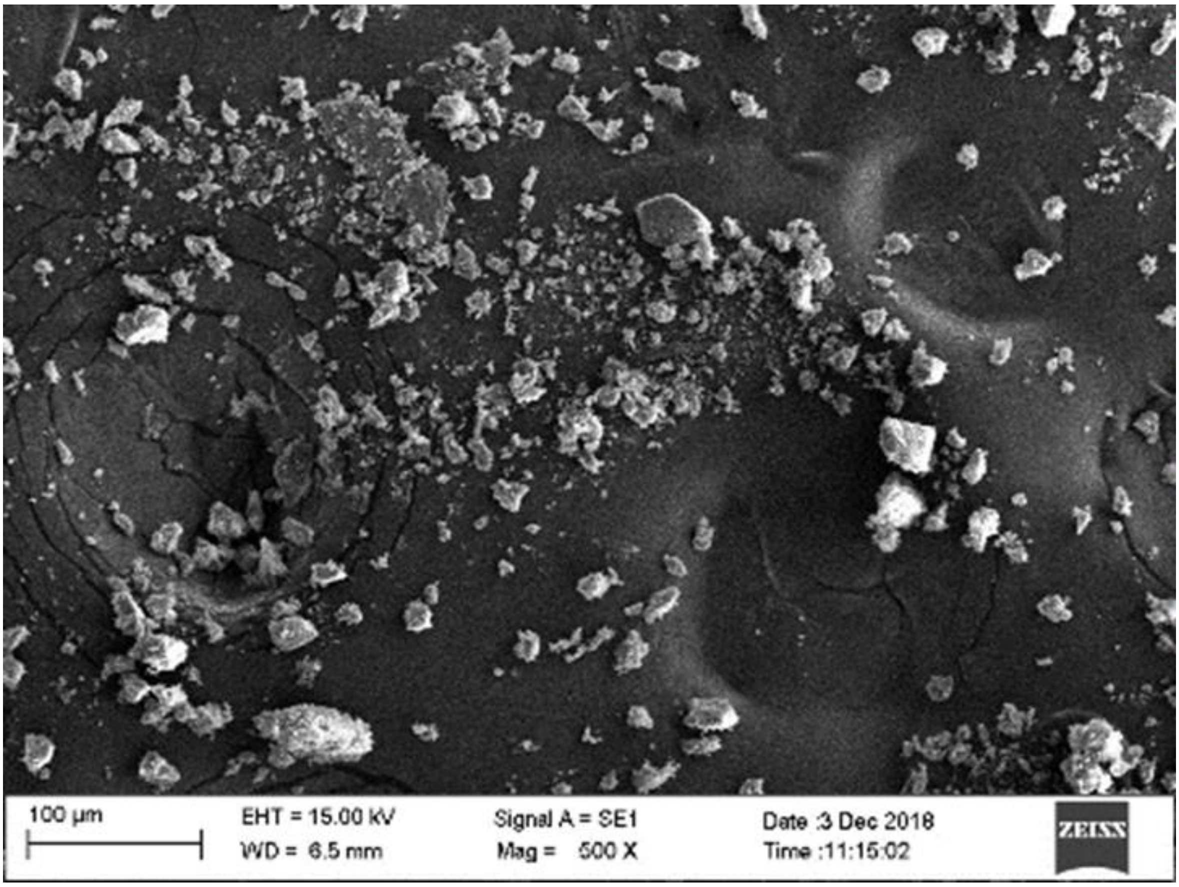

Figure S44: 100 µm resolution SEM picture of insoluble γ-CDP (entry 12[*] of Table 5)

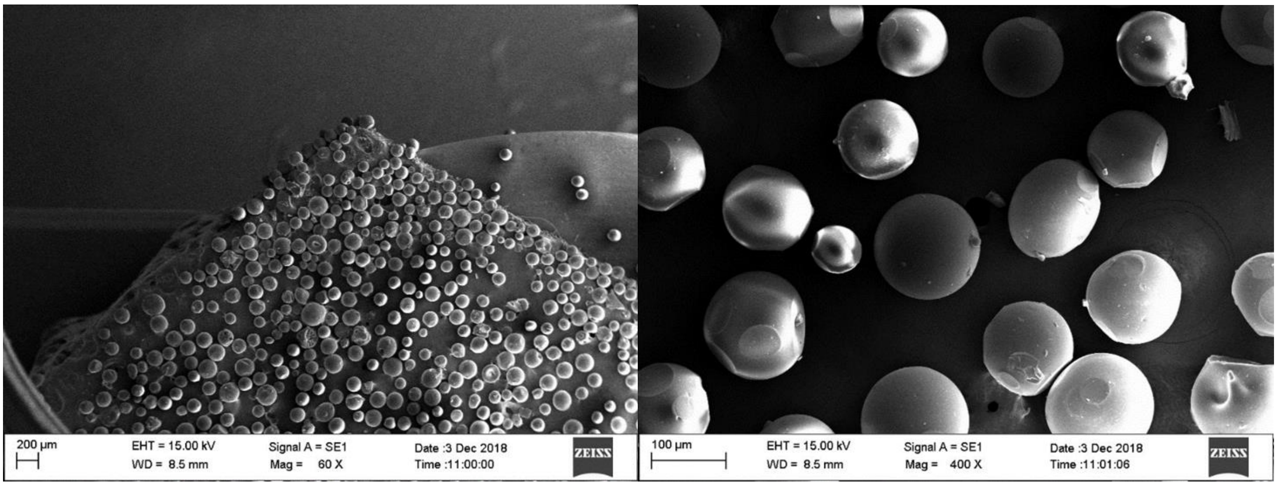

Figure S45: 200 and 100 µm resolution SEM picture of insoluble β-CDP bead (entry 14[*] of Table 5)

[*]Entry labels are corresponding to the body text, not the SI.

HPxCD_LJ10 repeated 4 (Combined)

Effective Diameter: 1867.9 nm
Polydispersity: 0.005
Avg. Count Rate: 271.4 kcps
Sample Quality: 3.5
Elapsed Time: 00:01:30

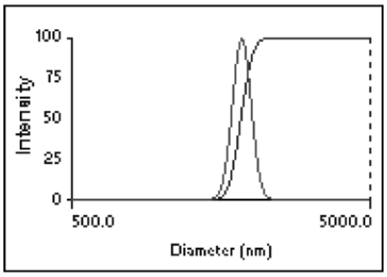

Lognormal

Run	Eff. Diam. (nm)	Half Width (nm)	Polydispersity	Sample Quality
1	1752.7	123.9	0.005	2.7
2	1973.4	139.5	0.005	7.3
3	1887.9	133.5	0.005	0.0
Mean	1871.3	132.3	0.005	3.3
Std. Error	64.2	4.5	0.000	2.1
Combined	1867.9	132.1	0.005	3.5

Figure S46: Particle size analysis of insoluble β-CDP (entry 9 Table 5)*

HPxCD_LJ17 insol rptd (Combined)

Effective Diameter: 2881.1 nm
Polydispersity: 0.005
Avg. Count Rate: 81.1 kcps
Sample Quality: 0.0
Elapsed Time: 00:03:00

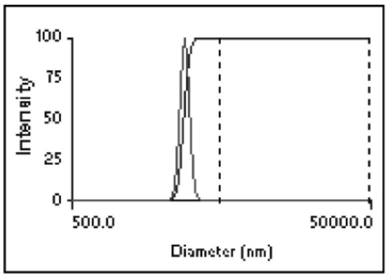

Lognormal

Run	Eff. Diam. (nm)	Half Width (nm)	Polydispersity	Sample Quality
1	2632.3	186.1	0.005	0.0
2	2676.9	1038.9	0.151	0.0
3	3231.5	228.5	0.005	0.0
Mean	2846.9	484.5	0.054	0.0
Std. Error	192.7	277.5	0.049	0.0
Combined	2881.1	203.7	0.005	0.0

Figure S47: Particle size analysis of insoluble β-CDP (entry 10 Table 5)*

HPxCD_LJ11 (bCD*H2O) 2 (Combined)

Effective Diameter: 1713.6 nm
Polydispersity: 0.005
Avg. Count Rate: 592.0 kcps
Sample Quality: 4.2
Elapsed Time: 00:01:30

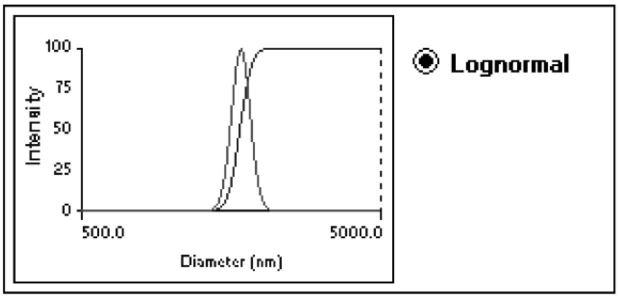

Run	Eff. Diam. (nm)	Half Width (nm)	Polydispersity	Sample Quality
1	1672.2	118.2	0.005	8.5
2	1781.4	126.0	0.005	0.0
3	1694.2	119.8	0.005	9.5
Mean	1716.0	121.3	0.005	6.0
Std. Error	33.3	2.4	0.000	3.0
Combined	1713.6	121.2	0.005	4.2

Figure S48: Particle size analysis of insoluble β-CDP (entry 11 Table 5)*

HPxCD_LJ12 repeated 2 (Combined)

Effective Diameter: 1775.4 nm
Polydispersity: 0.005
Avg. Count Rate: 470.0 kcps
Sample Quality: 3.6
Elapsed Time: 00:01:30

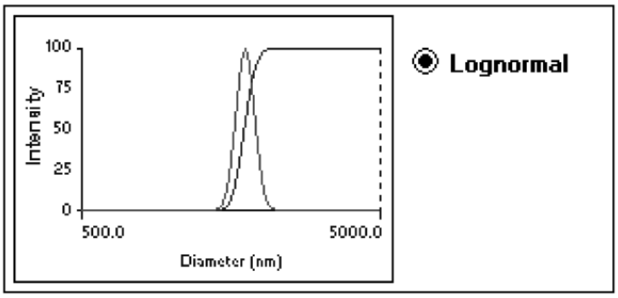

Run	Eff. Diam. (nm)	Half Width (nm)	Polydispersity	Sample Quality
1	1670.5	118.1	0.005	8.2
2	1817.7	128.5	0.005	4.9
3	1888.8	133.6	0.005	0.0
Mean	1792.3	126.7	0.005	4.4
Std. Error	64.3	4.5	0.000	2.4
Combined	1775.4	125.5	0.005	3.6

Figure S49: Particle size analysis of insoluble γ-CDP (entry 12 Table 5)*

HPxCD_LJ13 repeated 2 (Combined)

Effective Diameter: 2160.2 nm
Polydispersity: 0.005
Avg. Count Rate: 383.3 kcps
Sample Quality: 0.0
Elapsed Time: 00:01:30

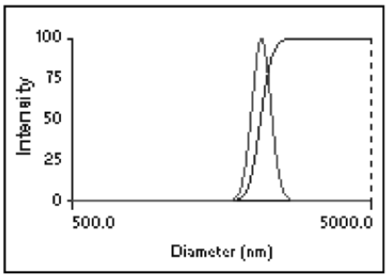

Run	Eff. Diam. (nm)	Half Width (nm)	Polydispersity	Sample Quality
1	2112.6	149.4	0.005	0.0
2	2063.3	145.9	0.005	0.0
3	2216.5	156.7	0.005	0.0
Mean	2130.8	150.7	0.005	0.0
Std. Error	45.1	3.2	0.000	0.0
Combined	2160.2	152.7	0.005	0.0

Figure S50: Particle size analysis of insoluble γ-CDP (entry 13[*] Table 5)

[*]Entry labels are corresponding to the body text, not the SI.
